# Hydrogen Bonds Dominate Brønsted Acid Sites in Zeolite SSZ‐42: A Classification of Their Diversity

**DOI:** 10.1002/anie.202109313

**Published:** 2021-11-30

**Authors:** Christian Schroeder, Stacey I. Zones, Michael Ryan Hansen, Hubert Koller

**Affiliations:** ^1^ Institut für Physikalische Chemie Westfälische Wilhelms-Universität Corrensstr. 28/30 48149 Münster Germany; ^2^ Center of Soft Nanoscience Westfälische Wilhelms-Universität Busso-Peus-Str. 10 48149 Münster Germany; ^3^ Chevron Energy Technology Company Richmond CA 94804 USA

**Keywords:** Catalysis, Defects, Solid-state NMR, Synthesis, Zeolite

## Abstract

The zeolite catalyst SSZ‐42 shows a remarkable high abundance (≈80 %) of hydrogen‐bonded Brønsted acid sites (BAS), which are deshielded from the ^1^H chemical shift of unperturbed BAS at typically 4 ppm. This is due to their interaction with neighboring oxygen atoms in the zeolite framework when oxygen alignments are suitable. The classification and diversity of hydrogen bonding is assessed by DFT calculations, showing that oval‐shaped 6‐rings and 5‐rings allow for a stronger hydrogen bond to oxygen atoms on the opposite ring side, yielding higher experimental chemical shifts (δ (^1^H)=6.4 ppm), than circular 6‐rings (δ(^1^H)=5.2 ppm). Cage‐like structures and intra‐tetrahedral interactions can also form hydrogen bonds. The alignment of oxygen atoms is expected to impact their role in the stabilization of intermediates in catalytic reactions, such as surface alkoxy groups and possibly transition states.

## Graphical abstract

The zeolite catalyst SSZ‐42 shows a high abundance of hydrogen bonding of Brønsted acid sites. These occur mainly due to the existence of 5‐ and 6‐rings, with suitable alignment of oxygen atoms, in which the acidic bridging OH groups can interact with oxygen atoms on the opposite side of the rings. ^1^H NMR spectroscopic components of the acid sites overlap with coexisting defect silanol groups that are also hydrogen‐bonded.

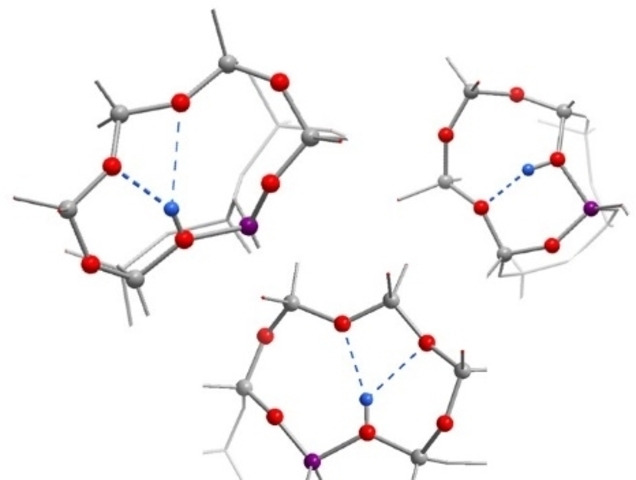

Zeolites belong to the most successful inorganic materials in large‐scale applications, including ion‐exchange, adsorption/separation processes, and heterogeneous catalysis.^[1]^ Their hydrothermal syntheses often rely on organic structure‐directing agents (OSDAs), typically quaternary ammonium cations, filling the pore system in the as‐made material.^[2]^ The charge of the OSDAs can be balanced by AlO_4/2_^−^ tetrahedra in the 3D tetrahedral aluminosilicate framework. However, a perfect match of the OSDA packing density and the negative charge due to Al insertion into the framework is usually not attained, and additional negative charge centers (SiO^−^ siloxy groups) are required. Such framework defects, stabilized by hydrogen‐bonded SiOH groups, are found in all‐silica zeolites when synthesis gels at high pH are used with OH^−^ ions as the mineralizing agent.^[3]^ The removal of OSDAs by calcination results in zeolites with catalytically active Brønsted acid sites (BAS). Hydrogen‐bonded defect silanol groups in calcined zeolites can have ^1^H NMR chemical shifts in the same range, where ^1^H signals from BAS are typically found in calcined zeolites.[^3f^, ^4^] BAS can also form hydrogen bonds when suitable O−O distances and mutual alignments of O atoms exist.^[5]^ A hydrogen bond is defined^[6]^ by a ^1^H chemical shift that is deshielded from the unperturbed BAS at typically 4 ppm. Here, we employ a combination of solid‐state NMR methods and DFT calculations of zeolite cluster models for zeolite SSZ‐42.^[7]^ A high abundance of hydrogen bonds was predicted for the BAS in SSZ‐42.^[5]^

Zeolite SSZ‐42, which is isostructural to MCM‐58^[8]^ and ITQ‐4,^[9]^ has the IFR framework type.^[10]^ It was prepared by a published procedure,^[11]^ and the structural integrity is documented by X‐ray powder diffraction and ^29^Si MAS NMR spectroscopy (Figure S1). The ^27^Al NMR data shown in Figure S2 confirm the presence of tetrahedral Al in the zeolite framework (chemical analysis: Si/Al atomic ratio of 22, that is 1.39 Al per unit cell). SSZ‐42 has 4 distinct tetrahedral (T) sites,^[7c]^ and the T3 position is most likely not occupied by Al (Figure S2). This differs from a previous study on ITQ‐4, where mainly T1 and T2, along with some T3 and less T4 positions are occupied.^[12]^ There are 2.09 OSDA (N‐benzyl‐1,4‐diazabicyclo[2.2.2]octane cations) and 0.49  Na^+^ cations per unit cell (chemical analysis). Therefore, the Al content is insufficient to provide the necessary charge balance, and defect sites are required.

The ^1^H MAS NMR data of as‐made SSZ‐42 (Figure 1) show a signal at 9.8 ppm for SiOH groups, donating hydrogen bonds to a SiO^−^ siloxy group.^[3a]^ These charged defect sites contribute to the charge compensation of the OSDA, which is responsible for various ^1^H NMR peaks below 9 ppm for aromatic and aliphatic protons.

**Figure 1:**
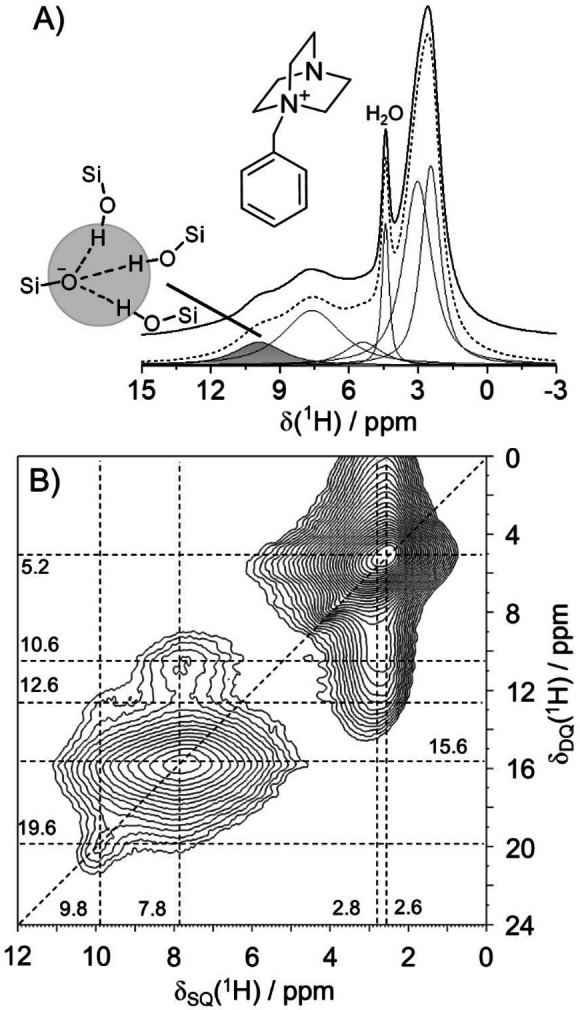
A) ^1^H MAS NMR spectrum of as‐made SSZ‐42 with a Si/Al ratio of 22, and the structural sketch illustrates the hydrogen‐bonded framework defects (^1^H signal near 10 ppm); the ^1^H peak at 4.5 ppm is assigned to H_2_O near Na^+^ cations,^[3a]^ B) ^1^H DQ‐SQ MAS NMR spectrum of as‐made SSZ‐42.

The peak at a single‐quantum chemical shift of δ_SQ_=9.8 ppm shows a ^1^H double‐quantum‐single‐quantum, DQ‐SQ, NMR signal^[13]^ at δ_DQ_=19.6 ppm (2×9.8 ppm; auto‐correlation). In fact, there are three such silanols, as can be verified in the ^1^H triple‐quantum‐single‐quantum (TQ‐SQ) correlation peak at δ_TQ_=29.4 ppm in Figure S3. This finding agrees well with the results for various other zeolites.[^3b^, ^3e^] The same three protons are also spatially close to the methylene groups at the charge center of the quaternary ammonium cation (cross‐correlation: δ_SQ_=2.8/9.8 ppm; δ_DQ_=12.6 ppm, Figure 1B). The other correlations in Figure 1B are due to ^1^H dipolar interactions within the OSDA. Therefore, the positive charge center of the OSDA and the negatively charged defects in the zeolite framework are spatially close as expected.[^3b^, ^3d^, ^3e^, ^14^] It is thus important to examine whether these defect sites contribute to the calcined material.

The ^1^H MAS NMR data of calcined and dehydrated SSZ‐42 (Figures 2A and S4) show signals at 3.9 and 5.2 ppm along with a broad component at 8 ppm. The components below 3 ppm are due to silanol groups without hydrogen bonds. The presence of water can be ruled out by a thorough sample preparation, and the NMR data do not indicate any presence of water.[^5^, ^15^] The broad line at 8 ppm disappears in the ^1^H spin‐echo MAS NMR spectrum (Figure 2B), uncovering another component at 6.4 ppm. On the other hand, the projection of the ^1^H DQ‐SQ MAS NMR spectrum yields signal intensities at 3—5 ppm and 6—10 ppm (Figure 2C). These signals are assigned to hydrogen‐bonded silanol dyads, which show the same ^1^H DQ‐SQ cross‐correlation pattern (Figure S5) as previously observed for SSZ‐70 and ZSM‐5.[^3f^, ^4a^, ^15b^] A protonation of the defect site sketched in Figure 1A would result in a silanol tetrad, which is not seen in the ^1^H DQ‐SQ MAS NMR pattern of Figure S5. Thus, two of the expected four silanols apparently have condensed. This is confirmed by the reduced ^29^Si NMR signal intensities of Q^3^ groups in calcined SSZ‐42 compared to the as‐made zeolite (Figure S1).

**Figure 2:**
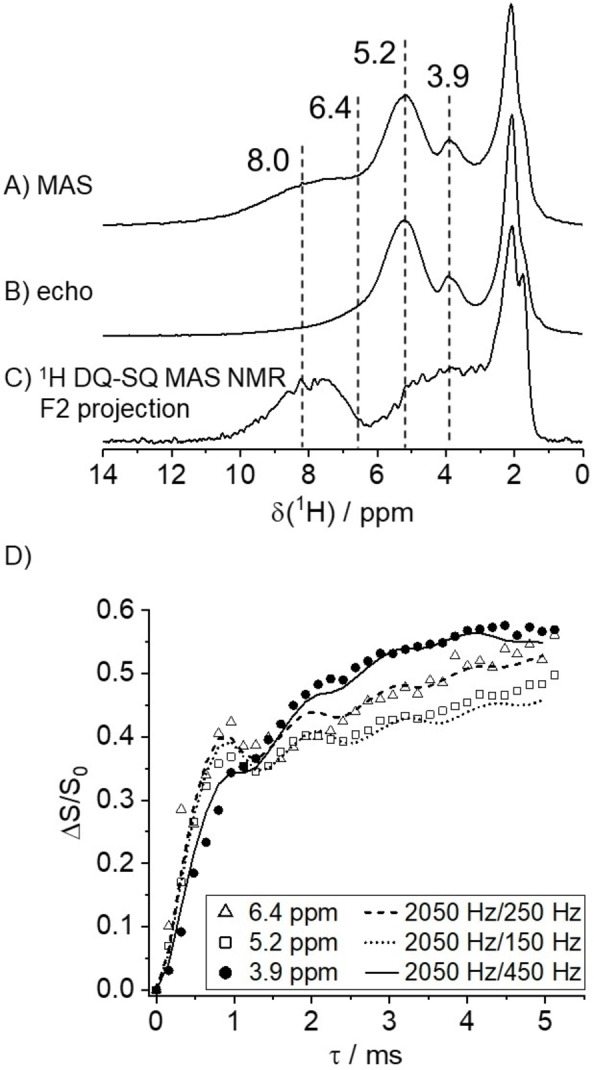
A) ^1^H MAS NMR, B) spin‐echo ^1^H MAS NMR (*τ*=1.28 ms) and C) F2 projection of the ^1^H DQ‐SQ MAS NMR spectrum for dehydrated H‐SSZ‐42. D) ^1^H{^27^Al} REAPDOR evolution curves of ^1^H signals for BAS at 3.9, 5.2 and 6.4 ppm (symbols) with corresponding SIMPSON simulations (lines). Note that the simulation for 3.9 ppm is corrected for the overlap of SiOH groups (Figure S6B).

Notably, the components at 5.2 and 6.4 ppm are not observed in the ^1^H DQ‐SQ MAS NMR data, which indicates that these are isolated from other protons. We assign these peaks to hydrogen‐bonded BAS.

The defect silanol groups and BAS can be separated by the analyses of T_2_(^1^H) relaxation times (Figure S6A). The BAS protons at 5.2 and 6.4 ppm have long spin‐spin relaxation times of 7.4±0.2 and 4.5±0.2 ms, whereas the silanol groups at 8 ppm yield a much shorter T_2_(^1^H) of 0.33±0.01 ms, which is in good agreement with previous observations for ZSM‐5.^[15b]^ Interestingly, the component at 3.9 ppm (unperturbed BAS without hydrogen bonding) exhibits a biexponential T_2_(^1^H) relaxation. Therefore, this line originates from two different species, that is defect silanol groups (the dyad observed in the ^1^H DQ‐SQ MAS NMR spectrum, see above, T_2_(^1^H)=0.98±0.12 ms) and BAS (T_2_(^1^H)=4.4±0.2 ms).

The ^1^H{^27^Al} rotational‐echo adiabatic‐passage double‐resonance (REAPDOR) technique^[16]^ uses the difference, Δ*S*, between the ^1^H spin‐echo signal intensities (Figure 2B) without (*S*_0_) and with a ^27^Al radiofrequency pulse that can cause a change in the ^27^Al spin state, thus altering the ^1^H‐^27^Al dipolar evolution. This leads to the reduced intensity, *S*, when the ^1^H and ^27^Al spins are spatially proximal, making this method suitable to unequivocally distinguish BAS protons from silanol groups. The normalized intensity differences, Δ*S*/*S*_0_, depend on the spin‐echo evolution time, *τ*, (Figure 2D). ^1^H‐^27^Al distances can be obtained,[^5^, ^17^] using numerical simulations.^[18]^ The initial slope of such a “REAPDOR curve” is dominated by the shortest ^1^H‐^27^Al distance within the BAS. The slope that evolves beyond ≈1 ms evolution time can be simulated by an additional ^27^Al spin, outside the BAS, with a longer ^1^H‐^27^Al distance.[^5^, ^15c^] By this means, the analyses of the ^1^H NMR lines at 5.2 and 6.4 ppm yields typical dipolar coupling constants of 2050 Hz, corresponding to a ^1^H‐^27^Al distance of 2.48 Å within the BAS, and 150 Hz (6.0 Å) or 250 Hz (5.0 Å) outside. The shortest distance at 2.48 Å is typical for a Brønsted acid site.[^5^, ^15b^, ^17d^, ^19^]

The analysis of the line at 3.9 ppm for BAS in Figure 2D is more complicated, because the signal is weak and overlaps with that from defect silanol groups. The SiOH groups contribute markedly to the spin‐echo intensity, *S*_0_, thus damping the Δ*S*/*S*_0_ data primarily at short *τ* times. Therefore, the analysis of the 3.9 ppm line takes this into account (for details see Figure S6B) and the ^1^H{^27^Al} REAPDOR evolution of the line at 3.9 ppm can be also simulated with a dipolar coupling constant of 2050 Hz (2.48 Å). Interestingly, it shows a second slope above 1 ms evolution time with a dipolar coupling constant of 450 Hz that corresponds to a ^1^H‐^27^Al distance of 4.1 Å. The fact that this only occurs for the ^1^H component at 3.9 ppm and not for the other BAS is intriguing and will be explained below.

The ^27^Al quadrupolar coupling constants are 17.5 MHz near the ^1^H components at 3.9 and 5.2 ppm and 13 MHz near the protons at 6.4 ppm as summarized in Figure S7. These values are typical for BAS,^[20]^ thus bolstering the assignments of these ^1^H NMR components, ruling out that other Al−OH species contribute to the ^1^H NMR spectrum.^[5]^

We conclude that the ^1^H NMR components at 3.9, 5.2 and 6.4 ppm can clearly be assigned to BAS, and the line at 3.9 ppm overlaps with a signal originating from defect silanol groups. These ^1^H NMR lines of BAS in SSZ‐42 were also found for zeolite ITQ‐4.[^17c^, ^21^] ITQ‐4 is made in the presence of F^−^ as mineralizing agent, and thus has a very low level of defects.[^9^, ^22^] In contrast, SSZ‐42 is made with hydroxide ions, OH^−^, and has additional framework defects compared to ITQ‐4. This explains, why the broad ^1^H components at 8 ppm and the one overlapping at 3.9 ppm are absent in ITQ‐4.[^17c^, ^21^]

The typical ^1^H chemical shift of BAS is 4 ppm, and the larger shifts of 5.2 and 6.4 ppm are due to the predicted hydrogen bonds for the IFR framework type.^[5]^ Hydrogen‐bonded BAS were also identified in ZSM‐5 with a similar chemical shift as the component at 6.4 ppm.^[5]^ However, it is remarkable that the most intense line for BAS in SSZ‐42 is at 5.2 ppm, which is in contrast to other zeolites where a line near 4 ppm for unperturbed BAS is typically dominant.^[4b]^ This unusual observation for SSZ‐42 is in accord with our recent prediction that 63 % of the possible bridging OH groups in this material should form a hydrogen bond.^[5]^ In fact, the ^1^H MAS NMR analysis indicates that even more, that is 80±5 %, of the BAS (5.2 and 6.4 ppm signal intensities, Figure 2B) are forming hydrogen bonds. This finding will now be elaborated by DFT cluster calculations of SSZ‐42.

Figure 3A illustrates the principles of hydrogen bond predictions in zeolites.^[5]^ The alignment angle, *κ*, is defined between the direction of the Si−O−Al bisector and the O−O connecting line. Oxygen pairs with distances between 2.7 and 4.0 Å have been proposed to be the most likely candidates for hydrogen bond formation for κ angles below 50°.^[5]^ This is not a sharp boundary, but outside of this range hydrogen bonds tend to be less likely and/or weaker. We used the structure of calcined SSZ‐42^[7c]^ to extract starting models for the DFT cluster calculations and for predicting possible hydrogen bonds. The structural details before and after DFT geometry optimizations are listed in Table S1. These data confirm that the hydrogen bond formation decreases the O⋅⋅⋅O distances and κ angles.

**Figure 3:**
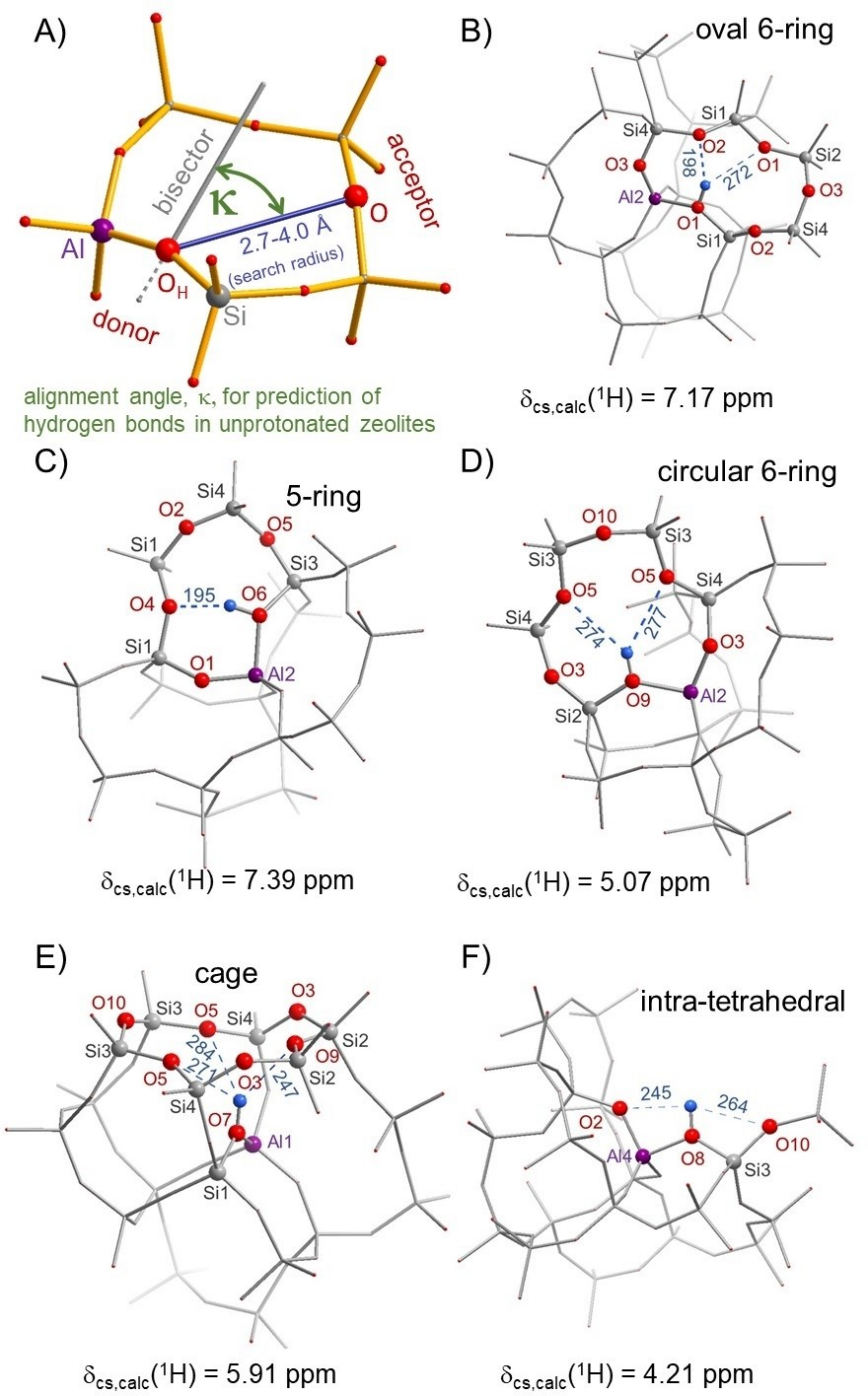
A) definition of *κ* angle and B–F) excerpts of DFT cluster geometry optimizations (PBE‐D3/def2‐TZVP) for selected hydrogen bonds of bridging OH groups in zeolite SSZ‐42. All 16 possible hydrogen positions are listed in the Supporting Information. Hydrogen bond lengths are given in pm.

Five typical situations are shown in Figure 3 for which hydrogen bonds are found in SSZ‐42, exhibiting a ^1^H NMR shift above 4 ppm. The ^1^H chemical shifts are a direct indicator for the strength of the hydrogen bonds and number of hydrogen bond acceptors. All 16 DFT models are shown in Figure S8. Figure 3B shows an oval‐shaped 6‐ring, and it allows the formation of a relatively strong hydrogen bond (198 pm distance) along with a second, much weaker one at 272 pm. Together these result in a calculated ^1^H NMR chemical shift of 7.17 ppm. Oval 6‐rings are abundant in SSZ‐42 (Figures S8A, B, E), and together with the 5‐ring in Figure 3C, they are good candidates for explaining the broad experimental ^1^H component at 6.4 ppm. We note that the DFT calculations tend to overestimate stronger hydrogen bonding to some extent, and that these calculated ^1^H chemical shifts are slightly higher than their experimental values.^[3f]^ When a 6‐ring is shaped circularly, then weaker hydrogen bonds are formed (Figures 3D, S8F, H, I, N) that can explain the ^1^H line at 5.2 ppm. A similar circular 6‐ring model was found for zeolite Y, where weak hydrogen bonds of the BAS at O2 and O3 in the sodalite cages exist with a ^1^H chemical shift of 4.5 ppm.[^4b^, ^15a^, ^23^] The cooperative influence of several weak hydrogen bonds can also add up to a sizeable ^1^H NMR shift, as shown for the cage model in Figure 3E. Intra‐tetrahedral interactions, as shown in Figure 3F and previously proposed,^[24]^ have a small to moderate ^1^H chemical shift effect (Figure 3F: 4.21 ppm, Figure S8K: 4.84 ppm, Figure S8L: 5.17 ppm). As these differences are still remarkable, given the similar H⋅⋅⋅O distances in these models, we propose that the orientation of oxygen electron lone pairs in the hydrogen bond acceptor should also be important for such intra‐tetrahedral hydrogen bonds. This can be best appreciated in Figure S8J (for Al3), where potential H‐bond acceptor atoms in Si−O−Si or Al−O−Si bridges are oriented unfavourably in other directions and not towards the hydrogen atom, yielding the lowest calculated ^1^H chemical shift of 3.78 ppm in this study. Note that the T3 position is not occupied by Al. Therefore, this site does not contribute to the observed signals.

In summary, hydrogen bonding is ubiquitous in zeolite SSZ‐42, yielding strong ^1^H NMR signals at 6.4 and 5.2 ppm, and an unperturbed BAS was only found for Al4/O2 (3.95 ppm, Figure S8M) by DFT model calculations. This H location is assigned to the ^1^H NMR line at 3.9 ppm. The DFT model shows a distance of 420 pm between the BAS proton and the T2 position in the same 6‐ring, which can explain the observed ^1^H‐^27^Al distance of 4.1 Å. The H‐T2 distances are longer for the hydrogen‐bonded BAS, located at the other oxygen atoms, which explains why the experimentally observed distance of 4.1 Å is only found for the proton at 3.9 ppm. This assignment suggests that T4 and T2 are both occupied by Al.

Increasing (calculated) deprotonation energies (DPE) generally correlate with higher ^1^H chemical shifts (Figure S9), although there is some scatter and the hydrogen bond in the 5‐ring (Figure 3C) violates the correlation substantially. We anticipate that local framework strain and flexibility also affects the DPE.^[23]^ Although, the protons will be highly mobile at high temperatures,^[25]^ where catalytic reactions are usually carried out, the alignment of the oxygen atoms within small 5‐ and 6‐rings is likely to impact the catalytic reaction. For example, the formation of surface alkoxy groups will force the bridging oxygen atom in an sp^2^‐type hybridization with the alkoxy group in one plane with Si−O−Al. This is expected to be sterically hindered, when the oxygen atoms have a small alignment angle, thus making certain reactions unfavorable. Hence, the identification of hydrogen‐bonded acid sites, as shown in this work, is suggested to be a general indicator for the alignment of oxygen atoms. In contrast, the absence of hydrogen‐bonded BAS indicates that the oxygen atoms are preferably oriented towards the zeolite pore.

## Conflict of interest

The authors declare no conflict of interest.

## Supporting information

As a service to our authors and readers, this journal provides supporting information supplied by the authors. Such materials are peer reviewed and may be re‐organized for online delivery, but are not copy‐edited or typeset. Technical support issues arising from supporting information (other than missing files) should be addressed to the authors.

Supporting Information
