# Supplementary material for: Hydrogen Bonds Dominate Brønsted Acid Sites in Zeolite SSZ‐42: A Classification of Their Diversity
Source: Angew Chem Int Ed Engl. 2021 Nov 30;61(3):e202109313. doi: 10.1002/anie.202109313 (PMC9299800; doi:10.1002/anie.202109313)
Supplement: Supplementary file 1 — Supporting Information [file ANIE-61-0-s001.pdf]

Angewandte Chemie
Eine Zeitschrift der Gesellschaft Deutscher Chemiker

Supporting Information

Hydrogen Bonds Dominate Brønsted Acid Sites in Zeolite SSZ-42: A Classification of Their Diversity

*C. Schroeder, S. I. Zones, M. R. Hansen, H. Koller**

SUPPORTING INFORMATION

Contents

A Experimental Section 3
A.1 Syntheses 3
A.2 Solid State NMR and XRD 3
A.3 DFT Calculations 3
B Supplementary Experimental Data 4
B.1 X-ray Powder Diffraction and ^{29}Si MAS NMR 4
B.2 ^{27}Al MAS NMR 5
B.3 ^{1}H TQ-SQ MAS NMR of As-Made SSZ-42 6
B.4 ^{1}H MAS and DQ-SQ MAS NMR Data of Calcined SSZ-42 7
B.5 $T_2(^{1}H)$ Relaxation Times and Correcting $^{1}H\{^{27}Al\}$ READPOR Evolution Due to the Presence of Defects 8
B.6 $^{1}H\{^{27}Al\}$ Offset READPOR Data 9
C Cluster Models of Acid Site Locations and Calculated ^{1}H Chemical Shifts. 10
D Tables 19
References 21
E Cartesian Atomic Coordinates (in Å) for the Zeolite Clusters 21

A Experimental Section

A.1 Syntheses

SSZ-42 was synthesized with Al in the framework, using zeolite Y as a starting material, according to the published procedure.[1] The as-made SSZ-42 was calcined in air by heating with a ramp of 1 K/min to 823 K (with 2 h held at 393 K in between) and held at 823 K for 12 h. After cooling to room temperature, 0.2 g of calcined SSZ-42 was ion-exchanged with NH_4^+ by treatment in 12 mL 1M NH_4^+ acetate solution under shaking (240 rounds per minute) for 5 days. NH_4^+-SSZ-42 was dried at 323 K overnight and then deammoniated by placing it in a borosilicate glass tube under vacuum ($<10^{-2}$ mbar) and heating with following temperature program: Room temperature to 383 K with 2 K/min, hold for 2 h, heating to 723 K with 2 K/min, hold for at least 12 h. After that, samples were flame-sealed under vacuum, and stored inside the ampules until needed.
The absence of water, NH_4^+ and AlOH groups in ^{1}H MAS NMR spectra was verified by a combination of NMR experiments, as described in our earlier works.[2] Water and NH_4^+ would give much longer H-Al distances than determined for the BAS in SSZ-42. Al-OH groups would be associated with Al moieties in extra-framework or partially from the framework extracted Al. These will be less strained in their local coordination geometry than the trigonally distorted AlO_4 tetrahedra for a BAS. Therefore, the ^{27}Al quadrupolar coupling constant of Al-OH species would be smaller than observed here for the BAS.
The Si/Al chemical analyses was carried out by ICP-AES at Galbraith Laboratories, Knoxville, TN, and CHN analysis was performed at the Chevron Corp. research center in Richmond, CA.

A.2 Solid State NMR and XRD

All solid-state NMR experiments were performed at a Bruker Avance I spectrometer with a 4mm HX double resonance probe operating at a magnetic flux density of 9.4 T, utilizing software Topspin 1.3. Hydrated samples (as-made SSZ-42, calcined SSZ-42) were transferred into 4mm ZrO_2 rotors with Kel-F caps under ambient conditions, while dehydrated H-SSZ-42 was transferred in a dry nitrogen atmosphere to prevent rehydration. [2a] The MAS rate was set to 12.5 kHz in all cases.

^{1}H MAS NMR spectra were acquired with a π/2-pulse of 4 µs with a corresponding radio frequency field strength of 62.5 kHz, and referencing was executed with adamantane (δ(^{1}H) = 1.78 ppm). The recycle delay was set to 15 s. ^{1}H DQ-SQ and TQ-SQ MAS NMR spectra utilized one rotor period of the back-to-back pulse scheme each for excitation and reconversion of multi quantum coherences, with different phase cycles to select either double or triple quantum coherences.

^{27}Al MAS NMR spectra were referenced against 1M $Al(NO_3)_3$ solution (δ(^{27}Al) = 0 ppm), and the excitation pulse was set to 0.56 µs (π/18-pulse of the liquid standard, corresponding to an rf field strength of 50.0 kHz). ^{27}Al MQMAS experiments were used in the z-filtered variant with short pulses of 4 µs and 1.5 µs for the excitation and reconversion of triple quantum coherences, respectively, and a read-out pulse of 10 µs. Shearing of the data was performed with Topspin 4.0.6.

^{1}H{^{27}Al} REAPDOR experiments were acquired with π/2- and π-pulses of 4 and 8 µs, respectively, on the ^{1}H channel with an rf field strength of 62.5 kHz and an rf field strength of 50.0 kHz on the ^{27}Al channel. The adiabatic pulse length was kept at 1/9 of the rotor period (8.89 µs in case of a MAS rate of 12.5 kHz), and points at discrete evolution times at $2nT_r$ were acquired. For ^{27}Al offset-dependent ^{1}H{^{27}Al} REAPDOR experiments, the irradiation frequency was shifted at intervals of 150 kHz between 0 and 3 MHz offset, and at intervals of 300 kHz for higher offset values. The 8th REAPDOR point (nT_r = 16, 1.28 ms for a MAS rate of 12.5 kHz) was used.

^{29}Si MAS NMR spectra were recorded with a π/2-pulse length of 5 µs corresponding to an rf field strength of 50 kHz, and were referenced against tetrakis-trimethylsilylsilane (δ(^{29}Si) = -9.8 ppm)

All NMR spectra were processed using Topspin 4.0.6, and deconvolution of signals was done with dmfit 2015. [3] Numerical simulations of ^{1}H{^{27}Al} REAPDOR evolution curves and ^{27}Al offset-dependent ^{1}H{^{27}Al} REAPDOR profiles was executed with SIMPSON Version 4.1. [4] Distributions of ^{27}Al CQ values from ^{27}Al offset-dependent ^{1}H{^{27}Al} REAPDOR measurements were determined by creating a library of simulations with ^{27}Al CQ values between 0 and 30 MHz and adding these in Origin2019 according to monomodal or bimodal Gaussian distribution functions similar to our recent work. [5]

Powder X-ray diffractograms were acquired on a Stoe StadIP (Cu-$K_{\alpha1}$ 1.5406 Å) by placing samples in 0.3 mm capillaries and measurement under rotation.

A.3 DFT Calculations

We performed DFT calculations on cluster models of SSZ-42, where one of the four crystallographic T sites is occupied by Al, surrounded by SiO_4 tetrahedra within a radius of 9 Å. Additional peripheral SiO_4 tetrahedra were added, as needed, to avoid dangling SiO_3 groups (Q^1), which was achieved for example by completing the small 4-, 5- and 6-rings. The dangling Si-O bonds at the cluster boundaries (for Q^2 and Q^3) were replaced by Si-H bonds. For geometry optimization, the functional PBE[6] together with Grimmes D3 dispersion correction [7] and a triple-ζ basis set (def2-TZVP) was used.[8] The thus obtained cartesian coordinates were then scaled by a factor of 1.01 (which was the scaling factor with the lowest cluster energy) to take into account that the Al-O distances in the cluster center need more space than Si-O bonds. After an initial optimization of the H atomic positions at the cluster boundaries, these H coordinates were then frozen while the other atomic positions were fully optimized.
The cluster was then recalculated with a hybrid meta GGA functional (PW6B95)[9], while keeping the triple-ζ basis set (def2-TZVP), for the calculation of ^{1}H and ^{27}Al chemical shifts. The ^{27}Al chemical shift was reference to a cluster extracted from a Si-rich chabazite structure, [10] and tetramethylsilane was used as the ^{1}H reference. All DFT calculations were performed with Turbomole 7.4.[11]

B Supplementary Experimental Data

B.1 X-ray Powder Diffraction and ^{29}Si MAS NMR

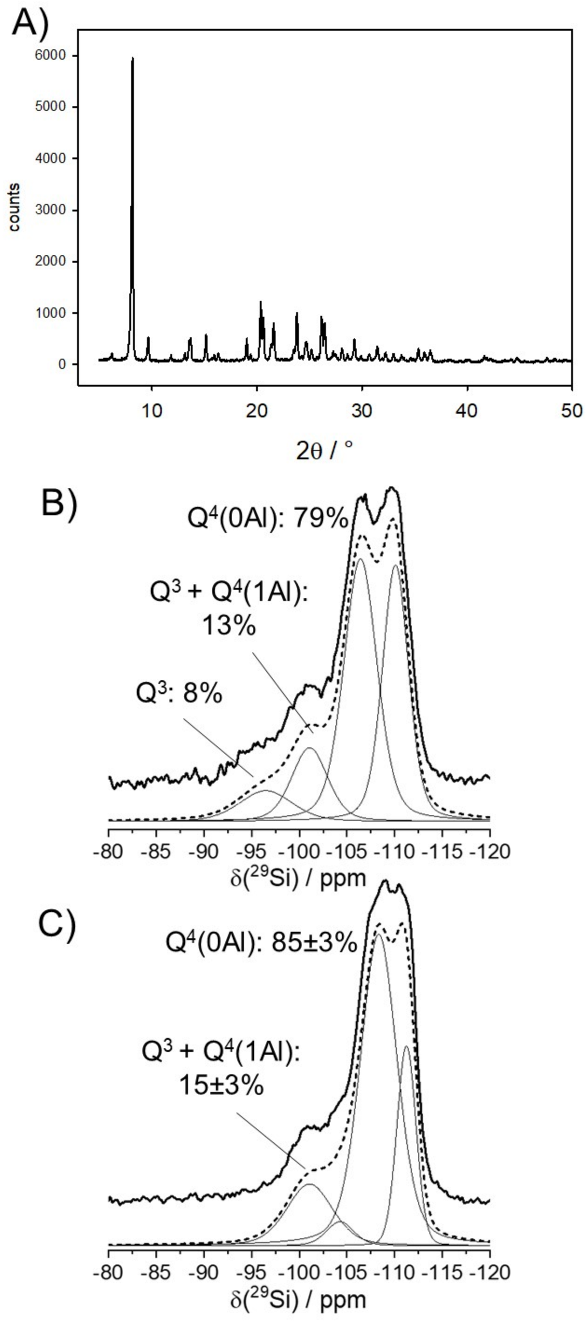

Figure S1:
A) X-ray powder diffraction pattern of calcined Al-SSZ-42, B) ^{29}Si MAS NMR spectrum of hydrated as-made and C) calcined Al-SSZ-42. We note that the small X-ray diffraction peak at 6.20° is due to a minor impurity (~ 4% relatively to the maximum peak for SSZ-42 at 8.15°) of zeolite Y starting material remaining after the hydrothermal synthesis.

B.2 ^{27}Al MAS NMR

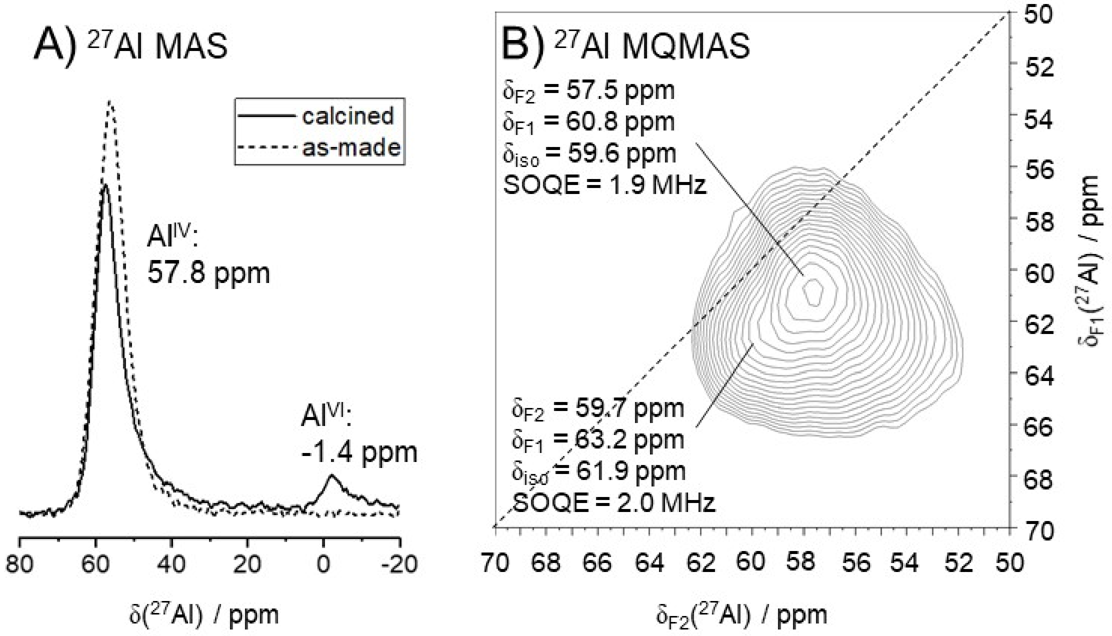

Figure S2: A) ^{27}Al MAS NMR spectra of hydrated as-made and hydrated calcined Al-SSZ-42. B) ^{27}Al MQMAS NMR spectrum of hydrated calcined Al-SSZ-42. The calculated ^{27}Al chemical shifts are 58.83 ppm, 59.95 ppm, 63.93 ppm and 60.66 ppm for Al1, Al2, Al3 and Al4, respectively. Therefore, the calculated ^{27}Al chemical shift for the Al3 position differs by more than 2 ppm from the observed chemical shifts, from which we conclude that the T3 position is not occupied by Al.

B.3 ^{1}H TQ-SQ MAS NMR of As-Made SSZ-42

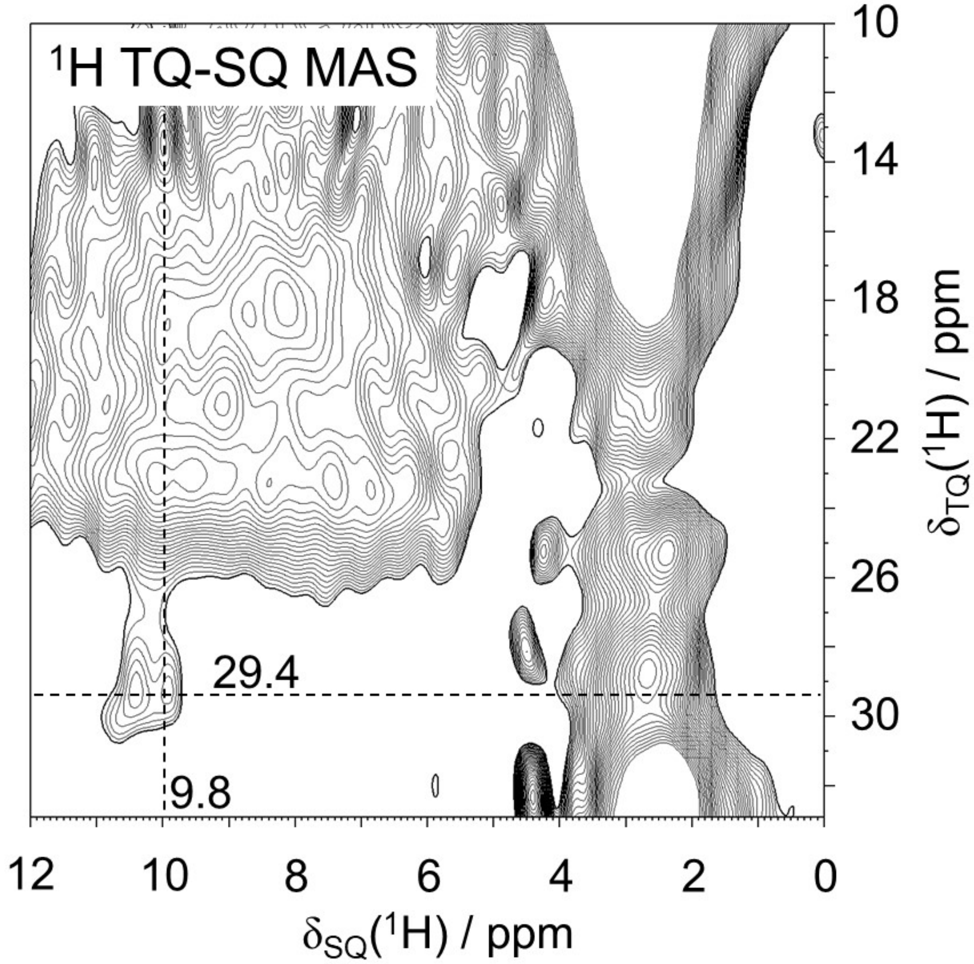

Figure S3: ^{1}H TQ-SQ MAS NMR spectrum of as-made SSZ-42. The auto-correlation peak at $\delta_{TQ}(^1H)$ = 29.4 ppm demonstrates the presence of [3 SiOH : 1 SiO]$^-$ clusters.

B.4 ¹H MAS and DQ-SQ MAS NMR Data of Calcined SSZ-42

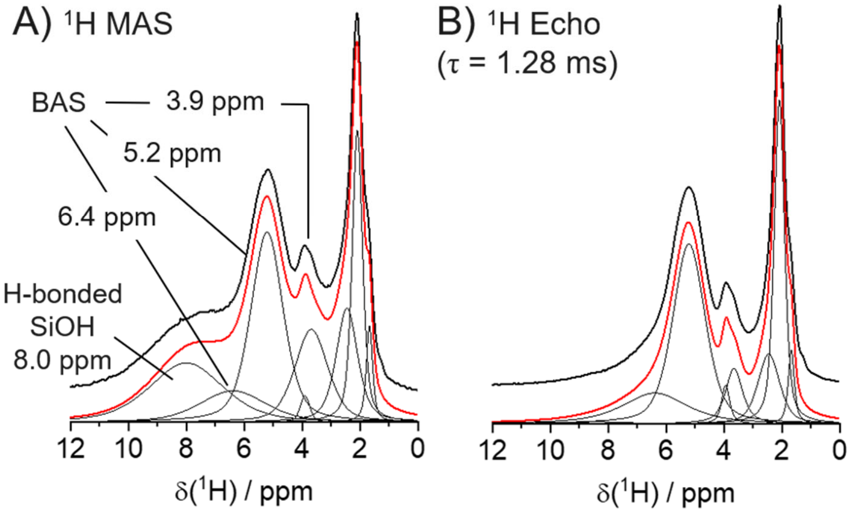

Figure S4: A) ^1H MAS NMR and B) spin-echo ^1H MAS NMR spectrum with simulations of dehydrated H-SSZ-42 from Figures 2A,B.

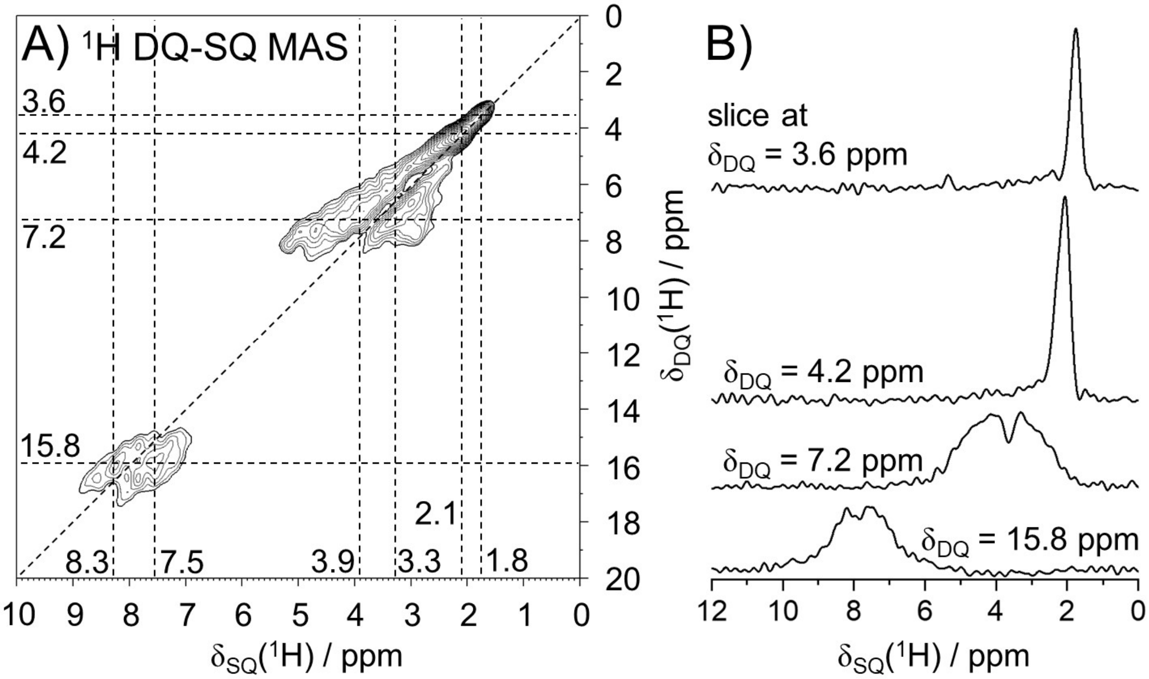

Figure S5: A) ^1H DQ-SQ MAS NMR spectrum of calcined and dehydrated H-SSZ-42. B) Slices along the SQ dimension at δ_{DQ} = 3.6, 4.2, 7.2 and 15.8 ppm.

B.5 T_2(^{1}H) Relaxation Times and Correcting ^{1}H{^{27}Al} READPOR Evolution Due to the Presence of Defects

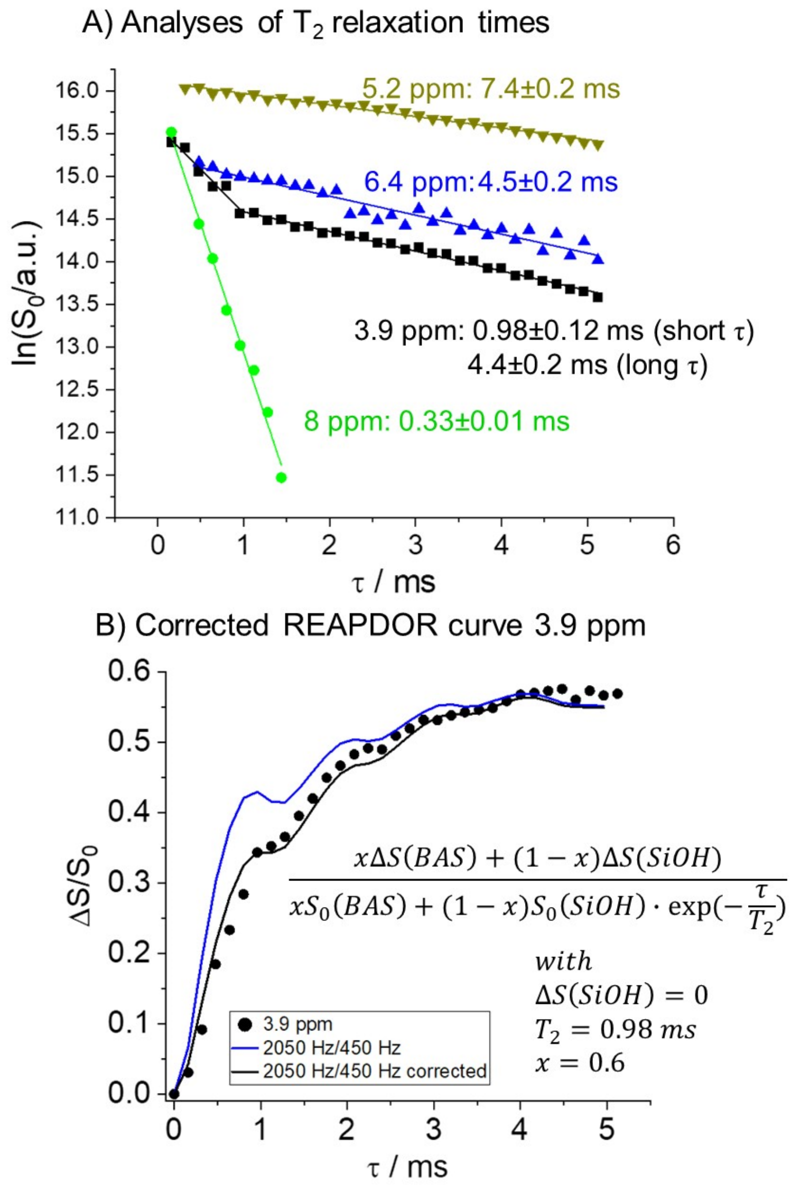

$$\frac{x\Delta S(BAS) + (1-x)\Delta S(SiOH)}{xS_0(BAS) + (1-x)S_0(SiOH)\cdot \exp(-\frac{\tau}{T_2})}$$

Figure S6: A) Analyses of T_2 relaxation times of ^{1}H in dehydrated SSZ-42 and B) Simulation of ^{1}H{^{27}Al} REAPDOR evolution curve for the ^{1}H component at 3.9 ppm, considering the overlapping signal intensities from BAS and SiOH groups. The simulation model assumes that the ^{1}H{^{27}Al} REAPDOR effect is zero (ΔS(SiOH)=0), and the ^{1}H spin-echo MAS NMR intensity of the silanol groups, S_0(SiOH), decays with a T_2 relaxation time constant of 0.98 ms according to part A).

B.6 $^{1}H\{^{27}Al\}$ Offset READPOR Data

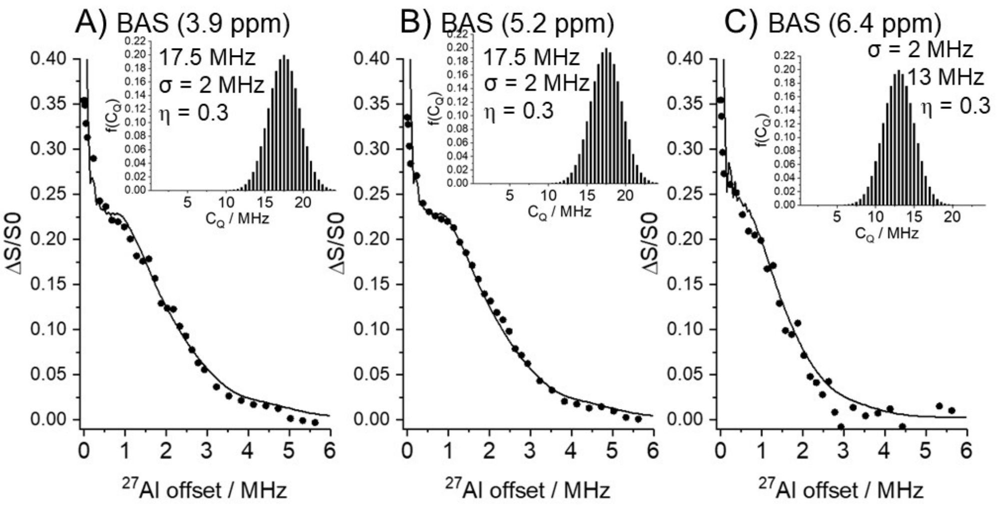

Figure S7: ^{27}Al offset-dependent $^{1}H\{^{27}Al\}$ REAPDOR profiles for BAS. $\Delta S/S_0$ values are mapped for the range of ^{27}Al frequencies covering the ^{27}Al spectral width stepwise, while keeping the ^{1}H spin-echo NMR evolution time constant ("offset REAPDOR") at 1.28 ms. As the data must be symmetric to zero offset, only one side was acquired with high data point resolution. This method allows to acquire very broad lines and yields ^{27}Al quadrupolar coupling constants, C_Q, of 17.5 MHz for ^{27}Al near the protons at 3.9 and 5.2 ppm and 13 MHz near the protons at 6.4 ppm. (A) ^{1}H NMR component at 3.9 ppm and hydrogen-bonded BAS at (B) 5.2 ppm and (C) 6.4 ppm (C) of dehydrated H-SSZ-42. The solid lines are simulations with the parameters given in the figures. Insets show obtained distributions (σ) of ^{27}Al C_Q values.

C Cluster Models of Acid Site Locations and Calculated ^{1}H Chemical Shifts.

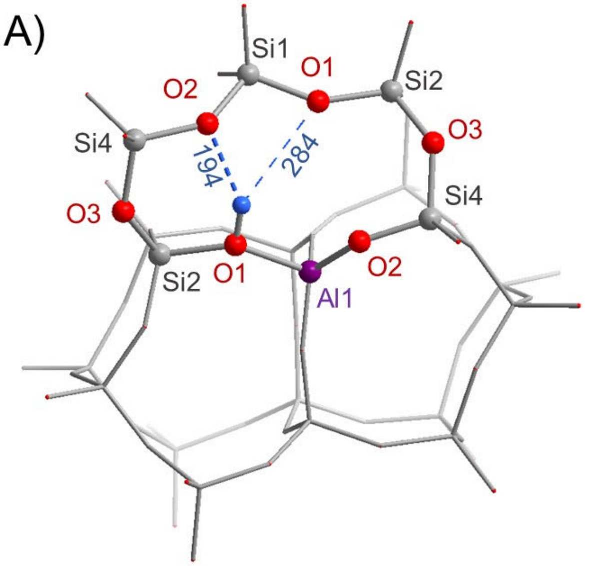

$\delta_{cs,calc}(^1H)$ = 7.06 ppm

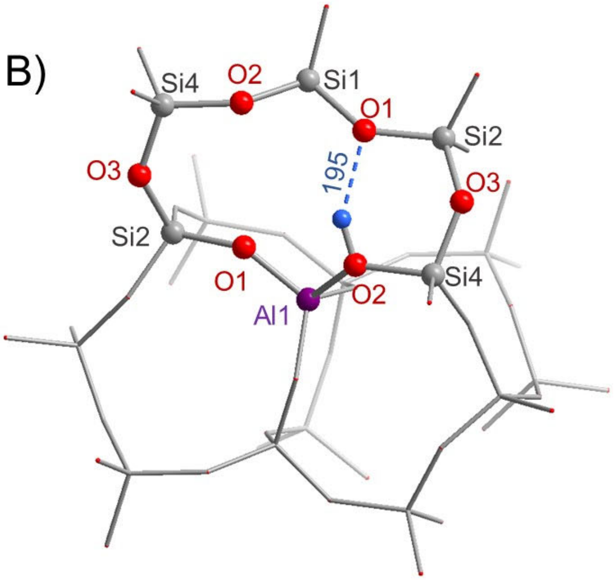

$\delta_{cs,calc}(^1H)$ = 7.06 ppm

C)

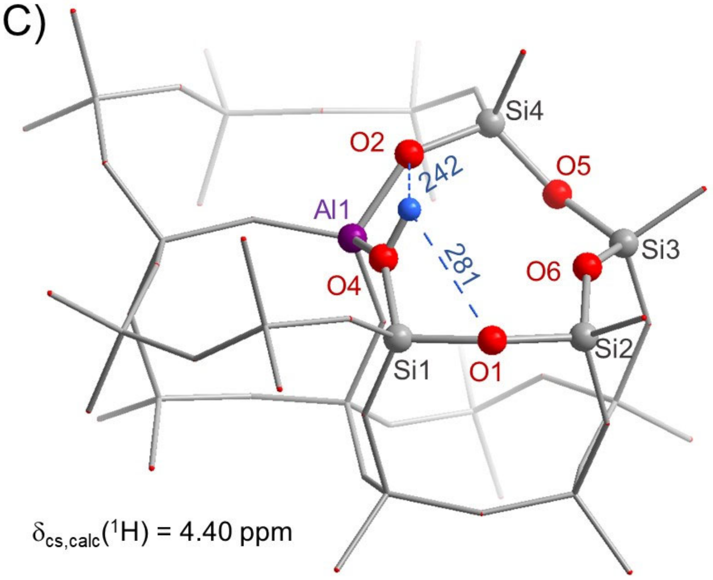

$\delta_{cs,calc}(^1H)$ = 4.40 ppm

D)

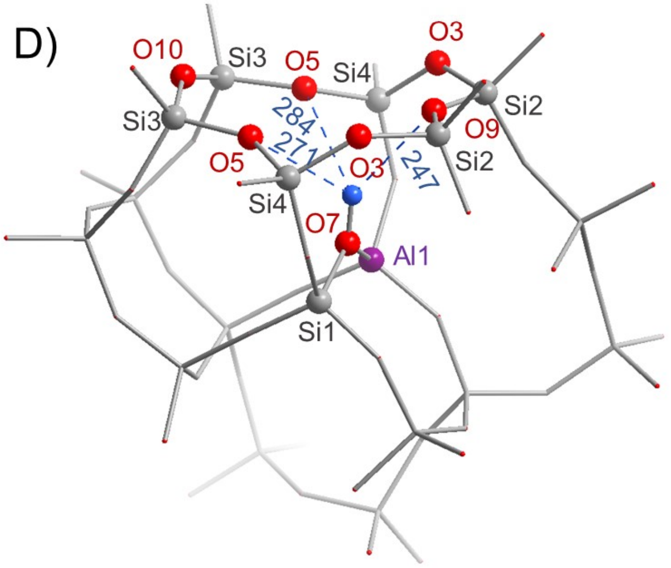

$\delta_{cs,calc}(^1H)$ = 5.91 ppm

E)

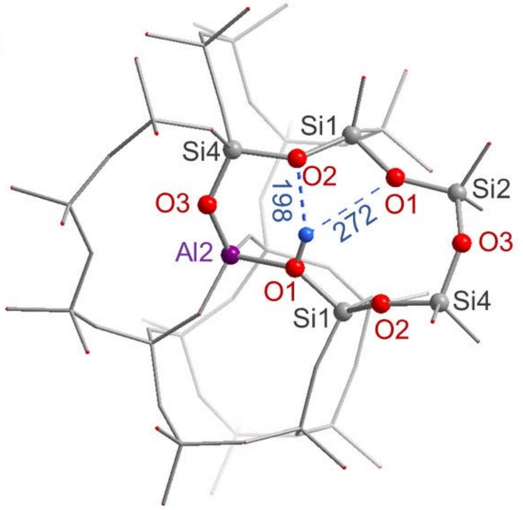

$\delta_{cs,calc}(^1H)$ = 7.17 ppm

F)

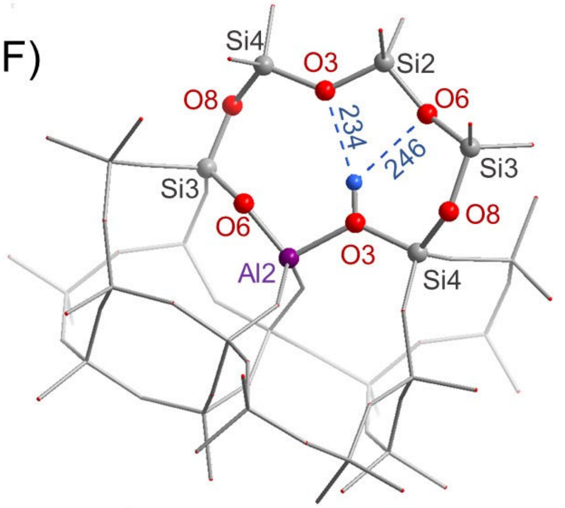

$\delta_{cs,calc}(^1H)$ = 5.81 ppm

G)

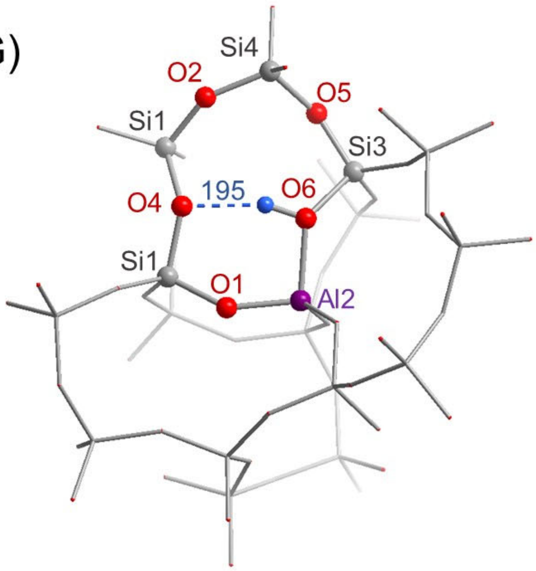

$\delta_{cs,calc}(^1H)$ = 7.39 ppm

H)

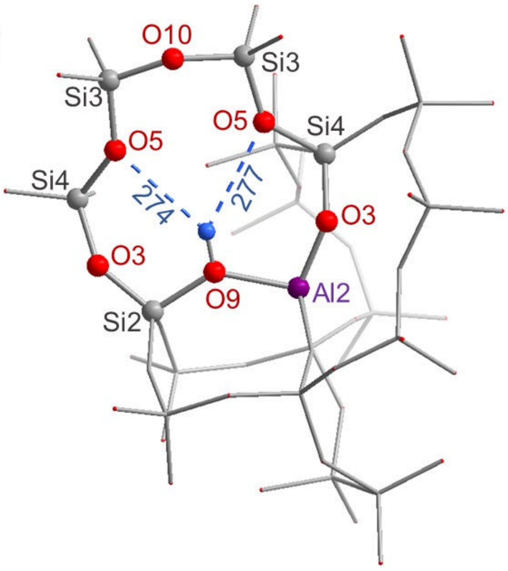

$\delta_{cs,calc}(^1H)$ = 5.07 ppm

I)

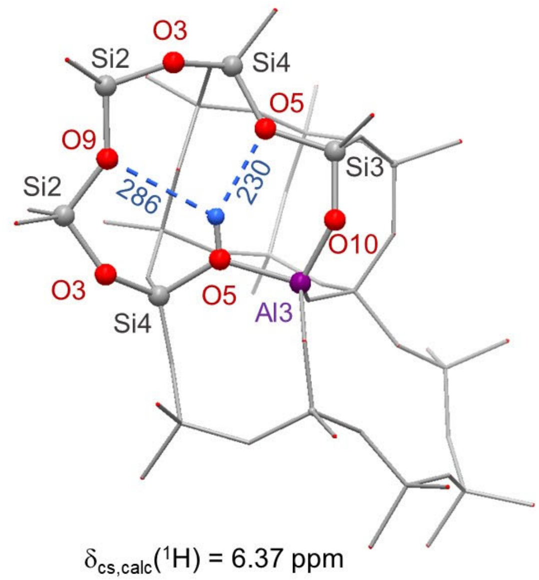

$\delta_{cs,calc}(^{1}H)$ = 6.37 ppm

J)

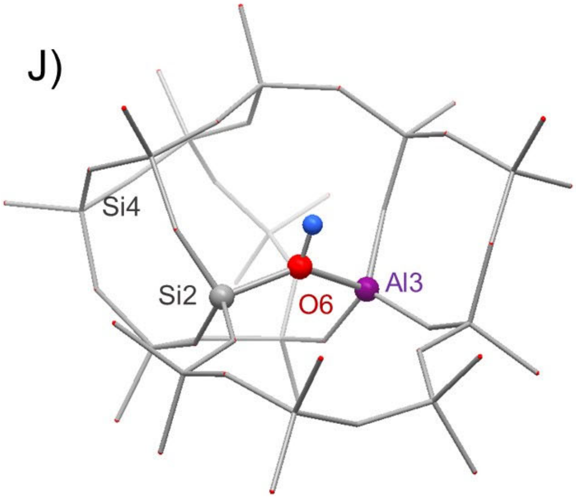

$\delta_{cs,calc}(^{1}H)$ = 3.78 ppm

K)

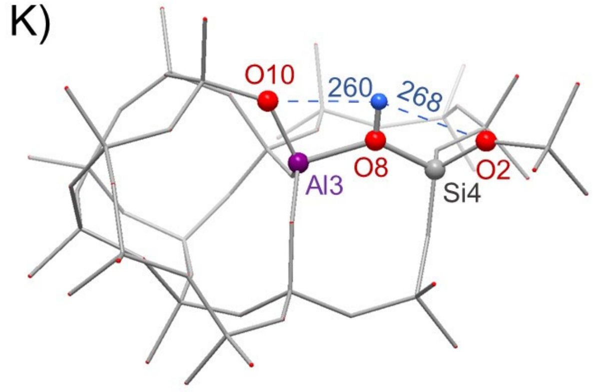

$\delta_{cs,calc}(^1H) = 4.84$ ppm

L)

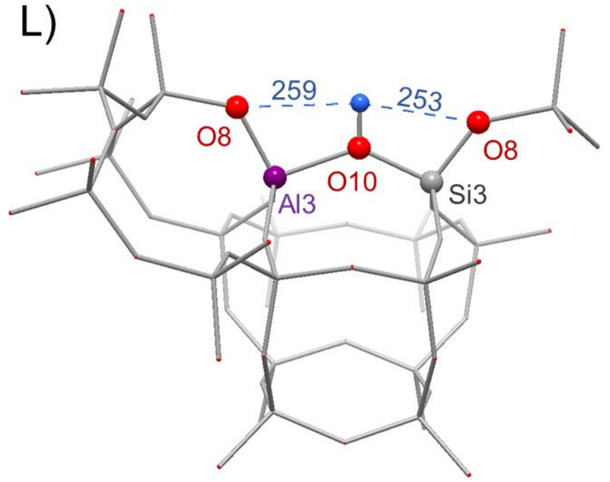

$\delta_{cs,calc}(^1H) = 5.17$ ppm

M)

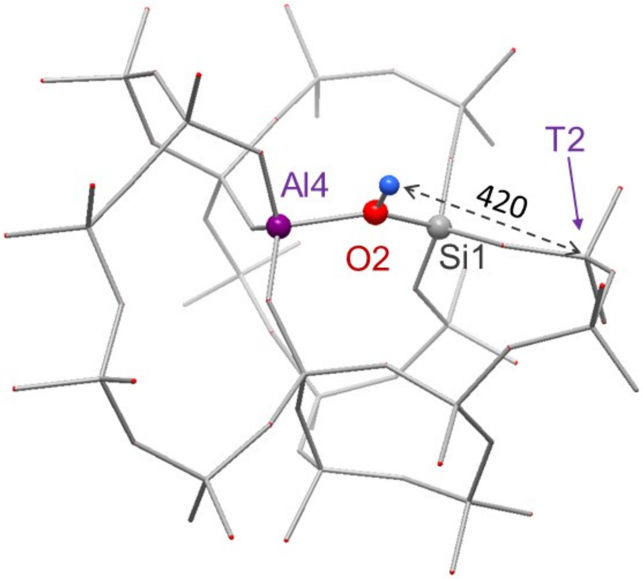

$\delta_{cs,calc}(^1H) = 3.95$ ppm

N)

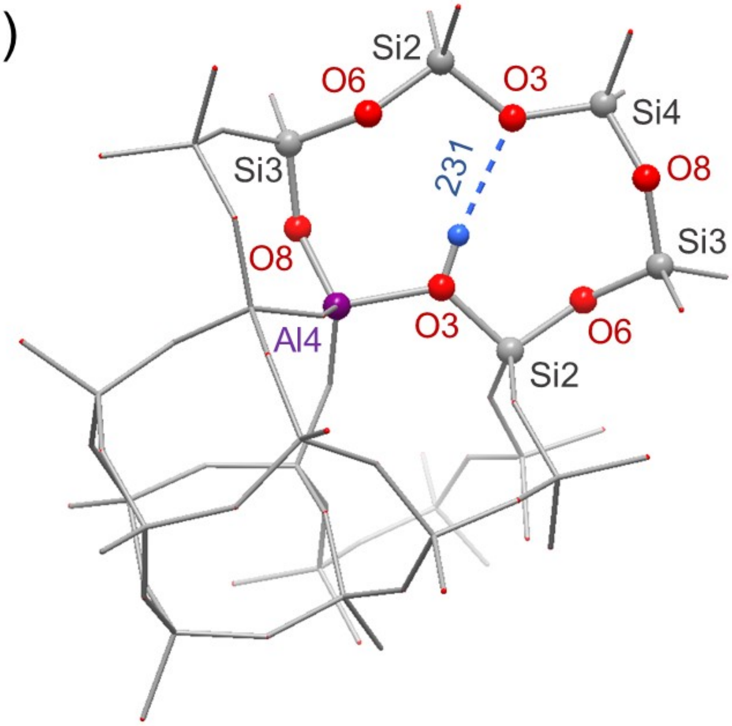

$\delta_{cs,calc}(^1H) = 6.00$ ppm

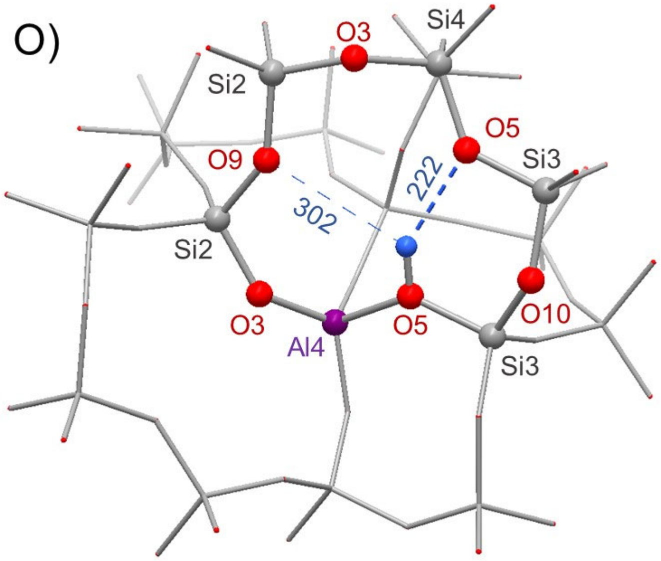

$\delta_{cs,calc}(^1H)$ = 6.18 ppm

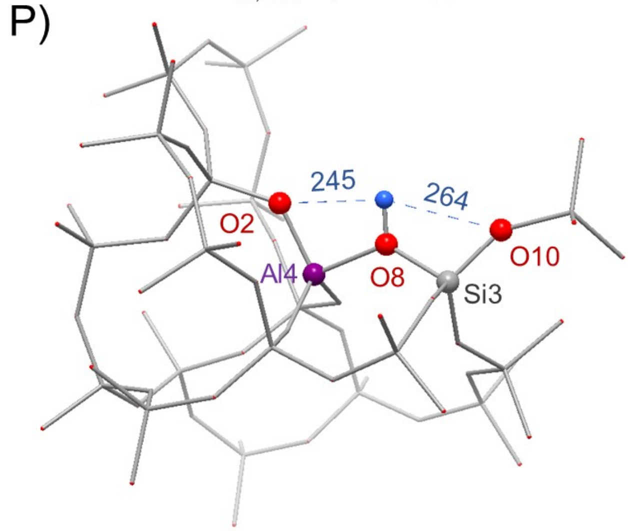

$\delta_{cs,calc}(^1H)$ = 4.21 ppm

Figure S8: Excerpts of DFT cluster models of all 16 possible hydrogen locations of Brønsted acid sites in SSZ-42. Distances are given in pm, and atomic labelling refers to the crystal structure data of SSZ-42, [12] but we note that the symmetry of the clusters is C_1 to allow the atoms to adopt a local distortion of the more symmetric crystal structure (space group C2/m). The full list of cartesian coordinates is given at the end of this Supporting Information, labelled in the heading with the Al position and the oxygen atom that is protonated.

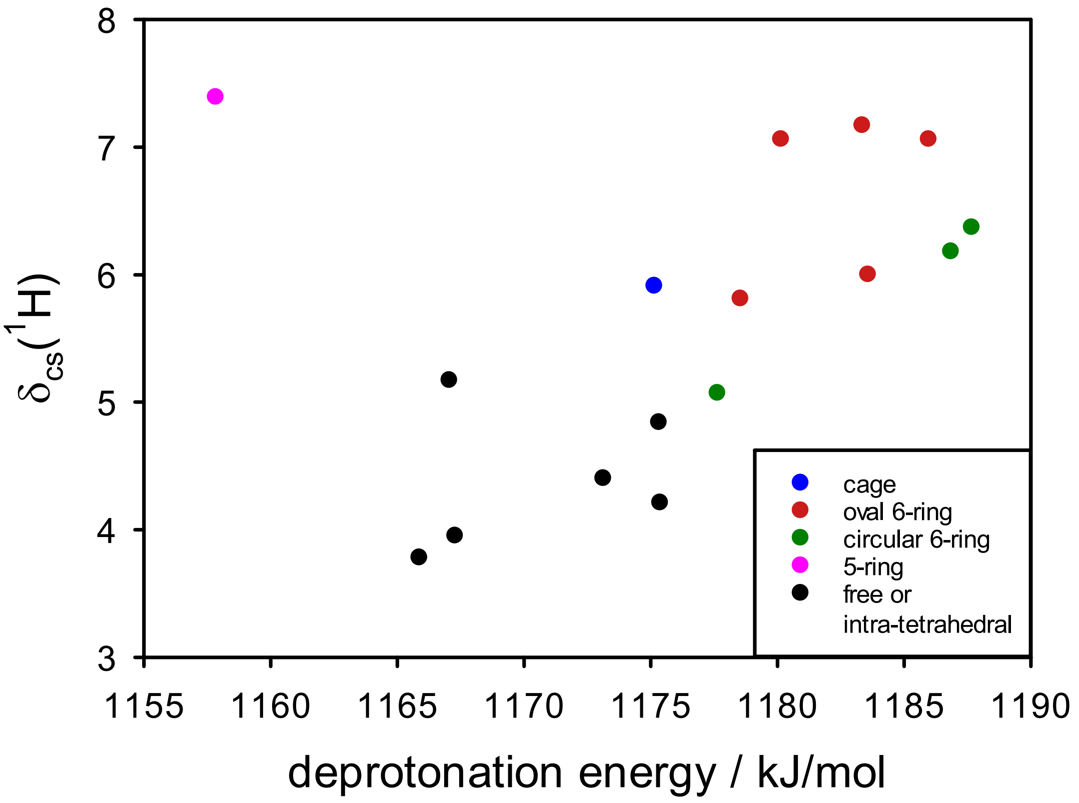

Figure S9: Correlation between deprotonation energy and ^1H chemical shift for the cluster models of Figure S8. The deprotonation energies (DPEs) appear to correlate (with some scattering) with the ^1H chemical shifts for most sites, but the hydrogen bond in the 5-ring (Figure 3C) substantially violates this correlation. This hydrogen bond was not predicted (Table S1), due to a large alignment angle (76.45°). Therefore, we conclude that the DPE is not solely affected by the strength of the hydrogen bond, but also by other parameters, for example the local flexibility and the strain versus relaxation of the zeolite framework upon protonation/deprotonation. This interpretation is based on the suggestion by Eichler et al., who found no correlation between ^1H chemical shifts and deprotonation energies for (smaller) cluster models of zeolite Y and ZSM-5.[13] Their cluster models were a circular 6-ring and a 5-ring.
A higher DPE means higher proton affinity and lower intrinsic acid strength. However, we note that the DPE is a number, which is calculated by a quantum-chemical method, and its exact value depends to some (minor) extent on the computational method. The numbers can thus only be truly compared when the same method was applied. Its significance was described for example in a paper published by J. Sauer, who also described the influence of the dielectric constant of the zeolite.[14] The DPEs shown in this figure are all in within the range, which is expected for strong Brønsted acites, whereas an isolated SiOH group has a comparatively weak intrinsic acidity (DPE on the order of 1400 kJ/mol). We also note that the intrinsic acid strength (characterized by the DPE) does not provide a straightforward means to predict catalytic reactivity. The true acidity for a catalytic reaction should always be seen as a combination of intrinsic acid strength and the solvation (adsorption energy) of the protonated substrate in the zeolite environment. In addition, diffusion may also contribute to the macrokinetics of such a reaction. An example of the complex interplay of all these points can be found for example in the work of Noh, Zones and Iglesia, who described the solvation effects as “confinement”.[15]

D Tables

Table S1: Structural parameters for oxygen atom environments. The crystal structure of SSZ-42[12] was used to define potential hydrogen bond donors, $O_{H,donor}$, and acceptors, $O_{acceptor}$. O···O distances were determined for the (unprotonated) crystal structure, [12] and after DFT geometry optimization of the protonated cluster in this work. Likewise, κ angles from the crystal structure and after DFT geometry optimization are shown along with the calculated ^{1}H chemical shifts. The green color indicates sites for which the two criteria, O···O distance < 4 Å and κ < 50°, suggest that a hydrogen bond would likely form in the protonated structure, which is confirmed by the DFT calculations. The red color indicates sites for which the oxygen alignment model did not predict the formation of hydrogen bonds. Most of these cases are intra-tetrahedral interactions (O4/Al1, O8/Al3, O8/Al4, O10/Al3), in addition to the O2/O1/Al1 site, where a hydrogen bond could form within an oval 6-ring (Figure S7B) with a short initial O···O distance (thus favoring hydrogen bond formation), and the O6/O4/Al2 sites (Figure S7G) for a 5-ring. The latter, O6/O4/Al2, is likely made possible by the second hydrogen bond acceptor, O5, which meets the above criteria.

$O_{H,donor}$	$O_{acceptor}$	Al position	O···O distance (ref. [12]) / Å	O···O distance (DFT) / Å	κ (from data of ref [12]) / °	κ (DFT) / °	δ(^{1}H) / ppm
O1	O2	Al1	3.3184	2.814	48.32	44.22	7.06
	O2	Al2	3.3184	2.866	48.32	41.52	7.17
O2	O1	Al1	3.3184	2.814	78.96	59.76	7.06
	-	Al4					3.95
O3	O6	Al2	3.4972	3.185	34.06	35.22	5.81
	O3	Al2	3.7047	3.301	12.74	12.96	
	O6	Al4	3.4972	3.421	34.06	40.06	6.00
	O3	Al4	3.7047	3.290	12.74	6.23	
O4	-	Al1					4.40
O5	O5	Al3	3.5581	3.153	37.29	35.33	6.37
	O9	Al3	3.8643	3.507	37.35	37.32	
	O5	Al4	3.5581	3.082	37.29	31.06	6.18
O6	O4	Al2	3.7172	2.897	76.45	40.97	7.39
	O5	Al2	3.8940	3.969*	42.73	74.03	
	O5	Al3	3.8940	3.971*	42.73	57.40	3.78
O7	O9	Al1	3.6537	3.302	12.47	25.83	5.91
O8	-	Al3					4.84
	-	Al4					4.21
O9	O5	Al2	3.8643	3.557	27.95	38.80	5.07
	O5	Al2	3.8643	3.518	27.95	37.28	
O10	-	Al3					5.17

* no hydrogen bond formed in this pair of oxygen atoms

Table S2: Results of the alignment angle analysis on the crystal structure of calcined SSZ-42. [2a, 12]

O (Donor)	O (acceptor)	distance / Å	φ_1 / °	φ_2 / °	φ_3 / °	κ / °
O1	O2	3.3184	123.384	79.844	147.344	48.32
	O1	3.8071	117.013	81.596	147.344	56.78
	O1	3.8265	76.962	76.456	147.344	144.86
O2	O1	3.3182	99.464	86.43	149.047	78.96
	O9	3.9000	96.205	68.105	149.047	119.75
	O6	3.9351	91.576	68.232	149.047	130.04
O3	O6	3.4972	141.447	75.044	143.132	34.06
	O3	3.7047	117.93	98.538	143.132	12.74
O4	O4	3.3519	85.124	85.124	170.274	180.00
	O6	3.7173	100.255	80.18	170.274	87.47
O5	O5	3.5581	126.837	80.054	148.881	37.29
	O5	3.5933	81.467	75.01	148.881	139.35
	O7	3.7200	113.631	74.819	148.881	74.99
	O9	3.8643	141.397	69.207	148.881	37.35
	O6	3.8934	107.902	62.1	148.881	107.41
O6	O3	3.4979	106.752	84.761	156.188	61.49
	O6	3.6792	79.726	77.552	156.188	162.68
	O4	3.7172	110.613	75.205	156.188	76.45
	O5	3.8940	136.358	65.128	156.188	42.73
	O2	3.9346	114.525	76.503	156.188	63.87
O7	O9	3.5615	81.417	81.417	148.352	123.18
	O9	3.6537	105.441	105.441	148.352	12.47
	O5	3.7201	128.646	72.8	148.352	52.92
	O7	3.8028	76.866	76.866	148.352	146.44
O8	O8	3.8232	78.277	71.439	139.005	138.13
O9	O7	3.5616	80.114	80.114	148.867	129.78
	O10	3.5771	82.367	82.367	148.867	119.67
	O7	3.6525	97.597	97.597	148.867	60.49
	O5	3.8643	132.889	78.085	148.867	27.95
	O2	3.8989	138.2	60.876	148.867	61.17
O10	O9	3.5771	80.045	80.045	140.291	120.60

References

[1] S. Elomari, S. Zones, *US 6,821,502* **2002**.
[2] a) C. Schroeder, V. Siozios, C. Mück-Lichtenfeld, M. Hunger, M. R. Hansen, H. Koller, *Chem. Mater.* **2020**, *32*, 1564-1574; b) C. Schroeder, V. Siozios, M. Hunger, M. R. Hansen, H. Koller, *J. Phys. Chem. C* **2020**, *124*, 23380-23386.
[3] D. Massiot, F. Fayon, M. Capron, I. King, S. Le Calve, B. Alonso, J. O. Durand, B. Bujoli, Z. H. Gan, G. Hoatson, *Magn. Reson. Chem.* **2002**, *40*, 70-76.
[4] M. Bak, J. T. Rasmussen, N. C. Nielsen, *J. Magn. Reson.* **2000**, *147*, 296-330.
[5] a) C. Schroeder, M. R. Hansen, H. Koller, *Angew. Chem. Int. Ed.* **2018**, *57*, 14281-14285; b) H. Koller, T. Uesbeck, M. R. Hansen, M. Hunger, *J. Phys. Chem. C* **2017**, *121*, 25930-25940.
[6] J. P. Perdew, K. Burke, M. Ernzerhof, *Phys. Rev. Lett.* **1996**, *77*, 3865-3868.
[7] S. Grimme, J. Antony, S. Ehrlich, H. Krieg, *J Chem Phys* **2010**, *132*, 154104.
[8] F. Weigend, R. Ahlrichs, *Phys. Chem. Chem. Phys.* **2005**, *7*, 3297-3305.
[9] Y. Zhao, D. G. Truhlar, *J. Phys. Chem. A* **2005**, *109*, 5656-5667.
[10] S. Sklenak, J. Dědeček, C. Li, B. Wichterlová, V. Gábová, M. Sierka, J. Sauer, *Angew. Chem. Int. Ed.* **2007**, *46*, 7286-7289.
[11] TURBOMOLE V7.4, a development of University of Karlsruhe and Forschungszentrum Karlsruhe GmbH, 1989-2007, TURBOMOLE GmbH. http://www.turbomole.com (last accessed December 2019).
[12] C. Y. Chen, L. W. Finger, R. C. Medrud, C. L. Kibby, P. A. Crozier, I. Y. Chan, T. V. Harris, L. W. Beck, S. I. Zones, *Chem. Eur. J.* **1998**, *4*, 1312-1323.
[13] U. Eichler, M. Brändle, J. Sauer, *J. Phys. Chem. B* **1997**, *101*, 10035-10050.
[14] J. Sauer, *Faraday Discuss.* **2016**, *188*, 227-234.
[15] G. Noh, S. I. Zones, E. Iglesia, *J. Phys. Chem. C* **2018**, *122*, 25475-25497.

E Cartesian Atomic Coordinates (in Å) for the Zeolite Clusters

In the following, all clusters are given with their atomic coordinates. For each model, the first line begins with the total number of atoms and the position of the Al atom in the center of the cluster (e.g. Al1) and the oxygen atom that is protonated (e.g. O1).

	194 atoms	Al1-O1	
Energy =		-22737.88748043	Hartree
H	18.1919	9.1939	7.2970
Al	17.3210	8.2693	5.2263
O	18.6224	8.8265	6.4845
O	16.1219	9.4225	5.6165
O	18.1167	8.4292	3.7300
O	16.9285	6.6455	5.6640
O	20.1437	8.6229	-1.2776
O	17.7027	9.3273	-2.0212
O	18.3874	6.7528	-2.1162
O	14.6283	9.2724	13.0378
O	16.5157	8.7190	11.2369
O	15.5085	6.7628	12.8082
O	17.3207	8.5893	1.1614
O	19.7345	9.2921	1.8336
O	19.0674	6.7172	1.9293
O	15.7896	8.5391	8.6581
O	18.2219	9.2307	9.2407
O	17.4756	6.7329	9.7310
O	17.2996	4.8677	1.1541
O	19.6841	4.1459	1.9939
O	17.9113	5.0836	3.7264
O	15.7915	4.9451	8.6740
O	18.2149	4.2028	9.3908
O	16.4476	4.7766	11.2491
O	20.1160	4.8685	-1.2766
O	17.6851	4.1651	-2.0504
O	18.6208	4.6669	6.2534
O	16.1962	4.0790	5.4810
O	22.0347	9.2486	0.5251
O	21.7912	6.7402	-0.3214
O	20.6122	9.1423	8.1495
O	21.1012	8.6596	5.5500
O	20.1394	6.7079	7.1224
O	15.4350	9.2279	-0.5896
O	14.7945	8.7018	1.9477
O	15.5791	6.7479	0.2983
O	13.8419	9.2630	6.9757
O	13.2889	8.6677	9.5024
O	14.0104	6.7398	7.7974
O	15.4050	4.2628	-0.5898
O	14.7617	4.7965	1.9288
O	13.9392	4.2277	6.9384
O	13.2527	4.7841	9.4526
O	23.3162	11.0826	6.9339
O	22.5697	11.6492	9.4380
O	23.5692	13.5628	7.8375
O	21.9398	4.2248	0.5413
O	20.5255	4.2682	8.0656

O	21.1654	4.8011	5.5565
O	12.5184	11.0941	0.5349
O	13.2376	11.6916	-1.9968
O	11.0084	11.0984	8.0510
O	11.6714	11.6795	5.5230
O	10.7727	13.5867	7.1858
O	13.8764	8.5139	4.4356
O	12.2377	9.2702	2.4631
O	13.0532	6.7668	2.6134
O	12.3093	8.4447	11.9421
O	10.7118	9.2664	9.9597
O	11.4816	6.7468	10.0684
O	23.3084	1.6166	4.4200
O	21.5986	2.4352	2.4871
O	23.2509	15.3689	4.3528
O	22.2856	13.5757	2.6217
O	21.9848	8.4751	3.0613
O	23.6448	9.2557	5.0276
O	22.8702	6.7511	4.8819
O	20.3648	8.4732	10.6953
O	21.4241	6.7449	12.4172
O	12.5301	1.6365	3.0762
O	14.2466	2.4113	5.0212
O	11.7598	15.5720	5.5949
O	13.4712	13.5993	4.9105
O	21.9976	5.0312	3.0542
O	23.6955	4.2483	4.9863
O	20.4712	4.9610	10.6332
O	12.6010	11.8846	3.0599
O	14.2084	11.1012	5.0646
O	13.9025	5.0144	4.4208
O	12.2086	4.2724	2.4804
O	23.2859	11.8435	4.3789
O	21.6019	11.0478	2.4428
O	20.0381	11.0345	9.9715
O	20.7782	13.5669	10.1227
O	15.8194	11.1146	-2.4432
O	12.7276	11.0514	12.5437
O	11.0269	11.8698	10.6015
O	23.3248	16.0641	6.9458
O	22.5482	15.4937	9.4197
Si	18.9259	8.2963	-2.3161
Si	15.9321	8.3465	12.7255
Si	18.5347	8.2526	2.1995
Si	16.9648	8.2807	9.7423
Si	18.4828	5.2176	2.2181
Si	16.9693	5.1710	9.7620
Si	18.9090	5.2058	-2.3233
Si	17.3978	5.1586	5.2713
Si	15.8775	5.1549	12.7370

Si	21.6653	8.3095	-0.7637
Si	20.2016	8.2899	6.8286
Si	15.7857	8.3142	0.7135
Si	14.2248	8.2986	8.2193
Si	15.7662	5.1805	0.7064
Si	14.2425	5.1810	8.2182
Si	23.6503	11.9850	8.2571
Si	21.6377	5.1716	-0.7572
Si	20.0985	5.1078	6.7360
Si	12.1851	12.0182	-0.7864
Si	10.6512	12.0171	6.7429
Si	13.4994	8.3145	2.8653
Si	11.9491	8.2813	10.3540
Si	22.8706	1.4478	2.8370
Si	22.8207	15.1221	2.7859
Si	22.4050	8.2843	4.6143
Si	20.8674	8.2814	12.2538
Si	12.9658	1.4434	4.6545
Si	13.0280	15.1595	4.6283
Si	22.4308	5.2027	4.6122
Si	20.8574	5.2074	12.2112
Si	12.9847	12.0701	4.6379
Si	13.4848	5.2159	2.8574
Si	11.9411	5.1884	10.3654
Si	22.8373	12.0507	2.8126
Si	21.2852	12.0334	10.3712
Si	16.0811	9.5425	-2.0654
Si	14.5323	9.5678	5.5110
Si	13.0065	9.4974	12.9905
Si	23.9588	2.6920	5.4879
Si	21.3279	9.5121	1.9738
Si	19.8237	9.4780	9.5515
Si	11.8949	2.7196	2.0052
Si	21.2978	3.9670	2.0221
Si	19.8263	3.9540	9.5148
Si	11.9043	10.8138	2.0259
Si	10.3705	10.8134	9.5307
Si	16.0489	3.9685	-2.0788
Si	14.5787	3.9443	5.4557
Si	23.9613	10.8104	5.4491
Si	23.9529	16.3629	5.4504
Si	23.6445	15.1441	8.2608
Si	21.2678	15.1260	10.3815
Si	14.5579	12.0707	-2.8971
Si	10.6769	15.1737	6.7670
Si	11.4756	12.0661	12.1735
H	19.4046	8.4935	-3.7027
H	17.0009	8.6791	13.6903
H	19.4014	5.0249	-3.7050
H	16.9563	4.8308	13.7186

H	14.6395	4.3474	12.9640
H	22.6387	8.6282	-1.8200
H	24.9920	11.6378	8.7714
H	22.6210	4.8697	-1.8199
H	10.8328	11.6530	-1.2807
H	12.2972	13.4547	-0.4076
H	9.3014	11.6866	6.2548
H	24.0091	1.8385	1.9552
H	22.4173	0.0442	2.6260
H	24.0267	15.3065	1.9252
H	21.7065	16.0418	2.4095
H	19.6705	8.5048	13.1110
H	21.9803	9.2356	12.5568
H	11.8345	1.8331	5.5451
H	13.4085	0.0336	4.8485
H	12.5996	15.2657	3.2039
H	14.1958	16.0393	4.9496
H	19.6289	5.0357	13.0458
H	21.9531	4.2768	12.5922
H	12.3373	5.0708	11.8014
H	10.7986	4.2946	10.0039
H	23.9959	11.7716	1.9299
H	21.6733	11.8402	11.7943
H	15.4716	8.6610	-3.0978
H	12.4333	9.2495	14.3288
H	25.4391	2.5263	5.5241
H	23.3166	2.4662	6.8221
H	10.4096	2.5631	1.9843
H	12.5192	2.4704	0.6705
H	20.0743	2.5439	9.8811
H	10.4291	10.9967	2.0308
H	8.8949	10.9814	9.5023
H	15.4772	4.9250	-3.0743
H	15.7510	2.5467	-2.4085
H	25.4314	11.0050	5.4734
H	25.4238	16.0941	5.4801
H	23.6379	17.7724	5.0856
H	25.0022	15.4302	8.7729
H	21.7133	15.2636	11.8029
H	20.1238	16.0234	10.0492
H	14.2092	11.7873	-4.3215
H	14.9533	13.4990	-2.6855
H	9.3067	15.4305	6.2392
H	10.9743	15.9519	8.0020
H	10.3052	11.7358	13.0469
H	11.9492	13.4722	12.3581

	194 atoms	Al1-O2		
Energy =		-22737.88526133	Hartree	
H	16.2759	9.4182	6.8642
Al	17.4322	8.2537	5.1767
O	18.5994	8.5650	6.4002
O	16.0981	9.3735	5.8881
O	17.7298	8.8552	3.6011
O	16.7813	6.6542	5.2253
O	20.1436	8.6338	-1.2808
O	17.7078	9.3327	-2.0400
O	18.3946	6.7510	-2.1103
O	14.6296	9.3111	13.0630
O	16.3369	8.5088	11.2070
O	15.4213	6.7667	13.0284
O	17.3665	8.4531	1.0067
O	19.7101	9.2682	1.8760
O	18.9454	6.7797	2.3824
O	15.7673	8.6942	8.5991
O	18.1559	9.3237	9.4430
O	17.4117	6.7571	9.5054
O	17.3275	5.0323	1.1566
O	19.7222	4.2661	1.9105
O	17.9048	4.7133	3.7462
O	15.7673	4.8254	8.6868
O	18.1903	4.1970	9.4777
O	16.4101	4.9980	11.2560
O	20.1233	4.8575	-1.2757
O	17.6951	4.1604	-2.0634
O	18.5582	4.9561	6.3180
O	16.1709	4.0957	5.6195
O	22.0036	9.2392	0.5452
O	21.7715	6.7416	-0.3220
O	20.4957	9.2159	8.1267
O	21.1259	8.7188	5.5917
O	20.3811	6.7347	7.2127
O	15.4302	9.2471	-0.6193
O	14.8447	8.7134	1.9062
O	15.4700	6.7403	0.2347
O	13.7461	9.2136	6.9859
O	13.2710	8.6633	9.5338
O	14.0893	6.7311	7.8533
O	15.4530	4.2352	-0.5707
O	14.7983	4.7936	1.9361
O	13.8590	4.2448	6.9441
O	13.2325	4.7908	9.4670
O	23.3208	11.0844	6.9316
O	22.5716	11.6468	9.4415
O	23.5629	13.5636	7.8458
O	21.9846	4.2290	0.5341
O	20.4461	4.2366	8.0404

O	21.0888	4.7882	5.5241
O	12.5155	11.0946	0.5445
O	13.2483	11.6870	-1.9866
O	11.0068	11.0869	8.0568
O	11.7001	11.6673	5.5513
O	10.7706	13.5814	7.1895
O	14.0350	8.5015	4.4011
O	12.2964	9.2807	2.5070
O	13.1023	6.7705	2.6271
O	12.3085	8.4385	11.9675
O	10.7048	9.2733	9.9843
O	11.4588	6.7499	10.0852
O	23.2968	1.6373	4.4115
O	21.5815	2.4482	2.4691
O	23.2495	15.3698	4.3512
O	22.2850	13.5708	2.6206
O	21.9755	8.4739	3.0888
O	23.6691	9.2463	5.0183
O	22.8354	6.7520	4.9127
O	20.3830	8.5044	10.6741
O	21.4134	6.7520	12.4023
O	12.5410	1.6563	3.0828
O	14.2502	2.4057	5.0499
O	11.7624	15.5496	5.5952
O	13.4961	13.5947	4.9141
O	22.0235	5.0314	3.0602
O	23.6635	4.2603	5.0284
O	20.4814	4.9566	10.6243
O	12.6131	11.8767	3.0788
O	14.2261	11.0832	5.0928
O	13.9126	4.9956	4.4235
O	12.2277	4.2860	2.4632
O	23.2823	11.8334	4.3772
O	21.5882	11.0448	2.4417
O	20.0229	11.0702	9.9943
O	20.7950	13.5772	10.1165
O	15.8321	11.1259	-2.4640
O	12.7201	11.0517	12.5318
O	11.0213	11.8841	10.5991
O	23.3252	16.0633	6.9450
O	22.5535	15.5152	9.4215
Si	18.9313	8.2950	-2.3169
Si	15.8913	8.3174	12.7698
Si	18.4277	8.3395	2.2498
Si	16.9347	8.3018	9.7132
Si	18.4688	5.2171	2.3105
Si	16.9495	5.1993	9.7384
Si	18.9199	5.2040	-2.3215
Si	17.3556	5.1491	5.2236
Si	15.8548	5.1938	12.7812

Si	21.6619	8.3145	-0.7569
Si	20.1263	8.3044	6.8160
Si	15.7937	8.2879	0.6428
Si	14.2010	8.2918	8.2647
Si	15.7768	5.2035	0.6969
Si	14.2381	5.1441	8.2402
Si	23.6477	11.9810	8.2584
Si	21.6454	5.1705	-0.7570
Si	20.1048	5.1950	6.7604
Si	12.1879	12.0168	-0.7863
Si	10.6514	12.0117	6.7541
Si	13.5717	8.3109	2.8310
Si	11.9279	8.2774	10.3901
Si	22.8573	1.4597	2.8305
Si	22.8198	15.1167	2.7855
Si	22.3894	8.2969	4.6561
Si	20.8627	8.2942	12.2313
Si	12.9698	1.4450	4.6610
Si	13.0336	15.1570	4.6279
Si	22.3966	5.2093	4.6373
Si	20.8588	5.2122	12.2044
Si	12.9942	12.0742	4.6505
Si	13.5171	5.2053	2.8582
Si	11.9259	5.1905	10.3780
Si	22.8319	12.0408	2.8142
Si	21.2871	12.0378	10.3730
Si	16.0916	9.5537	-2.0896
Si	14.4390	9.5493	5.5532
Si	13.0095	9.5074	13.0026
Si	23.9527	2.7006	5.4915
Si	21.3093	9.4984	2.0050
Si	19.7795	9.5190	9.5523
Si	11.8992	2.7277	2.0049
Si	21.3212	3.9942	2.0115
Si	19.8111	3.9516	9.5192
Si	11.9143	10.8086	2.0357
Si	10.3671	10.8167	9.5422
Si	16.0612	3.9649	-2.0804
Si	14.5632	3.9406	5.4916
Si	23.9636	10.8001	5.4489
Si	23.9523	16.3614	5.4506
Si	23.6439	15.1442	8.2629
Si	21.2744	15.1326	10.3800
Si	14.5609	12.0732	-2.9005
Si	10.6752	15.1703	6.7700
Si	11.4722	12.0708	12.1743
H	19.4046	8.4935	-3.7027
H	17.0009	8.6791	13.6903
H	19.4014	5.0249	-3.7050
H	16.9563	4.8308	13.7186

H	14.6395	4.3474	12.9640
H	22.6387	8.6282	-1.8200
H	24.9920	11.6378	8.7714
H	22.6210	4.8697	-1.8199
H	10.8328	11.6530	-1.2807
H	12.2972	13.4547	-0.4076
H	9.3014	11.6866	6.2548
H	24.0091	1.8385	1.9552
H	22.4173	0.0442	2.6260
H	24.0267	15.3065	1.9252
H	21.7065	16.0418	2.4095
H	19.6705	8.5048	13.1110
H	21.9803	9.2356	12.5568
H	11.8345	1.8331	5.5451
H	13.4085	0.0336	4.8485
H	12.5996	15.2657	3.2039
H	14.1958	16.0393	4.9496
H	19.6289	5.0357	13.0458
H	21.9531	4.2768	12.5922
H	12.3373	5.0708	11.8014
H	10.7986	4.2946	10.0039
H	23.9959	11.7716	1.9299
H	21.6733	11.8402	11.7943
H	15.4716	8.6610	-3.0978
H	12.4333	9.2495	14.3288
H	25.4391	2.5263	5.5241
H	23.3166	2.4662	6.8221
H	10.4096	2.5631	1.9843
H	12.5192	2.4704	0.6705
H	20.0743	2.5439	9.8811
H	10.4291	10.9967	2.0308
H	8.8949	10.9814	9.5023
H	15.4772	4.9250	-3.0743
H	15.7510	2.5467	-2.4085
H	25.4314	11.0050	5.4734
H	25.4238	16.0941	5.4801
H	23.6379	17.7724	5.0856
H	25.0022	15.4302	8.7729
H	21.7133	15.2636	11.8029
H	20.1238	16.0234	10.0492
H	14.2092	11.7873	-4.3215
H	14.9533	13.4990	-2.6855
H	9.3067	15.4305	6.2392
H	10.9743	15.9519	8.0020
H	10.3052	11.7358	13.0469
H	11.9492	13.4722	12.3581

	194 atoms	Al1-O4	
Energy =		-22737.88259072	Hartree
H	17.4226	9.7679	3.5902
Al	17.3123	8.2237	5.3760
O	18.6417	8.4462	6.4091
O	16.0919	9.4046	5.5749
O	17.8509	8.8923	3.6671
O	16.8243	6.6071	5.0573
O	20.1423	8.6576	-1.2727
O	17.6988	9.3157	-2.0172
O	18.4238	6.7469	-2.0882
O	14.6311	9.3063	13.0653
O	16.3853	8.5241	11.2302
O	15.4274	6.7633	13.0123
O	17.3412	8.4835	1.0955
O	19.7471	9.2255	1.8701
O	18.8932	6.7510	2.5138
O	15.7802	8.5453	8.6405
O	18.1675	9.3137	9.4360
O	17.5181	6.7419	9.5954
O	17.3339	5.0412	1.1801
O	19.7504	4.2953	1.8697
O	17.9585	4.5781	3.7622
O	15.7790	4.9316	8.6735
O	18.1756	4.1682	9.4624
O	16.4035	4.9564	11.2542
O	20.1284	4.8268	-1.2747
O	17.6957	4.1611	-2.0635
O	18.5753	5.0017	6.3225
O	16.1872	4.0816	5.6316
O	22.0268	9.2259	0.5421
O	21.7422	6.7379	-0.3280
O	20.5161	9.2071	8.1273
O	21.1401	8.7124	5.5920
O	20.4818	6.7074	7.2297
O	15.4406	9.2437	-0.5836
O	14.8502	8.7068	1.9802
O	15.4912	6.7422	0.2674
O	13.8189	9.2572	6.9795
O	13.2718	8.6592	9.4993
O	14.0132	6.7381	7.7859
O	15.4674	4.2453	-0.5649
O	14.8049	4.7740	1.9493
O	13.8852	4.2216	6.9625
O	13.2450	4.8022	9.4697
O	23.3207	11.0821	6.9350
O	22.5764	11.6512	9.4458
O	23.5662	13.5632	7.8395
O	22.0140	4.2315	0.5323
O	20.4426	4.2085	8.0385

O	21.0945	4.7651	5.5170
O	12.5227	11.0996	0.5292
O	13.2389	11.6908	-1.9982
O	11.0068	11.0952	8.0534
O	11.6684	11.6768	5.5232
O	10.7746	13.5852	7.1841
O	13.8214	8.5366	4.4362
O	12.2566	9.2455	2.4009
O	13.1104	6.7532	2.6020
O	12.3204	8.4452	11.9489
O	10.7076	9.2735	9.9770
O	11.4575	6.7517	10.0847
O	23.2978	1.6295	4.4136
O	21.5873	2.4547	2.4727
O	23.2510	15.3680	4.3521
O	22.2838	13.5687	2.6237
O	22.0020	8.4625	3.0956
O	23.6874	9.2391	5.0237
O	22.8484	6.7405	4.9205
O	20.4016	8.5094	10.6709
O	21.4134	6.7497	12.3976
O	12.5403	1.6418	3.0809
O	14.2548	2.4052	5.0416
O	11.7593	15.5700	5.5950
O	13.4680	13.5972	4.8961
O	22.0290	5.0434	3.0584
O	23.6721	4.2508	5.0164
O	20.4682	4.9528	10.6216
O	12.6101	11.8530	3.0624
O	14.2132	11.1077	5.0727
O	13.9341	4.9960	4.4348
O	12.2376	4.2712	2.4811
O	23.2751	11.8188	4.3787
O	21.5897	11.0377	2.4379
O	20.0286	11.0688	9.9816
O	20.7966	13.5765	10.1152
O	15.8214	11.1172	-2.4441
O	12.7218	11.0539	12.5388
O	11.0214	11.8824	10.5993
O	23.3249	16.0628	6.9454
O	22.5533	15.5126	9.4215
Si	18.9336	8.2966	-2.3130
Si	15.8974	8.3187	12.7706
Si	18.4900	8.2830	2.2109
Si	16.9590	8.2761	9.7307
Si	18.4667	5.1561	2.3430
Si	16.9646	5.2083	9.7519
Si	18.9279	5.1940	-2.3183
Si	17.3830	5.1040	5.2090
Si	15.8551	5.1888	12.7776

Si	21.6611	8.3109	-0.7639
Si	20.1681	8.2672	6.8368
Si	15.7569	8.2894	0.6922
Si	14.2217	8.2968	8.2281
Si	15.7761	5.1952	0.7180
Si	14.2310	5.1823	8.2326
Si	23.6476	11.9829	8.2630
Si	21.6466	5.1605	-0.7591
Si	20.1310	5.1855	6.7704
Si	12.1836	12.0215	-0.7899
Si	10.6497	12.0141	6.7482
Si	13.5121	8.3093	2.8623
Si	11.9428	8.2820	10.3670
Si	22.8606	1.4569	2.8332
Si	22.8207	15.1189	2.7881
Si	22.4097	8.2866	4.6732
Si	20.8631	8.2938	12.2307
Si	12.9711	1.4442	4.6619
Si	13.0261	15.1610	4.6282
Si	22.4033	5.1999	4.6408
Si	20.8553	5.2112	12.2010
Si	12.9814	12.0663	4.6418
Si	13.5203	5.1988	2.8745
Si	11.9292	5.1961	10.3756
Si	22.8355	12.0429	2.8189
Si	21.2844	12.0357	10.3736
Si	16.0809	9.5468	-2.0707
Si	14.5017	9.5658	5.5223
Si	13.0108	9.5048	12.9976
Si	23.9550	2.6938	5.4928
Si	21.3606	9.4859	2.0080
Si	19.7804	9.5140	9.5492
Si	11.9004	2.7199	2.0093
Si	21.3458	3.9988	2.0078
Si	19.7970	3.9433	9.5212
Si	11.9063	10.8015	2.0163
Si	10.3696	10.8161	9.5374
Si	16.0613	3.9656	-2.0791
Si	14.5727	3.9338	5.5063
Si	23.9666	10.7965	5.4580
Si	23.9528	16.3623	5.4535
Si	23.6432	15.1429	8.2646
Si	21.2724	15.1309	10.3812
Si	14.5561	12.0733	-2.8991
Si	10.6760	15.1725	6.7687
Si	11.4734	12.0690	12.1733
H	19.4046	8.4935	-3.7027
H	17.0009	8.6791	13.6903
H	19.4014	5.0249	-3.7050
H	16.9563	4.8308	13.7186

H	14.6395	4.3474	12.9640
H	22.6387	8.6282	-1.8200
H	24.9920	11.6378	8.7714
H	22.6210	4.8697	-1.8199
H	10.8328	11.6530	-1.2807
H	12.2972	13.4547	-0.4076
H	9.3014	11.6866	6.2548
H	24.0091	1.8385	1.9552
H	22.4173	0.0442	2.6260
H	24.0267	15.3065	1.9252
H	21.7065	16.0418	2.4095
H	19.6705	8.5048	13.1110
H	21.9803	9.2356	12.5568
H	11.8345	1.8331	5.5451
H	13.4085	0.0336	4.8485
H	12.5996	15.2657	3.2039
H	14.1958	16.0393	4.9496
H	19.6289	5.0357	13.0458
H	21.9531	4.2768	12.5922
H	12.3373	5.0708	11.8014
H	10.7986	4.2946	10.0039
H	23.9959	11.7716	1.9299
H	21.6733	11.8402	11.7943
H	15.4716	8.6610	-3.0978
H	12.4333	9.2495	14.3288
H	25.4391	2.5263	5.5241
H	23.3166	2.4662	6.8221
H	10.4096	2.5631	1.9843
H	12.5192	2.4704	0.6705
H	20.0743	2.5439	9.8811
H	10.4291	10.9967	2.0308
H	8.8949	10.9814	9.5023
H	15.4772	4.9250	-3.0743
H	15.7510	2.5467	-2.4085
H	25.4314	11.0050	5.4734
H	25.4238	16.0941	5.4801
H	23.6379	17.7724	5.0856
H	25.0022	15.4302	8.7729
H	21.7133	15.2636	11.8029
H	20.1238	16.0234	10.0492
H	14.2092	11.7873	-4.3215
H	14.9533	13.4990	-2.6855
H	9.3067	15.4305	6.2392
H	10.9743	15.9519	8.0020
H	10.3052	11.7358	13.0469
H	11.9492	13.4722	12.3581

	194 atoms	Al1-O7		
Energy =		-22737.88335403	Hartree	
H	15.6851	6.6863	5.7249
Al	17.4161	8.4288	5.2836
O	18.6433	8.4151	6.4634
O	16.1006	9.4405	5.6585
O	17.9303	8.3978	3.6616
O	16.6318	6.6879	5.4773
O	20.1397	8.6109	-1.2749
O	17.7003	9.3296	-2.0230
O	18.3748	6.7538	-2.1238
O	14.6352	9.3148	13.0714
O	16.3840	8.5162	11.2340
O	15.4204	6.7674	13.0238
O	17.3445	8.5681	1.0717
O	19.7135	9.3088	1.8964
O	19.0774	6.7298	1.9458
O	15.7628	8.5886	8.6580
O	18.1671	9.3201	9.4435
O	17.4911	6.7497	9.5719
O	17.2974	4.8818	1.1725
O	19.6745	4.1434	2.0249
O	17.9028	5.1180	3.7396
O	15.7775	4.9032	8.6796
O	18.1715	4.1742	9.4727
O	16.3948	4.9715	11.2574
O	20.1112	4.8791	-1.2751
O	17.6870	4.1647	-2.0504
O	18.5784	5.0729	6.3209
O	16.2106	4.0823	5.5451
O	22.0021	9.2359	0.5464
O	21.7978	6.7380	-0.3216
O	20.5276	9.2243	8.1427
O	21.1467	8.7151	5.6088
O	20.5101	6.7198	7.2655
O	15.4144	9.2241	-0.6051
O	14.8211	8.6925	1.9446
O	15.5592	6.7402	0.2875
O	13.8020	9.2780	6.9620
O	13.2405	8.5410	9.4480
O	14.1437	6.7424	7.6473
O	15.4150	4.2503	-0.5858
O	14.7686	4.7866	1.9297
O	13.9324	4.2068	6.9423
O	13.2127	4.9128	9.4133
O	23.3226	11.0788	6.9339
O	22.5729	11.6474	9.4431
O	23.5632	13.5627	7.8436
O	21.9175	4.2198	0.5420
O	20.4155	4.2030	8.0320

O	21.0854	4.7769	5.5217
O	12.5172	11.0882	0.5310
O	13.2380	11.6913	-1.9974
O	11.0091	11.1052	8.0514
O	11.6663	11.6730	5.5176
O	10.7796	13.5898	7.1748
O	13.9144	8.4923	4.4432
O	12.2697	9.2843	2.4810
O	13.0722	6.7658	2.6305
O	12.3467	8.4514	11.9323
O	10.7169	9.2928	9.9775
O	11.3671	6.7547	10.1367
O	23.3098	1.6172	4.4191
O	21.5921	2.4283	2.4861
O	23.2510	15.3712	4.3524
O	22.2888	13.5742	2.6243
O	21.9732	8.4755	3.0922
O	23.6803	9.2432	5.0132
O	22.8344	6.7446	4.9016
O	20.3937	8.5063	10.6752
O	21.4138	6.7502	12.3989
O	12.5247	1.6201	3.0717
O	14.2551	2.4011	5.0005
O	11.7555	15.5872	5.5917
O	13.4576	13.6063	4.9172
O	21.9939	5.0271	3.0561
O	23.6549	4.2478	5.0093
O	20.4699	4.9536	10.6208
O	12.5804	11.9134	3.0479
O	14.1944	11.1055	5.0225
O	13.9740	5.0306	4.4322
O	12.2310	4.2569	2.5293
O	23.2864	11.8338	4.3815
O	21.5998	11.0514	2.4436
O	20.0238	11.0742	9.9996
O	20.7988	13.5790	10.1150
O	15.8205	11.1182	-2.4459
O	12.7195	11.0558	12.5360
O	11.0190	11.8964	10.5982
O	23.3259	16.0635	6.9460
O	22.5553	15.5164	9.4225
Si	18.9233	8.2967	-2.3165
Si	15.8964	8.3215	12.7751
Si	18.5042	8.2629	2.1830
Si	16.9524	8.2917	9.7285
Si	18.4941	5.2354	2.2149
Si	16.9608	5.2148	9.7583
Si	18.9078	5.2107	-2.3260
Si	17.3776	5.1642	5.2533
Si	15.8513	5.1957	12.7852

Si	21.6642	8.3100	-0.7558
Si	20.1839	8.2801	6.8549
Si	15.7898	8.3098	0.6884
Si	14.2322	8.3025	8.1866
Si	15.7669	5.1803	0.7010
Si	14.2512	5.1993	8.2014
Si	23.6469	11.9809	8.2601
Si	21.6366	5.1726	-0.7569
Si	20.1541	5.2143	6.7834
Si	12.1852	12.0185	-0.7883
Si	10.6509	12.0197	6.7409
Si	13.5279	8.3194	2.8686
Si	11.9215	8.2716	10.3659
Si	22.8713	1.4472	2.8384
Si	22.8213	15.1211	2.7873
Si	22.3976	8.2973	4.6567
Si	20.8629	8.2953	12.2322
Si	12.9588	1.4340	4.6477
Si	13.0239	15.1689	4.6290
Si	22.3926	5.2049	4.6270
Si	20.8570	5.2130	12.2027
Si	12.9680	12.0846	4.6265
Si	13.5059	5.2168	2.8670
Si	11.9078	5.2169	10.3775
Si	22.8402	12.0482	2.8192
Si	21.2873	12.0398	10.3746
Si	16.0799	9.5461	-2.0725
Si	14.5219	9.5931	5.5128
Si	13.0148	9.5101	12.9972
Si	23.9545	2.6948	5.4912
Si	21.3185	9.5091	2.0083
Si	19.7809	9.5233	9.5588
Si	11.8971	2.7185	2.0095
Si	21.2963	3.9634	2.0308
Si	19.7932	3.9443	9.5267
Si	11.9063	10.8198	2.0243
Si	10.3703	10.8322	9.5336
Si	16.0530	3.9667	-2.0821
Si	14.5772	3.9164	5.4696
Si	23.9663	10.7990	5.4521
Si	23.9529	16.3627	5.4526
Si	23.6437	15.1435	8.2637
Si	21.2746	15.1339	10.3801
Si	14.5575	12.0726	-2.8985
Si	10.6773	15.1791	6.7649
Si	11.4712	12.0731	12.1730
H	19.4046	8.4935	-3.7027
H	17.0009	8.6791	13.6903
H	19.4014	5.0249	-3.7050
H	16.9563	4.8308	13.7186

H	14.6395	4.3474	12.9640
H	22.6387	8.6282	-1.8200
H	24.9920	11.6378	8.7714
H	22.6210	4.8697	-1.8199
H	10.8328	11.6530	-1.2807
H	12.2972	13.4547	-0.4076
H	9.3014	11.6866	6.2548
H	24.0091	1.8385	1.9552
H	22.4173	0.0442	2.6260
H	24.0267	15.3065	1.9252
H	21.7065	16.0418	2.4095
H	19.6705	8.5048	13.1110
H	21.9803	9.2356	12.5568
H	11.8345	1.8331	5.5451
H	13.4085	0.0336	4.8485
H	12.5996	15.2657	3.2039
H	14.1958	16.0393	4.9496
H	19.6289	5.0357	13.0458
H	21.9531	4.2768	12.5922
H	12.3373	5.0708	11.8014
H	10.7986	4.2946	10.0039
H	23.9959	11.7716	1.9299
H	21.6733	11.8402	11.7943
H	15.4716	8.6610	-3.0978
H	12.4333	9.2495	14.3288
H	25.4391	2.5263	5.5241
H	23.3166	2.4662	6.8221
H	10.4096	2.5631	1.9843
H	12.5192	2.4704	0.6705
H	20.0743	2.5439	9.8811
H	10.4291	10.9967	2.0308
H	8.8949	10.9814	9.5023
H	15.4772	4.9250	-3.0743
H	15.7510	2.5467	-2.4085
H	25.4314	11.0050	5.4734
H	25.4238	16.0941	5.4801
H	23.6379	17.7724	5.0856
H	25.0022	15.4302	8.7729
H	21.7133	15.2636	11.8029
H	20.1238	16.0234	10.0492
H	14.2092	11.7873	-4.3215
H	14.9533	13.4990	-2.6855
H	9.3067	15.4305	6.2392
H	10.9743	15.9519	8.0020
H	10.3052	11.7358	13.0469
H	11.9492	13.4722	12.3581

	207 atoms	Al2-O1	
Energy =	-25085.92363058	Hartree	
H	-0.6395	9.1301	7.2612
Al	1.5691	8.3459	6.7498
O	-0.2451	8.8199	6.4064
O	1.8848	9.3877	8.0838
O	2.3539	8.6750	5.2627
O	1.4655	6.6843	7.1747
O	-2.6096	9.2612	5.4621
O	-0.6437	8.4938	3.8146
O	-1.7243	6.7349	5.5304
O	-1.6869	8.5383	13.8567
O	-4.0642	9.2997	13.0501
O	-2.1857	8.8033	11.2521
O	-3.3083	6.7830	12.6251
O	-1.3715	8.6417	1.2506
O	1.0840	9.2819	1.9659
O	0.3451	6.7451	2.0607
O	-2.8925	8.5318	8.6695
O	-0.4837	9.2549	9.2287
O	-1.2525	6.7535	9.8214
O	-1.3681	4.9068	1.1311
O	1.0101	4.1791	1.9473
O	-0.8142	5.0174	3.7123
O	-2.9271	4.9830	8.7088
O	-0.4906	4.2403	9.3885
O	-2.2755	4.7324	11.2638
O	6.4288	11.7382	8.6855
O	7.0868	11.6990	11.2557
O	8.1759	13.5472	9.6460
O	-0.0930	4.7216	6.2609
O	-2.5496	4.2085	5.5305
O	-1.7092	4.9983	13.8653
O	6.8269	11.0086	5.5516
O	8.5982	11.7713	3.7331
O	7.6060	13.5570	5.4811
O	5.2927	11.0150	13.0943
O	6.1040	13.5519	12.9452
O	3.2639	9.3236	0.4326
O	2.9455	6.7744	-0.3825
O	0.8503	8.6886	13.0732
O	0.1008	6.7571	14.7866
O	-3.9078	8.6846	1.9896
O	-3.0811	6.7895	0.2730
O	-4.7911	9.2760	6.9799
O	-5.4156	8.6465	9.4985
O	-4.6842	6.7538	7.7632
O	4.6352	2.4248	6.9366
O	3.8857	1.8723	9.4655
O	6.4599	15.3565	8.6537

O	4.5521	16.0641	6.9609
O	3.9359	15.5008	9.4884
O	4.6696	13.5524	7.8027
O	-3.9211	4.8414	1.9257
O	-4.8021	4.2270	6.9695
O	8.0095	11.6870	1.1181
O	5.4990	11.6531	1.9752
O	6.2619	13.5472	0.2402
O	4.5605	11.0390	6.9522
O	3.8734	11.6171	9.4572
O	3.2599	4.2590	0.5004
O	1.7895	4.1803	8.0268
O	2.4321	4.8024	5.5168
O	0.8175	4.8629	13.0161
O	-4.9510	8.5124	4.4330
O	-6.4930	9.2513	2.3832
O	-5.6872	6.7547	2.5932
O	-6.3873	8.4736	11.9592
O	-7.9849	9.2514	9.9476
O	4.6036	1.6441	4.3934
O	2.9047	2.4366	2.4220
O	4.4710	15.3421	4.4038
O	5.4417	15.4840	1.9289
O	3.6887	13.5564	2.5898
O	3.0011	1.6459	11.9691
O	1.3445	2.4517	10.0108
O	2.1191	13.5745	10.0775
O	3.5065	8.4660	2.9031
O	4.9654	9.2427	5.0131
O	4.1939	6.7506	4.8281
O	1.6748	8.4487	10.5850
O	3.3811	9.2925	12.4846
O	2.6117	6.7689	12.4038
O	3.3156	5.0224	3.0337
O	4.9736	4.2468	4.9921
O	1.7855	5.0402	10.5542
O	3.3933	4.2708	12.5570
O	-6.0299	11.8572	3.0288
O	-6.9619	11.6822	5.5169
O	-4.4266	11.0855	5.0431
O	-4.8607	4.9820	4.4138
O	4.5301	11.8356	4.4329
O	2.9359	11.0581	2.4254
O	3.0249	11.9146	11.9464
O	1.3172	11.0576	10.0804
O	6.8020	16.1109	5.5058
O	-5.9484	11.0788	12.5523
O	-7.6768	11.1043	8.0521
O	-7.6559	11.8581	10.6036
O	7.9891	15.4075	1.1491

O	7.0816	15.4200	11.2480
O	8.6236	15.3374	3.7459
O	9.7057	13.5471	2.0632
Si	-1.3542	8.2835	5.2375
Si	-2.8066	8.3411	12.6936
Si	-0.1472	8.2857	2.2675
Si	-1.7109	8.3175	9.7773
Si	-0.2021	5.2121	2.2252
Si	-1.7339	5.1863	9.7968
Si	7.6418	11.9943	9.7514
Si	-1.2751	5.1670	5.2584
Si	-2.8544	5.2037	12.7154
Si	8.0849	11.9990	5.2643
Si	6.5667	11.9849	12.7726
Si	2.8927	8.3501	-0.8388
Si	-0.1333	8.3136	14.3231
Si	-2.8995	8.3537	0.7345
Si	-4.4522	8.2947	8.2373
Si	4.9645	1.5289	8.2786
Si	4.9017	15.1086	8.2352
Si	-2.9097	5.2078	0.6868
Si	-4.4809	5.1977	8.2448
Si	6.4567	11.9781	0.6860
Si	4.8815	11.9911	8.2306
Si	2.8960	5.1900	-0.8098
Si	1.4201	5.1404	6.7525
Si	-0.1419	5.2083	14.2991
Si	-5.2524	8.3015	2.8399
Si	-6.7512	8.2601	10.3764
Si	4.1718	1.4552	2.8139
Si	4.1180	15.1166	2.8244
Si	2.5980	1.4613	10.3885
Si	2.5989	15.1269	10.3754
Si	3.7296	8.2881	4.5195
Si	2.1342	8.3020	12.1380
Si	3.7178	5.2140	4.6055
Si	2.1482	5.2304	12.1311
Si	-5.6607	12.0809	4.6098
Si	-5.2364	5.1997	2.8371
Si	4.1751	12.0257	2.8551
Si	2.5895	12.0431	10.3740
Si	-4.2203	9.5303	5.4738
Si	5.2737	2.7053	5.4545
Si	5.1836	16.3550	5.4749
Si	3.7283	2.7148	12.9963
Si	2.7037	9.5166	1.9513
Si	1.1423	9.5392	9.4924
Si	2.6318	3.9819	1.9833
Si	1.1179	3.9850	9.4972
Si	-4.1606	3.9546	5.4832

Si	5.2167	10.7815	5.4778
Si	3.6742	10.8286	12.9921
Si	-5.6810	9.5249	13.0079
Si	-8.3149	10.8058	9.5314
Si	-8.0208	12.0148	6.7233
Si	6.4464	15.1156	0.6859
Si	7.6466	15.1011	9.7507
Si	-7.2129	12.0720	12.1775
Si	6.5237	15.1366	12.7634
Si	8.0733	15.1164	5.2647
Si	9.1773	11.9990	2.2261
Si	9.1779	15.0931	2.2328
Si	-6.7878	10.8263	1.9835
H	8.7647	11.0695	9.4656
H	-4.0195	4.3535	13.0402
H	9.1772	11.6540	6.2004
H	7.6611	11.6363	13.6988
H	1.5084	8.6614	-1.2908
H	3.9239	8.6070	-1.8729
H	0.1578	9.2000	15.4618
H	-3.2044	9.2208	-0.4363
H	6.3188	1.8941	8.7840
H	4.8506	0.0851	7.9305
H	-3.1956	4.3476	-0.4761
H	-5.4316	4.8988	9.3454
H	6.1172	11.1108	-0.4633
H	1.5112	4.8616	-1.2581
H	3.9194	4.9337	-1.8680
H	0.1537	4.3081	15.4316
H	-7.1601	6.8449	10.1696
H	5.3209	1.8263	1.9418
H	3.7145	0.0507	2.6279
H	2.9818	15.9774	2.4296
H	2.1729	0.0585	10.1710
H	2.9692	15.2520	11.8157
H	1.4808	16.0353	9.9784
H	-5.2267	13.4840	4.8166
H	-6.3767	4.3384	2.4678
H	6.7426	2.5348	5.4932
H	4.9022	17.7598	5.1018
H	5.2123	2.5412	12.9398
H	3.1769	2.4763	14.3628
H	-4.4052	2.5486	5.1067
H	3.0856	11.0489	14.3256
H	-6.2452	9.2813	14.3503
H	-9.7882	10.9862	9.5057
H	-9.3715	11.6454	6.2273
H	-7.9003	13.4517	7.0841
H	6.1378	15.9727	-0.4750
H	8.7668	16.0218	9.4654

H	-8.3706	11.7213	13.0571
H	-6.7659	13.4875	12.3491
H	7.6248	15.4753	13.7143
H	5.3040	15.9746	12.9789
H	9.1565	15.4638	6.2106
H	10.3029	11.0740	1.9960
H	10.3017	16.0145	1.9896
H	-8.2651	11.0116	2.0573
H	-6.2375	11.0904	0.6235

	207 atoms	Al2-O3	
Energy =		-25085.92180109	Hartree
H	2.6599	9.9501	7.9124
Al	1.3939	8.2541	6.6529
O	-0.2657	8.5298	6.3416
O	1.8352	9.4358	8.0616
O	2.4629	8.7838	5.4142
O	1.7143	6.6908	7.3051
O	-2.6295	9.2495	5.5110
O	-0.7751	8.7494	3.7268
O	-1.8494	6.7344	5.1108
O	-1.6729	8.5596	13.8365
O	-4.0606	9.3146	13.0525
O	-2.2049	8.7511	11.2394
O	-3.3275	6.7857	12.6825
O	-1.3426	8.5422	1.1373
O	1.0770	9.2294	1.9027
O	0.2774	6.7396	2.3036
O	-2.9107	8.5436	8.6697
O	-0.4619	9.2311	9.3252
O	-1.2465	6.7348	9.7751
O	-1.3769	4.9750	1.1527
O	1.0301	4.2274	1.8475
O	-0.7425	4.7221	3.7370
O	-2.9449	4.9579	8.7088
O	-0.5218	4.1971	9.4153
O	-2.2872	4.7685	11.2693
O	6.4136	11.7191	8.6952
O	7.0746	11.6992	11.2532
O	8.1668	13.5418	9.6379
O	-0.1429	5.0214	6.3140
O	-2.5428	4.2148	5.5842
O	-1.7077	4.9940	13.8691
O	6.8090	11.0508	5.6065
O	8.5766	11.7395	3.7375
O	7.6171	13.5675	5.4668
O	5.3078	11.0136	13.1128
O	6.1013	13.5538	12.9425
O	3.2949	9.2923	0.4527
O	2.9391	6.7695	-0.3836
O	0.8632	8.6900	13.0461
O	0.1145	6.7610	14.7583
O	-3.8745	8.6826	1.9967
O	-3.1640	6.7755	0.2484
O	-4.8349	9.2777	6.9784
O	-5.4203	8.6425	9.4985
O	-4.6810	6.7523	7.7636
O	4.6344	2.4189	6.9387
O	3.8888	1.8729	9.4709
O	6.4632	15.3478	8.6439

O	4.5453	16.0501	6.9529
O	3.9484	15.5165	9.4877
O	4.6589	13.5425	7.8217
O	-3.9231	4.8129	1.9274
O	-4.8227	4.2204	6.9678
O	8.0024	11.6886	1.1249
O	5.4893	11.6516	1.9792
O	6.2563	13.5422	0.2381
O	4.5218	11.0023	6.9543
O	3.8173	11.5458	9.3896
O	3.3044	4.2603	0.4921
O	1.7148	4.1331	7.9969
O	2.3699	4.8071	5.5040
O	0.8160	4.8519	13.0073
O	-4.9692	8.5340	4.4307
O	-6.4744	9.2433	2.3557
O	-5.6626	6.7543	2.5912
O	-6.3826	8.4725	11.9603
O	-7.9875	9.2540	9.9567
O	4.5996	1.6514	4.3883
O	2.8992	2.4451	2.4124
O	4.4749	15.3368	4.4004
O	5.4374	15.4714	1.9272
O	3.6776	13.5460	2.6030
O	3.0054	1.6286	11.9734
O	1.3428	2.4386	10.0218
O	2.1673	13.5814	10.0773
O	3.4583	8.4733	2.9621
O	5.0346	9.2191	4.9995
O	4.1539	6.7453	4.8558
O	1.8055	8.5144	10.5718
O	3.4244	9.2569	12.5414
O	2.6121	6.7472	12.3747
O	3.3067	5.0208	3.0371
O	4.9384	4.2500	5.0159
O	1.7677	5.0288	10.5303
O	3.3868	4.2558	12.5342
O	-6.0297	11.8507	3.0304
O	-6.9689	11.6882	5.5110
O	-4.4289	11.0927	5.0588
O	-4.8521	4.9727	4.4146
O	4.5074	11.8061	4.4322
O	2.9164	11.0446	2.4124
O	3.0553	11.8685	11.9268
O	1.3002	11.1086	10.0542
O	6.8001	16.1144	5.5178
O	-5.9501	11.0812	12.5532
O	-7.6778	11.1048	8.0519
O	-7.6578	11.8615	10.6024
O	7.9874	15.3978	1.1475

O	7.0761	15.4149	11.2424
O	8.6191	15.3703	3.7441
O	9.6925	13.5431	2.0990
Si	-1.3529	8.3028	5.1903
Si	-2.8170	8.3401	12.6939
Si	-0.2009	8.3043	2.2811
Si	-1.7339	8.2897	9.7507
Si	-0.2119	5.1802	2.2726
Si	-1.7524	5.1744	9.7856
Si	7.6392	11.9900	9.7522
Si	-1.3049	5.1946	5.1933
Si	-2.8621	5.2105	12.7270
Si	8.0839	12.0053	5.2677
Si	6.5722	11.9900	12.7760
Si	2.8941	8.3462	-0.8342
Si	-0.1280	8.3190	14.3075
Si	-2.9081	8.3300	0.7132
Si	-4.4651	8.3013	8.2237
Si	4.9646	1.5271	8.2805
Si	4.9044	15.1105	8.2303
Si	-2.9250	5.2115	0.6886
Si	-4.4943	5.1943	8.2363
Si	6.4482	11.9742	0.6891
Si	4.8824	11.9805	8.2160
Si	2.8991	5.1851	-0.8115
Si	1.4207	5.1964	6.7817
Si	-0.1407	5.2041	14.2919
Si	-5.2302	8.3044	2.8382
Si	-6.7518	8.2600	10.3754
Si	4.1639	1.4613	2.8081
Si	4.1124	15.1064	2.8212
Si	2.6014	1.4550	10.3907
Si	2.6132	15.1461	10.3745
Si	3.7400	8.2963	4.5664
Si	2.1712	8.2887	12.1552
Si	3.6777	5.2086	4.6157
Si	2.1369	5.2056	12.1058
Si	-5.6574	12.0779	4.6112
Si	-5.2309	5.1959	2.8400
Si	4.1526	12.0071	2.8483
Si	2.6050	12.0509	10.3737
Si	-4.2157	9.5307	5.4819
Si	5.2668	2.7076	5.4549
Si	5.1821	16.3532	5.4677
Si	3.7275	2.7051	12.9932
Si	2.6816	9.4917	1.9525
Si	1.0825	9.5717	9.5860
Si	2.6380	3.9928	1.9617
Si	1.0872	3.9586	9.4866
Si	-4.1517	3.9535	5.4899

Si	5.2161	10.7624	5.4664
Si	3.6966	10.8062	13.0098
Si	-5.6820	9.5272	13.0065
Si	-8.3150	10.8060	9.5294
Si	-8.0201	12.0149	6.7205
Si	6.4433	15.1095	0.6827
Si	7.6467	15.0998	9.7467
Si	-7.2152	12.0728	12.1749
Si	6.5242	15.1393	12.7622
Si	8.0761	15.1299	5.2599
Si	9.1684	11.9906	2.2349
Si	9.1746	15.0960	2.2352
Si	-6.7841	10.8199	1.9822
H	8.7647	11.0695	9.4656
H	-4.0195	4.3535	13.0402
H	9.1772	11.6540	6.2004
H	7.6611	11.6363	13.6988
H	1.5084	8.6614	-1.2908
H	3.9239	8.6070	-1.8729
H	0.1578	9.2000	15.4618
H	-3.2044	9.2208	-0.4363
H	6.3188	1.8941	8.7840
H	4.8506	0.0851	7.9305
H	-3.1956	4.3476	-0.4761
H	-5.4316	4.8988	9.3454
H	6.1172	11.1108	-0.4633
H	1.5112	4.8616	-1.2581
H	3.9194	4.9337	-1.8680
H	0.1537	4.3081	15.4316
H	-7.1601	6.8449	10.1696
H	5.3209	1.8263	1.9418
H	3.7145	0.0507	2.6279
H	2.9818	15.9774	2.4296
H	2.1729	0.0585	10.1710
H	2.9692	15.2520	11.8157
H	1.4808	16.0353	9.9784
H	-5.2267	13.4840	4.8166
H	-6.3767	4.3384	2.4678
H	6.7426	2.5348	5.4932
H	4.9022	17.7598	5.1018
H	5.2123	2.5412	12.9398
H	3.1769	2.4763	14.3628
H	-4.4052	2.5486	5.1067
H	3.0856	11.0489	14.3256
H	-6.2452	9.2813	14.3503
H	-9.7882	10.9862	9.5057
H	-9.3715	11.6454	6.2273
H	-7.9003	13.4517	7.0841
H	6.1378	15.9727	-0.4750
H	8.7668	16.0218	9.4654

H	-8.3706	11.7213	13.0571
H	-6.7659	13.4875	12.3491
H	7.6248	15.4753	13.7143
H	5.3040	15.9746	12.9789
H	9.1565	15.4638	6.2106
H	10.3029	11.0740	1.9960
H	10.3017	16.0145	1.9896
H	-8.2651	11.0116	2.0573
H	-6.2375	11.0904	0.6235

207	atoms	Al2-O6	
Energy =		-25085.91391354	Hartree
H	1.3841	8.8489	4.6421
Al	1.3008	8.2515	6.9352
O	-0.3533	8.3836	6.4578
O	1.7681	9.4179	8.1005
O	2.1388	8.6980	5.2729
O	1.8237	6.6432	7.2340
O	-2.5789	9.2598	5.2880
O	-0.3954	8.6873	3.8700
O	-1.8685	6.7211	4.9688
O	-1.6663	8.6058	13.8551
O	-4.0525	9.3719	13.0821
O	-2.3040	8.5482	11.2694
O	-3.4220	6.7998	12.9496
O	-1.4296	8.6153	1.3313
O	1.0711	9.2320	1.8031
O	0.2725	6.7239	2.2138
O	-2.8962	8.5552	8.6891
O	-0.5228	9.3691	9.5027
O	-1.1513	6.7722	9.6418
O	-1.3872	4.9219	1.1548
O	1.0417	4.2156	1.7907
O	-0.6670	4.7077	3.7270
O	-2.9276	4.9754	8.7110
O	-0.5534	4.1832	9.5213
O	-2.3411	5.0146	11.2772
O	6.4565	11.7658	8.6575
O	7.0705	11.6951	11.2472
O	8.1861	13.5536	9.6635
O	-0.1726	5.0868	6.3127
O	-2.5339	4.2070	5.5297
O	-1.6973	4.9437	13.8637
O	6.7894	10.9742	5.4938
O	8.6034	11.7683	3.7410
O	7.5879	13.5374	5.4934
O	5.3100	11.0096	13.1205
O	6.1092	13.5526	12.9372
O	3.3301	9.2968	0.4260
O	2.9372	6.7689	-0.3807
O	0.8770	8.6825	13.0732
O	0.0763	6.7579	14.7643
O	-3.9725	8.6897	1.9630
O	-3.0960	6.7887	0.2599
O	-4.7199	9.2772	6.9421
O	-5.4452	8.6619	9.4410
O	-4.6644	6.7580	7.7559
O	4.6365	2.4253	6.9421
O	3.8787	1.8753	9.4656
O	6.4733	15.3649	8.6514

O	4.5590	16.0788	6.9635
O	3.9525	15.5628	9.5006
O	4.6594	13.5827	7.8357
O	-3.9352	4.8482	1.9261
O	-4.7919	4.2249	6.9719
O	7.9922	11.6696	1.1393
O	5.4664	11.6385	1.9622
O	6.2642	13.5448	0.2515
O	4.5499	11.0951	6.9665
O	3.9233	11.6212	9.4942
O	3.3313	4.2657	0.4887
O	1.6309	4.1098	8.0058
O	2.3202	4.6933	5.4950
O	0.8281	4.8656	13.0087
O	-4.9950	8.5410	4.4237
O	-6.5411	9.2392	2.3690
O	-5.7153	6.7491	2.6046
O	-6.3355	8.4775	11.9401
O	-7.9893	9.2572	9.9722
O	4.5946	1.6756	4.3862
O	2.9018	2.4401	2.4009
O	4.4653	15.3351	4.4087
O	5.4367	15.4754	1.9323
O	3.6720	13.5556	2.5902
O	3.0129	1.6305	11.9748
O	1.3310	2.4279	10.0350
O	2.1834	13.5934	10.0654
O	3.3666	8.4885	2.9567
O	4.7912	9.2354	5.1320
O	3.9861	6.7442	4.8966
O	1.7438	8.4924	10.5803
O	3.4102	9.2885	12.5077
O	2.6281	6.7629	12.3760
O	3.2836	5.0177	3.0392
O	4.9218	4.2724	5.0288
O	1.7859	5.0211	10.5326
O	3.3941	4.2625	12.5375
O	-6.0289	11.8449	3.0301
O	-6.9532	11.6805	5.5213
O	-4.4049	11.0987	5.0289
O	-4.8585	4.9750	4.4181
O	4.4709	11.7913	4.4116
O	2.8933	11.0584	2.3890
O	3.0682	11.9366	11.9696
O	1.3506	11.1105	10.1129
O	6.8002	16.0974	5.4950
O	-5.9599	11.0870	12.5505
O	-7.6792	11.0987	8.0528
O	-7.6644	11.8715	10.5990
O	7.9853	15.4135	1.1572

O	7.0785	15.4317	11.2494
O	8.6284	15.3024	3.7524
O	9.6985	13.5401	2.0471
Si	-1.3171	8.2511	5.1964
Si	-2.8520	8.3254	12.7766
Si	-0.1646	8.2925	2.2921
Si	-1.7117	8.3094	9.7764
Si	-0.2005	5.1534	2.2395
Si	-1.7400	5.2507	9.7879
Si	7.6489	12.0014	9.7556
Si	-1.3004	5.1900	5.1523
Si	-2.8903	5.2570	12.7885
Si	8.0663	11.9798	5.2659
Si	6.5689	11.9840	12.7736
Si	2.8964	8.3379	-0.8484
Si	-0.1250	8.3244	14.3145
Si	-2.9397	8.3481	0.7436
Si	-4.4314	8.3077	8.2173
Si	4.9614	1.5275	8.2865
Si	4.9074	15.1409	8.2514
Si	-2.9257	5.2112	0.6905
Si	-4.4780	5.2044	8.2451
Si	6.4489	11.9742	0.6888
Si	4.8956	12.0260	8.2578
Si	2.9020	5.1820	-0.8130
Si	1.4016	5.1642	6.7780
Si	-0.1388	5.2009	14.2912
Si	-5.2977	8.3022	2.8430
Si	-6.7412	8.2646	10.3645
Si	4.1672	1.4598	2.8088
Si	4.1126	15.1164	2.8290
Si	2.5974	1.4550	10.3981
Si	2.6153	15.1508	10.3759
Si	3.6358	8.2745	4.5400
Si	2.1611	8.3053	12.1344
Si	3.6143	5.1641	4.6312
Si	2.1510	5.2278	12.1111
Si	-5.6517	12.0765	4.6110
Si	-5.2463	5.2019	2.8486
Si	4.1420	12.0208	2.8328
Si	2.6316	12.0646	10.3994
Si	-4.1737	9.5342	5.4280
Si	5.2654	2.7207	5.4625
Si	5.1833	16.3548	5.4745
Si	3.7292	2.7136	12.9956
Si	2.6686	9.5263	1.8889
Si	1.0990	9.5944	9.5519
Si	2.6450	3.9828	1.9455
Si	1.0589	3.9536	9.5250
Si	-4.1494	3.9533	5.4875

Si	5.1699	10.7956	5.4977
Si	3.6911	10.8260	13.0067
Si	-5.6751	9.5391	13.0097
Si	-8.3158	10.8059	9.5341
Si	-8.0170	12.0118	6.7234
Si	6.4453	15.1173	0.6896
Si	7.6507	15.1064	9.7586
Si	-7.2205	12.0777	12.1730
Si	6.5231	15.1384	12.7649
Si	8.0696	15.0977	5.2709
Si	9.1744	11.9941	2.2294
Si	9.1786	15.0878	2.2337
Si	-6.7941	10.8241	1.9825
H	8.7647	11.0695	9.4656
H	-4.0195	4.3535	13.0402
H	9.1772	11.6540	6.2004
H	7.6611	11.6363	13.6988
H	1.5084	8.6614	-1.2908
H	3.9239	8.6070	-1.8729
H	0.1578	9.2000	15.4618
H	-3.2044	9.2208	-0.4363
H	6.3188	1.8941	8.7840
H	4.8506	0.0851	7.9305
H	-3.1956	4.3476	-0.4761
H	-5.4316	4.8988	9.3454
H	6.1172	11.1108	-0.4633
H	1.5112	4.8616	-1.2581
H	3.9194	4.9337	-1.8680
H	0.1537	4.3081	15.4316
H	-7.1601	6.8449	10.1696
H	5.3209	1.8263	1.9418
H	3.7145	0.0507	2.6279
H	2.9818	15.9774	2.4296
H	2.1729	0.0585	10.1710
H	2.9692	15.2520	11.8157
H	1.4808	16.0353	9.9784
H	-5.2267	13.4840	4.8166
H	-6.3767	4.3384	2.4678
H	6.7426	2.5348	5.4932
H	4.9022	17.7598	5.1018
H	5.2123	2.5412	12.9398
H	3.1769	2.4763	14.3628
H	-4.4052	2.5486	5.1067
H	3.0856	11.0489	14.3256
H	-6.2452	9.2813	14.3503
H	-9.7882	10.9862	9.5057
H	-9.3715	11.6454	6.2273
H	-7.9003	13.4517	7.0841
H	6.1378	15.9727	-0.4750
H	8.7668	16.0218	9.4654

H	-8.3706	11.7213	13.0571
H	-6.7659	13.4875	12.3491
H	7.6248	15.4753	13.7143
H	5.3040	15.9746	12.9789
H	9.1565	15.4638	6.2106
H	10.3029	11.0740	1.9960
H	10.3017	16.0145	1.9896
H	-8.2651	11.0116	2.0573
H	-6.2375	11.0904	0.6235

	207 atoms	Al2-O9	
Energy =	-25085.92145792	Hartree	
H	1.9725	6.7400	8.3744
Al	1.3652	8.4765	6.7129
O	-0.3009	8.3612	6.3405
O	1.7519	9.4133	8.0979
O	2.3648	8.5953	5.3318
O	1.7149	6.7244	7.4302
O	-2.6263	9.2457	5.5067
O	-0.7622	8.7661	3.7280
O	-1.9203	6.7237	5.0010
O	-1.6714	8.6106	13.8437
O	-4.0605	9.3685	13.0661
O	-2.3123	8.5310	11.2561
O	-3.4225	6.7980	12.9511
O	-1.3379	8.5357	1.1405
O	1.0899	9.2137	1.8955
O	0.2688	6.7323	2.3200
O	-2.9182	8.5665	8.6708
O	-0.5434	9.3766	9.4940
O	-1.1598	6.7772	9.6136
O	-1.3887	4.9677	1.1716
O	1.0226	4.2174	1.8259
O	-0.6968	4.6909	3.7522
O	-2.9386	4.9696	8.7091
O	-0.5578	4.1929	9.5080
O	-2.3275	5.0216	11.2782
O	6.4430	11.7527	8.6714
O	7.0740	11.6894	11.2506
O	8.1825	13.5485	9.6635
O	-0.1631	5.1552	6.3123
O	-2.5259	4.2002	5.5876
O	-1.6970	4.9393	13.8618
O	6.8211	11.0147	5.5413
O	8.6082	11.7798	3.7384
O	7.6032	13.5588	5.4880
O	5.3084	11.0046	13.1189
O	6.1071	13.5506	12.9397
O	3.2982	9.3063	0.4415
O	2.9383	6.7740	-0.3809
O	0.8632	8.6839	13.0517
O	0.0766	6.7632	14.7511
O	-3.8709	8.6778	1.9974
O	-3.1592	6.7751	0.2471
O	-4.8379	9.2818	6.9722
O	-5.4348	8.6508	9.4888
O	-4.6787	6.7613	7.7649
O	4.6280	2.4137	6.9398
O	3.8786	1.8795	9.4635
O	6.4684	15.3524	8.6508

O	4.5593	16.0677	6.9592
O	3.9484	15.5351	9.4926
O	4.6597	13.5632	7.8204
O	-3.9297	4.8100	1.9241
O	-4.8069	4.2254	6.9713
O	7.9979	11.6749	1.1321
O	5.4769	11.6497	1.9676
O	6.2691	13.5444	0.2416
O	4.5595	11.0561	6.9542
O	3.9000	11.6089	9.4700
O	3.3103	4.2708	0.4982
O	1.6529	4.1743	8.0345
O	2.3848	4.9287	5.5582
O	0.8267	4.8677	13.0039
O	-4.9658	8.5346	4.4295
O	-6.4716	9.2431	2.3524
O	-5.6627	6.7543	2.5956
O	-6.3661	8.4716	11.9608
O	-7.9922	9.2575	9.9715
O	4.5984	1.6406	4.3883
O	2.9001	2.4449	2.4135
O	4.4705	15.3368	4.4037
O	5.4353	15.4721	1.9282
O	3.6713	13.5508	2.5920
O	3.0085	1.6339	11.9750
O	1.3378	2.4355	10.0318
O	2.1613	13.5856	10.0666
O	3.4853	8.4728	2.9372
O	4.9586	9.2436	5.0382
O	4.2321	6.7469	4.8159
O	1.7225	8.4447	10.5438
O	3.3864	9.3095	12.4686
O	2.6308	6.7736	12.3684
O	3.2598	5.0123	3.0601
O	4.9103	4.2323	5.0255
O	1.7817	5.0522	10.5242
O	3.3892	4.2619	12.5224
O	-6.0306	11.8498	3.0314
O	-6.9645	11.6852	5.5149
O	-4.4231	11.0942	5.0514
O	-4.8357	4.9707	4.4136
O	4.5104	11.8237	4.4257
O	2.9113	11.0456	2.4195
O	3.0783	11.9542	11.9633
O	1.3512	11.0828	10.1460
O	6.8038	16.1135	5.4964
O	-5.9622	11.0863	12.5468
O	-7.6796	11.0991	8.0520
O	-7.6655	11.8717	10.5980
O	7.9847	15.4146	1.1531

O	7.0788	15.4272	11.2481
O	8.6312	15.3264	3.7469
O	9.7012	13.5455	2.0481
Si	-1.3752	8.2771	5.1617
Si	-2.8592	8.3255	12.7661
Si	-0.1930	8.3051	2.2832
Si	-1.7344	8.3159	9.7539
Si	-0.2104	5.1737	2.2718
Si	-1.7557	5.2604	9.7798
Si	7.6443	11.9967	9.7532
Si	-1.3339	5.2133	5.1556
Si	-2.8913	5.2580	12.7880
Si	8.0842	12.0029	5.2658
Si	6.5698	11.9835	12.7725
Si	2.8947	8.3524	-0.8361
Si	-0.1310	8.3321	14.3048
Si	-2.9042	8.3305	0.7138
Si	-4.4655	8.3153	8.2258
Si	4.9646	1.5244	8.2836
Si	4.9056	15.1210	8.2396
Si	-2.9309	5.2119	0.6888
Si	-4.4916	5.2071	8.2403
Si	6.4491	11.9743	0.6893
Si	4.8895	12.0022	8.2348
Si	2.8998	5.1920	-0.8119
Si	1.3771	5.1717	6.7872
Si	-0.1421	5.2039	14.2918
Si	-5.2291	8.3059	2.8385
Si	-6.7532	8.2626	10.3800
Si	4.1643	1.4584	2.8048
Si	4.1111	15.1091	2.8250
Si	2.6051	1.4523	10.3982
Si	2.6114	15.1427	10.3733
Si	3.7285	8.2856	4.5465
Si	2.1471	8.3140	12.1124
Si	3.6990	5.2351	4.6085
Si	2.1505	5.2399	12.1188
Si	-5.6564	12.0786	4.6121
Si	-5.2323	5.1971	2.8418
Si	4.1506	12.0158	2.8476
Si	2.6291	12.0618	10.3929
Si	-4.2146	9.5345	5.4804
Si	5.2646	2.6936	5.4554
Si	5.1844	16.3543	5.4699
Si	3.7293	2.7153	12.9958
Si	2.6931	9.4980	1.9456
Si	1.0769	9.5915	9.5414
Si	2.6288	3.9885	1.9549
Si	1.0453	3.9565	9.5493
Si	-4.1465	3.9517	5.4934

Si	5.2105	10.7896	5.4791
Si	3.6894	10.8387	12.9986
Si	-5.6850	9.5395	13.0093
Si	-8.3168	10.8071	9.5317
Si	-8.0193	12.0132	6.7226
Si	6.4443	15.1140	0.6864
Si	7.6492	15.1023	9.7545
Si	-7.2228	12.0791	12.1711
Si	6.5234	15.1374	12.7631
Si	8.0742	15.1164	5.2642
Si	9.1756	11.9982	2.2255
Si	9.1775	15.0920	2.2295
Si	-6.7842	10.8211	1.9821
H	8.7647	11.0695	9.4656
H	-4.0195	4.3535	13.0402
H	9.1772	11.6540	6.2004
H	7.6611	11.6363	13.6988
H	1.5084	8.6614	-1.2908
H	3.9239	8.6070	-1.8729
H	0.1578	9.2000	15.4618
H	-3.2044	9.2208	-0.4363
H	6.3188	1.8941	8.7840
H	4.8506	0.0851	7.9305
H	-3.1956	4.3476	-0.4761
H	-5.4316	4.8988	9.3454
H	6.1172	11.1108	-0.4633
H	1.5112	4.8616	-1.2581
H	3.9194	4.9337	-1.8680
H	0.1537	4.3081	15.4316
H	-7.1601	6.8449	10.1696
H	5.3209	1.8263	1.9418
H	3.7145	0.0507	2.6279
H	2.9818	15.9774	2.4296
H	2.1729	0.0585	10.1710
H	2.9692	15.2520	11.8157
H	1.4808	16.0353	9.9784
H	-5.2267	13.4840	4.8166
H	-6.3767	4.3384	2.4678
H	6.7426	2.5348	5.4932
H	4.9022	17.7598	5.1018
H	5.2123	2.5412	12.9398
H	3.1769	2.4763	14.3628
H	-4.4052	2.5486	5.1067
H	3.0856	11.0489	14.3256
H	-6.2452	9.2813	14.3503
H	-9.7882	10.9862	9.5057
H	-9.3715	11.6454	6.2273
H	-7.9003	13.4517	7.0841
H	6.1378	15.9727	-0.4750
H	8.7668	16.0218	9.4654

H	-8.3706	11.7213	13.0571
H	-6.7659	13.4875	12.3491
H	7.6248	15.4753	13.7143
H	5.3040	15.9746	12.9789
H	9.1565	15.4638	6.2106
H	10.3029	11.0740	1.9960
H	10.3017	16.0145	1.9896
H	-8.2651	11.0116	2.0573
H	-6.2375	11.0904	0.6235

	216 atoms	Al3-O5	
Energy =	-25743.45624326	Hartree	
H	13.9123	7.3931	4.9590
Al	13.4537	8.2960	2.7203
O	13.8794	8.3041	4.5869
O	14.9517	8.7554	2.0006
O	12.1893	9.4343	2.5868
O	12.9771	6.6621	2.5026
O	20.1513	8.6049	-1.2643
O	17.7477	9.3793	-2.0096
O	18.3778	6.7750	-2.1282
O	18.5558	8.5690	6.3013
O	16.1776	9.3575	5.5134
O	17.9137	8.4988	3.7111
O	16.7684	6.7753	5.4094
O	9.1639	1.9314	6.3558
O	8.5524	1.7345	3.7447
O	9.1614	15.3612	6.3565
O	8.5681	15.5913	3.7672
O	7.5306	13.5551	5.1907
O	17.4308	8.5183	1.0801
O	19.7388	9.3515	2.0098
O	19.1462	6.7537	2.1168
O	15.8113	8.5396	8.7411
O	18.1750	9.3756	9.5691
O	17.5769	6.7619	9.7006
O	10.3398	2.5108	1.8975
O	10.3811	16.0825	1.9047
O	9.4560	13.5980	2.2076
O	17.3578	4.9873	1.1555
O	19.7077	4.1597	1.9931
O	17.9356	5.0362	3.7477
O	15.8044	5.0225	8.6913
O	18.1554	4.1505	9.5643
O	7.8804	11.6616	1.2009
O	10.3393	11.1332	1.8050
O	8.6318	11.5878	3.7608
O	20.1391	4.9345	-1.2688
O	17.7205	4.1826	-2.0052
O	18.5425	4.9899	6.3351
O	16.1760	4.1792	5.5248
O	10.7392	11.6207	-1.2013
O	8.3185	10.9522	-1.9972
O	10.0679	11.8506	-3.7665
O	9.1627	11.7837	6.3575
O	6.7819	11.0244	5.5684
O	21.9831	9.3007	0.5376
O	21.8373	6.7665	-0.2834
O	20.4146	9.2742	8.0632
O	21.1008	8.7440	5.5542

O	20.3280	6.7763	7.2033
O	12.6190	2.3977	0.5312
O	13.2492	1.8583	-2.0253
O	12.6454	16.0755	0.5202
O	13.2675	15.5387	-2.0077
O	12.3492	13.5956	-0.3815
O	11.0902	2.3690	8.1141
O	11.7168	1.8839	5.5574
O	11.7112	15.4932	5.5465
O	10.9496	13.5744	7.2553
O	15.5005	9.2664	-0.5672
O	15.5620	6.7725	0.3014
O	13.9661	9.3406	7.0309
O	13.2689	8.7000	9.5104
O	14.0292	6.8004	7.7513
O	15.4747	4.2697	-0.5662
O	14.8430	4.7733	1.9610
O	13.9332	4.2857	6.9402
O	13.2775	4.8584	9.4582
O	6.0068	11.1224	-0.6092
O	5.3152	11.6253	1.9039
O	20.4118	4.2796	8.0784
O	21.0886	4.7949	5.5667
O	12.6650	11.1370	0.5566
O	13.2671	11.6497	-1.9734
O	11.0353	11.0785	8.0829
O	11.7000	11.6354	5.5679
O	15.4882	8.4758	-3.1066
O	13.7882	9.3004	-5.0114
O	14.4953	6.7702	-4.8836
O	10.6657	9.2432	9.9773
O	11.5051	6.7646	10.1189
O	14.2021	1.6486	-4.4950
O	15.8112	2.4398	-2.4781
O	15.0535	13.5788	-2.5764
O	12.5587	1.5151	3.0480
O	14.2123	2.5147	4.9424
O	12.6916	15.3342	3.0868
O	13.4927	13.5565	4.9547
O	14.1479	11.9151	-4.4469
O	15.8105	11.0679	-2.5120
O	12.5203	12.0330	3.0527
O	14.1542	10.9524	4.8823
O	15.4357	5.0148	-3.1219
O	13.7465	4.2600	-5.0710
O	13.9343	5.1551	4.4310
O	12.3227	4.1143	2.5615
O	10.6878	4.2853	9.9460
O	22.0656	8.5542	3.0810
O	21.9631	4.2352	0.5370

O	22.0253	4.9804	3.0861
O	11.8645	11.0049	-5.5878
O	4.4291	11.7813	4.4401
O	22.8075	6.7627	4.9152
Si	18.9365	8.3118	-2.3132
Si	17.3784	8.2894	5.2182
Si	7.9904	1.5440	5.2658
Si	8.0021	15.1241	5.2240
Si	18.5473	8.2766	2.2250
Si	16.9919	8.2942	9.8470
Si	9.0756	1.5096	2.2130
Si	9.0703	15.1845	2.2667
Si	18.5287	5.2418	2.2481
Si	16.9672	5.2338	9.8233
Si	9.0683	12.0112	2.2513
Si	18.9232	5.2353	-2.3157
Si	17.3649	5.2466	5.2443
Si	9.5419	12.0070	-2.2370
Si	8.0256	11.9953	5.2092
Si	21.6762	8.3299	-0.7440
Si	20.1020	8.3340	6.7719
Si	12.2087	1.5220	-0.8058
Si	12.2398	15.1732	-0.7919
Si	10.6917	1.5199	6.7744
Si	10.7185	15.1304	6.7990
Si	15.8391	8.3343	0.7314
Si	14.2668	8.3313	8.2856
Si	15.8010	5.2189	0.7171
Si	14.2601	5.2382	8.2180
Si	6.3641	12.0114	0.7159
Si	20.0938	5.2147	6.7852
Si	12.2556	12.0094	-0.7518
Si	10.7099	12.0232	6.8008
Si	15.0317	8.2961	-4.6663
Si	11.9381	8.3128	10.3955
Si	14.5704	1.4570	-2.9065
Si	14.5902	15.1287	-2.8985
Si	12.9939	1.4560	4.6252
Si	13.0474	15.1175	4.6678
Si	14.5652	12.0528	-2.8688
Si	12.9538	12.0525	4.6109
Si	15.0048	5.2322	-4.6862
Si	13.5123	5.1935	2.8210
Si	11.9597	5.2157	10.3698
Si	16.1258	9.5378	-2.0424
Si	14.5734	9.5452	5.5409
Si	21.3556	9.5863	2.0239
Si	11.9629	2.6350	2.0044
Si	11.9962	16.3489	1.9996
Si	21.3298	3.9464	2.0204

Si	13.4867	10.8351	-5.4965
Si	11.9365	10.9016	2.0047
Si	10.3799	10.8148	9.5731
Si	16.1024	3.9866	-2.0448
Si	14.5546	4.0245	5.4479
Si	6.6887	10.7520	-2.0679
Si	5.1636	10.7463	5.4934
Si	10.6103	12.0270	-5.2946
Si	4.0462	12.0431	2.8611
Si	21.6659	5.2055	-0.7474
Si	22.3987	5.1985	4.6603
Si	22.4154	8.3303	4.6586
Si	19.7957	3.9687	9.5759
Si	19.8125	9.5721	9.5688
Si	10.3909	2.7099	9.5679
Si	13.4301	2.7028	-5.5098
H	19.4098	8.5047	-3.7003
H	6.8483	2.4770	5.4771
H	7.6152	0.1144	5.4642
H	6.8356	15.9497	5.5498
H	16.4509	8.5030	11.2037
H	7.9600	1.8765	1.3028
H	9.5328	0.0973	2.0347
H	7.9902	15.4883	1.3083
H	16.4153	5.0210	11.1808
H	19.3989	5.0322	-3.7018
H	9.1128	13.4122	-2.0000
H	22.6392	8.6367	-1.8203
H	10.8405	1.9116	-1.2538
H	12.3135	0.0773	-0.4619
H	10.8542	15.4696	-1.2285
H	10.7908	0.0783	7.0769
H	11.0035	15.9916	7.9593
H	6.3080	13.4572	0.4135
H	16.1807	8.5663	-5.5698
H	12.2883	8.5121	11.8158
H	14.9992	0.0566	-2.6754
H	14.2084	15.2183	-4.3346
H	15.7246	16.0285	-2.5370
H	13.4314	0.0625	4.8742
H	14.1620	16.0103	5.0553
H	16.1679	4.9661	-5.5748
H	12.3189	5.0027	11.7930
H	21.6028	10.9887	2.4146
H	12.2358	17.7629	2.3849
H	21.5928	2.5482	2.4104
H	14.0879	11.0692	-6.8239
H	8.9032	11.0352	9.5340
H	11.0663	11.6868	10.5706
H	6.1289	11.6798	-3.0990

H	6.3979	9.3208	-2.3711
H	4.6188	11.0068	6.8552
H	4.9604	9.3325	5.0474
H	9.4969	11.6715	-6.2172
H	11.0818	13.4285	-5.4557
H	2.8975	11.1758	2.4777
H	3.7516	13.4986	2.6862
H	22.6317	4.8982	-1.8247
H	23.5491	4.3478	5.0291
H	23.5619	9.1791	5.0342
H	20.3733	4.9250	10.5698
H	20.1043	2.5466	9.8996
H	20.3938	8.6184	10.5621
H	20.1088	10.9951	9.8944
H	8.9131	2.5082	9.4848
H	11.0270	1.8583	10.6130
H	11.9483	2.5112	-5.4377
H	13.9687	2.4757	-6.8835

	216 atoms	Al3-O6	
Energy =		-25743.44793760	Hartree
H	15.3512	9.6065	2.4313
Al	13.3586	8.2496	2.9427
O	13.9064	8.4973	4.5502
O	14.9525	8.8316	1.9907
O	12.1488	9.3258	2.4017
O	13.1541	6.6401	2.4265
O	20.1459	8.5988	-1.2661
O	17.7413	9.3696	-2.0049
O	18.3674	6.7716	-2.1269
O	18.5819	8.5839	6.2718
O	16.1837	9.3881	5.5943
O	17.8070	8.5428	3.7063
O	16.8006	6.7756	5.4276
O	9.1634	1.9313	6.3561
O	8.5496	1.7347	3.7439
O	9.1549	15.3590	6.3614
O	8.5721	15.6197	3.7702
O	7.5358	13.5605	5.1636
O	17.4252	8.5363	1.1035
O	19.7250	9.3580	2.0509
O	19.1252	6.7651	2.1671
O	15.8176	8.5251	8.7402
O	18.1769	9.3712	9.5669
O	17.5880	6.7591	9.7085
O	10.3464	2.4967	1.8963
O	10.3744	16.0949	1.8863
O	9.4585	13.6138	2.2330
O	17.3789	5.0158	1.1418
O	19.7128	4.1760	2.0013
O	17.9043	5.0133	3.7523
O	15.8075	5.0186	8.6946
O	18.1577	4.1500	9.5606
O	7.9032	11.6769	1.1971
O	10.3772	11.1782	1.8258
O	8.6309	11.5758	3.7585
O	20.1386	4.9402	-1.2687
O	17.7234	4.1759	-2.0105
O	18.5554	4.9670	6.3300
O	16.1743	4.1789	5.5533
O	10.7374	11.6265	-1.1909
O	8.3223	10.9484	-2.0046
O	10.0790	11.8520	-3.7604
O	9.1652	11.8018	6.3545
O	6.7820	11.0337	5.5698
O	21.9545	9.3010	0.5417
O	21.8358	6.7665	-0.2829
O	20.4226	9.2718	8.0664
O	21.1159	8.7421	5.5595

O	20.3274	6.7747	7.1994
O	12.6263	2.3872	0.5288
O	13.2438	1.8568	-2.0306
O	12.6452	16.0893	0.5115
O	13.2661	15.5455	-2.0142
O	12.3578	13.6058	-0.3889
O	11.0885	2.3696	8.1164
O	11.7121	1.8833	5.5550
O	11.6920	15.5525	5.5338
O	10.9757	13.5846	7.2164
O	15.4963	9.2208	-0.5867
O	15.4937	6.7487	0.4280
O	13.9550	9.3661	7.0713
O	13.2909	8.7172	9.5439
O	14.0082	6.7987	7.8121
O	15.4831	4.2946	-0.5771
O	14.8764	4.6489	1.9808
O	13.9279	4.2816	6.9650
O	13.2834	4.8276	9.4797
O	6.0222	11.1211	-0.6024
O	5.3277	11.6272	1.9094
O	20.4237	4.2796	8.0821
O	21.0939	4.7985	5.5658
O	12.6934	11.1570	0.5512
O	13.2563	11.6547	-1.9833
O	11.0329	11.0966	8.0811
O	11.6941	11.6238	5.5472
O	15.4806	8.4752	-3.1214
O	13.7800	9.2954	-5.0223
O	14.4897	6.7656	-4.8937
O	10.6727	9.2430	9.9673
O	11.5196	6.7657	10.1122
O	14.2045	1.6434	-4.4982
O	15.8026	2.4458	-2.4807
O	15.0527	13.5803	-2.5780
O	12.6005	1.5779	3.0645
O	14.2555	2.4506	5.0365
O	12.6815	15.3456	3.0746
O	13.4380	13.5902	4.9420
O	14.1627	11.9026	-4.4493
O	15.8015	11.0710	-2.4880
O	12.6513	11.8787	3.0824
O	14.2752	11.1056	5.0763
O	15.4437	5.0274	-3.1252
O	13.7474	4.2539	-5.0652
O	13.9022	5.0315	4.4167
O	12.2900	4.1645	2.4785
O	10.6838	4.2896	9.9487
O	22.0643	8.5487	3.0823
O	21.9601	4.2341	0.5377

O	22.0335	4.9836	3.0852
O	11.8647	11.0092	-5.5876
O	4.4277	11.7800	4.4400
O	22.8224	6.7611	4.9103
Si	18.9366	8.3038	-2.3163
Si	17.3423	8.3116	5.2599
Si	7.9908	1.5443	5.2675
Si	8.0037	15.1299	5.2212
Si	18.5398	8.2838	2.2815
Si	16.9895	8.2898	9.8464
Si	9.0749	1.5074	2.2147
Si	9.0679	15.1992	2.2723
Si	18.5244	5.2392	2.2759
Si	16.9676	5.2348	9.8241
Si	9.0820	12.0245	2.2625
Si	18.9238	5.2310	-2.3163
Si	17.3583	5.2424	5.2657
Si	9.5422	12.0072	-2.2331
Si	8.0291	11.9997	5.2063
Si	21.6736	8.3286	-0.7427
Si	20.1088	8.3351	6.7748
Si	12.2063	1.5193	-0.8050
Si	12.2398	15.1835	-0.7950
Si	10.6922	1.5217	6.7773
Si	10.7193	15.1428	6.7897
Si	15.8866	8.2867	0.6694
Si	14.2615	8.3410	8.2918
Si	15.8025	5.1623	0.7563
Si	14.2551	5.2412	8.2381
Si	6.3784	12.0136	0.7223
Si	20.0977	5.2108	6.7896
Si	12.2541	12.0199	-0.7502
Si	10.7203	12.0265	6.7882
Si	15.0212	8.2911	-4.6845
Si	11.9495	8.3162	10.3945
Si	14.5645	1.4544	-2.9072
Si	14.5887	15.1333	-2.8995
Si	13.0087	1.4586	4.6481
Si	13.0248	15.1471	4.6627
Si	14.5566	12.0588	-2.8690
Si	13.0044	12.0427	4.6622
Si	15.0033	5.2285	-4.6902
Si	13.5447	5.1449	2.8232
Si	11.9634	5.2154	10.3713
Si	16.1243	9.5357	-2.0709
Si	14.5486	9.5606	5.5611
Si	21.3500	9.5847	2.0378
Si	11.9663	2.6515	1.9989
Si	11.9887	16.3588	1.9904
Si	21.3343	3.9490	2.0264

Si	13.4837	10.8348	-5.5008
Si	11.9607	10.8582	1.9929
Si	10.3826	10.8145	9.5704
Si	16.1044	3.9890	-2.0543
Si	14.5541	3.9921	5.4852
Si	6.6904	10.7516	-2.0663
Si	5.1655	10.7471	5.4943
Si	10.6078	12.0294	-5.2918
Si	4.0502	12.0423	2.8606
Si	21.6675	5.2039	-0.7457
Si	22.4030	5.1990	4.6633
Si	22.4221	8.3265	4.6628
Si	19.7953	3.9688	9.5774
Si	19.8103	9.5712	9.5706
Si	10.3910	2.7149	9.5709
Si	13.4295	2.6971	-5.5100
H	19.4098	8.5047	-3.7003
H	6.8483	2.4770	5.4771
H	7.6152	0.1144	5.4642
H	6.8356	15.9497	5.5498
H	16.4509	8.5030	11.2037
H	7.9600	1.8765	1.3028
H	9.5328	0.0973	2.0347
H	7.9902	15.4883	1.3083
H	16.4153	5.0210	11.1808
H	19.3989	5.0322	-3.7018
H	9.1128	13.4122	-2.0000
H	22.6392	8.6367	-1.8203
H	10.8405	1.9116	-1.2538
H	12.3135	0.0773	-0.4619
H	10.8542	15.4696	-1.2285
H	10.7908	0.0783	7.0769
H	11.0035	15.9916	7.9593
H	6.3080	13.4572	0.4135
H	16.1807	8.5663	-5.5698
H	12.2883	8.5121	11.8158
H	14.9992	0.0566	-2.6754
H	14.2084	15.2183	-4.3346
H	15.7246	16.0285	-2.5370
H	13.4314	0.0625	4.8742
H	14.1620	16.0103	5.0553
H	16.1679	4.9661	-5.5748
H	12.3189	5.0027	11.7930
H	21.6028	10.9887	2.4146
H	12.2358	17.7629	2.3849
H	21.5928	2.5482	2.4104
H	14.0879	11.0692	-6.8239
H	8.9032	11.0352	9.5340
H	11.0663	11.6868	10.5706
H	6.1289	11.6798	-3.0990

H	6.3979	9.3208	-2.3711
H	4.6188	11.0068	6.8552
H	4.9604	9.3325	5.0474
H	9.4969	11.6715	-6.2172
H	11.0818	13.4285	-5.4557
H	2.8975	11.1758	2.4777
H	3.7516	13.4986	2.6862
H	22.6317	4.8982	-1.8247
H	23.5491	4.3478	5.0291
H	23.5619	9.1791	5.0342
H	20.3733	4.9250	10.5698
H	20.1043	2.5466	9.8996
H	20.3938	8.6184	10.5621
H	20.1088	10.9951	9.8944
H	8.9131	2.5082	9.4848
H	11.0270	1.8583	10.6130
H	11.9483	2.5112	-5.4377
H	13.9687	2.4757	-6.8835

	216 atoms	Al3-O8	
Energy =		-25743.45154484	Hartree
H	11.4112	8.6700	1.9441
Al	13.6087	8.2548	2.9258
O	13.7347	8.6710	4.5794
O	14.9150	8.8246	1.9655
O	12.1065	9.2454	2.3127
O	12.9907	6.6939	2.5520
O	20.1516	8.6059	-1.2670
O	17.7505	9.3828	-2.0196
O	18.3751	6.7759	-2.1305
O	18.5618	8.5635	6.2977
O	16.1662	9.3439	5.5737
O	17.8742	8.5227	3.7217
O	16.7894	6.7624	5.4075
O	9.1640	1.9319	6.3551
O	8.5461	1.7364	3.7424
O	9.1551	15.3616	6.3614
O	8.5762	15.5798	3.7679
O	7.5177	13.5593	5.1908
O	17.4178	8.5403	1.0903
O	19.7279	9.3568	2.0260
O	19.1221	6.7684	2.1369
O	15.8324	8.4933	8.7474
O	18.1796	9.3729	9.5766
O	17.6106	6.7521	9.7265
O	10.3420	2.5010	1.8950
O	10.3700	16.1019	1.8782
O	9.4913	13.6071	2.2025
O	17.3462	5.0057	1.1620
O	19.7076	4.1785	1.9731
O	17.9432	5.0078	3.7533
O	15.8136	5.0287	8.6981
O	18.1573	4.1403	9.5669
O	7.9133	11.6689	1.1974
O	10.3772	11.1393	1.8937
O	8.6205	11.5999	3.7679
O	20.1396	4.9354	-1.2706
O	17.7173	4.1853	-2.0085
O	18.5502	4.9713	6.3391
O	16.1812	4.1714	5.5285
O	10.7348	11.6278	-1.1877
O	8.3255	10.9473	-2.0022
O	10.0857	11.8509	-3.7621
O	9.1638	11.8003	6.3610
O	6.7823	11.0210	5.5707
O	21.9703	9.3010	0.5418
O	21.8360	6.7663	-0.2851
O	20.4191	9.2708	8.0645
O	21.1058	8.7426	5.5589

O	20.3344	6.7723	7.2023
O	12.6210	2.3935	0.5274
O	13.2412	1.8550	-2.0324
O	12.6425	16.0961	0.5034
O	13.2657	15.5421	-2.0202
O	12.3537	13.6088	-0.3816
O	11.0879	2.3692	8.1154
O	11.7113	1.8821	5.5529
O	11.6883	15.5643	5.5285
O	10.9789	13.5886	7.2058
O	15.4983	9.2863	-0.5779
O	15.5483	6.7981	0.3148
O	13.9729	9.3714	7.1100
O	13.3084	8.7038	9.5648
O	13.9999	6.7930	7.8143
O	15.4698	4.2986	-0.5686
O	14.8197	4.7951	1.9518
O	13.9439	4.2786	6.9605
O	13.2857	4.8190	9.4757
O	6.0314	11.1171	-0.5997
O	5.3361	11.6291	1.9140
O	20.4214	4.2777	8.0828
O	21.0931	4.7918	5.5689
O	12.6687	11.1619	0.5419
O	13.2651	11.6551	-1.9881
O	11.0349	11.1029	8.0843
O	11.6801	11.6271	5.5376
O	15.4869	8.4716	-3.1075
O	13.7902	9.3046	-5.0035
O	14.4913	6.7705	-4.8904
O	10.6822	9.2395	9.9597
O	11.5203	6.7613	10.1111
O	14.2084	1.6529	-4.4946
O	15.8026	2.4479	-2.4673
O	15.0504	13.5796	-2.5753
O	12.5981	1.5912	3.0639
O	14.2556	2.4510	5.0332
O	12.6707	15.3515	3.0627
O	13.4037	13.5943	4.8885
O	14.1384	11.9274	-4.4571
O	15.8091	11.0656	-2.5333
O	12.7547	11.6793	3.1436
O	14.3310	11.1921	5.2486
O	15.4327	5.0199	-3.1251
O	13.7432	4.2622	-5.0750
O	13.8930	5.0324	4.4150
O	12.2671	4.1814	2.4703
O	10.6848	4.2884	9.9484
O	22.0635	8.5536	3.0826
O	21.9670	4.2333	0.5362

O	22.0239	4.9796	3.0863
O	11.8635	11.0008	-5.5930
O	4.4332	11.7806	4.4392
O	22.8130	6.7616	4.9112
Si	18.9399	8.3096	-2.3141
Si	17.3522	8.2947	5.2417
Si	7.9918	1.5441	5.2675
Si	8.0011	15.1254	5.2288
Si	18.5252	8.2924	2.2507
Si	17.0046	8.2800	9.8554
Si	9.0755	1.5074	2.2142
Si	9.0746	15.1957	2.2643
Si	18.5216	5.2486	2.2573
Si	16.9756	5.2329	9.8285
Si	9.0708	12.0347	2.2751
Si	18.9235	5.2352	-2.3152
Si	17.3698	5.2375	5.2512
Si	9.5427	12.0087	-2.2425
Si	8.0191	12.0034	5.2274
Si	21.6760	8.3306	-0.7407
Si	20.1001	8.3306	6.7734
Si	12.2076	1.5201	-0.8057
Si	12.2426	15.1891	-0.8030
Si	10.6939	1.5211	6.7759
Si	10.7214	15.1481	6.7873
Si	15.8305	8.3606	0.7315
Si	14.2754	8.3266	8.3078
Si	15.7912	5.2402	0.7238
Si	14.2621	5.2365	8.2360
Si	6.3804	12.0123	0.7223
Si	20.0968	5.2105	6.7892
Si	12.2579	12.0347	-0.7805
Si	10.7205	12.0278	6.7938
Si	15.0332	8.2918	-4.6671
Si	11.9571	8.3084	10.3964
Si	14.5698	1.4589	-2.9033
Si	14.5946	15.1298	-2.9035
Si	13.0103	1.4608	4.6458
Si	13.0165	15.1599	4.6509
Si	14.5721	12.0544	-2.8849
Si	13.0451	12.0202	4.7282
Si	15.0033	5.2310	-4.6886
Si	13.4933	5.1944	2.8334
Si	11.9685	5.2105	10.3685
Si	16.1315	9.5366	-2.0504
Si	14.5539	9.5981	5.5987
Si	21.3455	9.5852	2.0322
Si	11.9636	2.6618	1.9967
Si	11.9861	16.3679	1.9792
Si	21.3268	3.9491	2.0201

Si	13.4874	10.8327	-5.5000
Si	11.9710	10.9094	1.9754
Si	10.3849	10.8095	9.5747
Si	16.0987	3.9961	-2.0403
Si	14.5622	3.9938	5.4743
Si	6.6927	10.7509	-2.0695
Si	5.1624	10.7444	5.4960
Si	10.6148	12.0258	-5.2994
Si	4.0507	12.0425	2.8615
Si	21.6670	5.2041	-0.7458
Si	22.3987	5.1981	4.6617
Si	22.4150	8.3292	4.6615
Si	19.7967	3.9677	9.5772
Si	19.8138	9.5708	9.5687
Si	10.3916	2.7140	9.5702
Si	13.4311	2.7019	-5.5092
H	19.4098	8.5047	-3.7003
H	6.8483	2.4770	5.4771
H	7.6152	0.1144	5.4642
H	6.8356	15.9497	5.5498
H	16.4509	8.5030	11.2037
H	7.9600	1.8765	1.3028
H	9.5328	0.0973	2.0347
H	7.9902	15.4883	1.3083
H	16.4153	5.0210	11.1808
H	19.3989	5.0322	-3.7018
H	9.1128	13.4122	-2.0000
H	22.6392	8.6367	-1.8203
H	10.8405	1.9116	-1.2538
H	12.3135	0.0773	-0.4619
H	10.8542	15.4696	-1.2285
H	10.7908	0.0783	7.0769
H	11.0035	15.9916	7.9593
H	6.3080	13.4572	0.4135
H	16.1807	8.5663	-5.5698
H	12.2883	8.5121	11.8158
H	14.9992	0.0566	-2.6754
H	14.2084	15.2183	-4.3346
H	15.7246	16.0285	-2.5370
H	13.4314	0.0625	4.8742
H	14.1620	16.0103	5.0553
H	16.1679	4.9661	-5.5748
H	12.3189	5.0027	11.7930
H	21.6028	10.9887	2.4146
H	12.2358	17.7629	2.3849
H	21.5928	2.5482	2.4104
H	14.0879	11.0692	-6.8239
H	8.9032	11.0352	9.5340
H	11.0663	11.6868	10.5706
H	6.1289	11.6798	-3.0990

H	6.3979	9.3208	-2.3711
H	4.6188	11.0068	6.8552
H	4.9604	9.3325	5.0474
H	9.4969	11.6715	-6.2172
H	11.0818	13.4285	-5.4557
H	2.8975	11.1758	2.4777
H	3.7516	13.4986	2.6862
H	22.6317	4.8982	-1.8247
H	23.5491	4.3478	5.0291
H	23.5619	9.1791	5.0342
H	20.3733	4.9250	10.5698
H	20.1043	2.5466	9.8996
H	20.3938	8.6184	10.5621
H	20.1088	10.9951	9.8944
H	8.9131	2.5082	9.4848
H	11.0270	1.8583	10.6130
H	11.9483	2.5112	-5.4377
H	13.9687	2.4757	-6.8835

	216 atoms	Al3-O10	
Energy =		-25743.44838877	Hartree
H	11.9830	6.7022	2.2419
Al	13.5489	8.4297	2.9154
O	13.8717	8.4702	4.5989
O	14.9586	8.5887	1.9599
O	12.1522	9.2822	2.3785
O	12.8904	6.6812	2.5991
O	20.1504	8.5991	-1.2666
O	17.7508	9.3866	-2.0201
O	18.3721	6.7764	-2.1328
O	18.5745	8.5596	6.2879
O	16.1878	9.3757	5.6021
O	17.8598	8.5406	3.7127
O	16.7972	6.7755	5.4030
O	9.1656	1.9338	6.3538
O	8.5478	1.7355	3.7467
O	9.1561	15.3662	6.3600
O	8.5704	15.5807	3.7662
O	7.5228	13.5565	5.1967
O	17.4488	8.5117	1.0635
O	19.7346	9.3511	2.0314
O	19.1263	6.7628	2.1688
O	15.8219	8.5182	8.7477
O	18.1801	9.3720	9.5724
O	17.5927	6.7572	9.7123
O	10.3380	2.5075	1.9097
O	10.3725	16.0958	1.8911
O	9.4774	13.6017	2.1992
O	17.3623	4.9929	1.1658
O	19.7122	4.1724	1.9885
O	17.9207	4.9887	3.7534
O	15.8060	5.0192	8.6957
O	18.1565	4.1466	9.5603
O	7.8989	11.6669	1.2024
O	10.3615	11.1302	1.8392
O	8.6310	11.5989	3.7631
O	20.1401	4.9404	-1.2685
O	17.7265	4.1815	-2.0082
O	18.5579	4.9806	6.3387
O	16.1850	4.1877	5.5463
O	10.7392	11.6253	-1.1979
O	8.3235	10.9480	-2.0044
O	10.0738	11.8506	-3.7657
O	9.1616	11.7898	6.3629
O	6.7840	11.0212	5.5644
O	21.9707	9.3028	0.5399
O	21.8394	6.7674	-0.2815
O	20.4228	9.2721	8.0648
O	21.1128	8.7422	5.5575

O	20.3460	6.7745	7.2065
O	12.6179	2.3868	0.5236
O	13.2546	1.8603	-2.0308
O	12.6419	16.0868	0.5112
O	13.2634	15.5541	-2.0160
O	12.3594	13.6032	-0.3956
O	11.0879	2.3721	8.1189
O	11.7126	1.8861	5.5595
O	11.6961	15.5430	5.5363
O	10.9624	13.5836	7.2244
O	15.5127	9.3064	-0.5674
O	15.6419	6.7736	0.1358
O	13.9662	9.3811	7.0849
O	13.2946	8.7228	9.5525
O	14.0020	6.8091	7.8206
O	15.4885	4.2288	-0.5647
O	14.8253	4.9306	1.9011
O	13.9241	4.3084	6.9558
O	13.2783	4.8226	9.4744
O	6.0224	11.1198	-0.6027
O	5.3248	11.6258	1.9072
O	20.4236	4.2761	8.0818
O	21.0946	4.7942	5.5659
O	12.6721	11.1476	0.5470
O	13.2641	11.6473	-1.9833
O	11.0347	11.0944	8.0835
O	11.6873	11.6280	5.5495
O	15.4861	8.4844	-3.1020
O	13.7883	9.3032	-5.0096
O	14.4974	6.7720	-4.8726
O	10.6711	9.2424	9.9681
O	11.5228	6.7681	10.1097
O	14.1982	1.6432	-4.5017
O	15.8210	2.4288	-2.4946
O	15.0443	13.5849	-2.5746
O	12.6147	1.6228	3.0743
O	14.2564	2.4660	5.0396
O	12.6794	15.3458	3.0741
O	13.4351	13.5811	4.9256
O	14.1418	11.9236	-4.4542
O	15.8145	11.0814	-2.5326
O	12.6312	11.8401	3.0889
O	14.2657	11.0967	5.0882
O	15.4334	5.0060	-3.1182
O	13.7474	4.2594	-5.0670
O	13.9152	5.0303	4.3968
O	12.2741	4.1957	2.4218
O	10.6820	4.2929	9.9499
O	22.0714	8.5518	3.0793
O	21.9642	4.2323	0.5351

O	22.0309	4.9804	3.0847
O	11.8619	11.0020	-5.5926
O	4.4284	11.7795	4.4398
O	22.8182	6.7617	4.9105
Si	18.9365	8.3132	-2.3152
Si	17.3528	8.3156	5.2402
Si	7.9888	1.5436	5.2726
Si	8.0017	15.1239	5.2266
Si	18.5293	8.2918	2.2540
Si	16.9961	8.2897	9.8502
Si	9.0672	1.5042	2.2195
Si	9.0733	15.1857	2.2640
Si	18.5311	5.2442	2.2723
Si	16.9713	5.2354	9.8254
Si	9.0861	12.0165	2.2593
Si	18.9279	5.2408	-2.3167
Si	17.3783	5.2552	5.2571
Si	9.5444	12.0085	-2.2373
Si	8.0272	11.9998	5.2160
Si	21.6757	8.3316	-0.7414
Si	20.1085	8.3326	6.7730
Si	12.2113	1.5155	-0.8207
Si	12.2407	15.1819	-0.7979
Si	10.6911	1.5226	6.7850
Si	10.7195	15.1422	6.7905
Si	15.8699	8.3148	0.6799
Si	14.2666	8.3539	8.2983
Si	15.8444	5.2511	0.6501
Si	14.2564	5.2568	8.2454
Si	6.3782	12.0127	0.7222
Si	20.1062	5.2153	6.7914
Si	12.2591	12.0167	-0.7566
Si	10.7179	12.0255	6.7914
Si	15.0327	8.3015	-4.6622
Si	11.9508	8.3209	10.3952
Si	14.5745	1.4531	-2.9176
Si	14.5871	15.1359	-2.8994
Si	13.0133	1.4663	4.6619
Si	13.0278	15.1409	4.6597
Si	14.5601	12.0611	-2.8760
Si	13.0028	12.0305	4.6611
Si	15.0060	5.2339	-4.6840
Si	13.5338	5.1226	2.8350
Si	11.9637	5.2197	10.3718
Si	16.1310	9.5574	-2.0512
Si	14.5584	9.5561	5.5665
Si	21.3509	9.5855	2.0321
Si	11.9557	2.6446	1.9847
Si	11.9885	16.3586	1.9896
Si	21.3336	3.9485	2.0217

Si	13.4844	10.8376	-5.4986
Si	11.9544	10.8310	1.9858
Si	10.3826	10.8153	9.5708
Si	16.1134	3.9728	-2.0561
Si	14.5767	3.9997	5.4998
Si	6.6924	10.7527	-2.0670
Si	5.1659	10.7466	5.4939
Si	10.6107	12.0273	-5.2954
Si	4.0499	12.0426	2.8604
Si	21.6690	5.2072	-0.7454
Si	22.4028	5.2001	4.6609
Si	22.4200	8.3292	4.6588
Si	19.7963	3.9687	9.5764
Si	19.8142	9.5715	9.5684
Si	10.3901	2.7192	9.5762
Si	13.4291	2.7048	-5.5126
H	19.4098	8.5047	-3.7003
H	6.8483	2.4770	5.4771
H	7.6152	0.1144	5.4642
H	6.8356	15.9497	5.5498
H	16.4509	8.5030	11.2037
H	7.9600	1.8765	1.3028
H	9.5328	0.0973	2.0347
H	7.9902	15.4883	1.3083
H	16.4153	5.0210	11.1808
H	19.3989	5.0322	-3.7018
H	9.1128	13.4122	-2.0000
H	22.6392	8.6367	-1.8203
H	10.8405	1.9116	-1.2538
H	12.3135	0.0773	-0.4619
H	10.8542	15.4696	-1.2285
H	10.7908	0.0783	7.0769
H	11.0035	15.9916	7.9593
H	6.3080	13.4572	0.4135
H	16.1807	8.5663	-5.5698
H	12.2883	8.5121	11.8158
H	14.9992	0.0566	-2.6754
H	14.2084	15.2183	-4.3346
H	15.7246	16.0285	-2.5370
H	13.4314	0.0625	4.8742
H	14.1620	16.0103	5.0553
H	16.1679	4.9661	-5.5748
H	12.3189	5.0027	11.7930
H	21.6028	10.9887	2.4146
H	12.2358	17.7629	2.3849
H	21.5928	2.5482	2.4104
H	14.0879	11.0692	-6.8239
H	8.9032	11.0352	9.5340
H	11.0663	11.6868	10.5706
H	6.1289	11.6798	-3.0990

H	6.3979	9.3208	-2.3711
H	4.6188	11.0068	6.8552
H	4.9604	9.3325	5.0474
H	9.4969	11.6715	-6.2172
H	11.0818	13.4285	-5.4557
H	2.8975	11.1758	2.4777
H	3.7516	13.4986	2.6862
H	22.6317	4.8982	-1.8247
H	23.5491	4.3478	5.0291
H	23.5619	9.1791	5.0342
H	20.3733	4.9250	10.5698
H	20.1043	2.5466	9.8996
H	20.3938	8.6184	10.5621
H	20.1088	10.9951	9.8944
H	8.9131	2.5082	9.4848
H	11.0270	1.8583	10.6130
H	11.9483	2.5112	-5.4377
H	13.9687	2.4757	-6.8835

	210 atoms	Al4-O2		
Energy =		-24863.53354741	Hartree	
H	16.5874	10.3122	5.7049	
Al	14.3284	9.5139	5.4931	
O	16.2476	9.4127	5.5334	
O	13.8382	9.2763	7.1148	
O	13.8420	8.3244	4.3620	
O	14.2480	11.1526	4.9638	
O	20.1588	8.6973	-1.3009	
O	17.6928	9.2958	-2.0390	
O	18.5961	8.6309	6.3490	
O	18.0034	8.5086	3.7507	
O	16.8014	6.8159	5.4516	
O	14.6262	9.2870	13.0301	
O	16.4187	8.4380	11.2570	
O	15.4201	6.7486	13.0774	
O	9.1410	15.3439	6.3722	
O	8.5999	15.4889	3.7668	
O	7.4711	13.5510	5.2496	
O	17.2830	8.6435	1.1935	
O	19.7012	9.3532	1.9285	
O	19.0297	6.7754	1.9990	
O	15.8356	8.4971	8.6564	
O	18.1810	9.2926	9.4793	
O	17.5796	6.6969	9.5956	
O	10.3451	16.0932	1.8676	
O	9.5141	13.5593	2.1348	
O	17.3048	4.8750	1.1928	
O	19.7102	4.2020	1.9743	
O	17.9665	5.1083	3.7633	
O	15.8033	4.9174	8.6441	
O	18.1507	4.1017	9.5355	
O	16.3349	4.9884	11.2423	
O	10.3223	11.0326	1.8256	
O	8.6071	11.6212	3.7667	
O	8.7971	10.9574	9.4829	
O	7.9932	13.5377	9.5388	
O	18.5327	4.9775	6.3405	
O	16.1382	4.2424	5.4827	
O	9.1524	11.7862	6.3701	
O	20.4725	9.2534	8.1091	
O	21.1043	8.7256	5.5713	
O	20.3127	6.7641	7.2138	
O	12.6250	16.0609	0.5145	
O	13.2705	15.5146	-1.9923	
O	12.2961	13.5736	-0.3848	
O	11.0274	2.3638	8.0815	
O	11.7474	1.8732	5.5290	
O	11.0574	16.0676	8.0582	
O	11.6515	15.5938	5.5208	

O	10.9857	13.5788	7.1627
O	15.4333	9.2536	-0.5945
O	14.7368	8.6693	1.9010
O	15.6400	6.7546	0.2549
O	13.3867	8.6311	9.6562
O	14.0052	6.7409	7.8569
O	14.7501	4.8277	1.9251
O	13.9013	4.2670	6.9263
O	13.2766	4.7560	9.4774
O	20.4155	4.2646	8.0748
O	21.0811	4.8089	5.5581
O	12.6215	11.0898	0.5066
O	13.2613	11.6663	-2.0123
O	11.0620	11.0944	8.0538
O	11.6646	11.6042	5.5024
O	12.2135	9.2893	2.4300
O	12.9488	6.7412	2.4527
O	12.2508	8.4269	12.0068
O	10.7692	9.2544	9.9319
O	11.5246	6.7458	10.0730
O	21.9609	8.4914	3.0805
O	23.6461	9.2687	5.0126
O	22.8155	6.7619	4.9030
O	12.6264	1.6112	3.0239
O	14.2669	2.4878	4.9670
O	12.6226	15.3286	3.0624
O	14.2479	16.0707	5.0601
O	13.3561	13.6105	4.9214
O	22.0293	4.9786	3.0777
O	15.8297	11.0835	-2.4918
O	15.0663	13.5876	-2.6293
O	12.5448	11.9068	3.0331
O	11.0492	11.8397	10.6063
O	12.7225	11.0306	12.5392
O	12.0365	13.5591	12.3835
O	13.8344	5.0825	4.3897
O	12.1892	4.2344	2.4648
O	10.6803	4.2722	9.9474
O	23.3005	11.8784	4.3975
O	21.6016	11.0829	2.4473
O	22.6013	11.6844	9.4752
O	20.0223	11.1241	9.9526
O	21.9881	9.2435	0.5492
O	12.2259	17.8917	2.4174
O	8.8106	16.1136	9.5247
O	11.1455	15.3742	10.6292
O	13.9345	18.6750	4.3980
O	23.3200	11.0967	6.9404
O	21.7122	6.7443	-0.3473
O	21.9566	4.2269	0.5149

Si	18.9402	8.2763	-2.3162
Si	17.4707	8.2735	5.2474
Si	15.8948	8.2943	12.7913
Si	7.9927	15.1069	5.2329
Si	18.4990	8.3080	2.2032
Si	16.9899	8.2233	9.7579
Si	9.0786	15.1372	2.2472
Si	18.4957	5.2425	2.2341
Si	16.9575	5.1835	9.7588
Si	9.0648	11.9821	2.2422
Si	7.5382	11.9697	9.7804
Si	17.3582	5.2680	5.2546
Si	15.8472	5.1838	12.7918
Si	7.9967	11.9944	5.2338
Si	20.1424	8.3323	6.8171
Si	12.2235	15.1557	-0.7932
Si	10.6734	1.5182	6.7160
Si	10.7093	15.1348	6.7704
Si	15.7568	8.3255	0.6976
Si	14.2448	8.2977	8.3058
Si	15.7736	5.1865	0.7010
Si	14.2418	5.1798	8.2338
Si	20.0867	5.2015	6.7887
Si	12.2184	11.9918	-0.7984
Si	10.7215	12.0194	6.7597
Si	13.4312	8.2662	2.8126
Si	11.9815	8.2728	10.4000
Si	22.3835	8.3091	4.6415
Si	13.0352	1.4636	4.6054
Si	12.9646	15.1552	4.6454
Si	22.3987	5.1968	4.6548
Si	14.5771	12.0501	-2.9017
Si	12.9700	12.0604	4.6071
Si	11.4846	12.0358	12.1715
Si	13.4299	5.2309	2.8118
Si	11.9640	5.1967	10.3620
Si	22.8628	12.0806	2.8193
Si	21.2965	12.0743	10.3948
Si	16.0788	9.5257	-2.0769
Si	13.0043	9.4874	13.0082
Si	21.3182	9.5414	2.0052
Si	19.7878	9.5506	9.5778
Si	11.8993	2.6817	1.9954
Si	11.9534	16.3431	1.9771
Si	10.3825	2.7013	9.5591
Si	10.4287	16.3622	9.5424
Si	19.7896	3.9604	9.5752
Si	11.9383	10.8303	1.9694
Si	10.4190	10.7946	9.5208
Si	14.5164	4.0296	5.4346

Si	14.5956	17.6262	5.4885
Si	13.5072	18.8589	2.8145
Si	11.5129	15.1080	12.2076
Si	7.5408	15.1052	9.7823
Si	23.9629	10.8171	5.4605
Si	23.6612	12.0122	8.2704
Si	21.6557	8.3233	-0.7642
Si	21.3310	3.9534	2.0046
Si	14.5848	15.1395	-2.9031
Si	21.5952	5.1669	-0.7927
H	19.4026	8.4671	-3.7162
H	18.5240	6.8662	-2.0429
H	17.0004	8.6838	13.6942
H	6.8545	15.9873	5.5276
H	7.9801	15.4407	1.3089
H	7.9416	11.6891	1.3278
H	6.4357	11.6334	8.8286
H	7.1212	11.8012	11.2083
H	16.9769	4.8216	13.6967
H	14.6467	4.3323	12.9885
H	6.8562	11.1049	5.5272
H	10.8538	15.4747	-1.2453
H	9.3221	1.9136	6.2326
H	10.7752	0.0684	7.0319
H	15.4877	4.3481	-0.4740
H	10.8617	11.6401	-1.2706
H	13.4816	0.0682	4.8450
H	23.5472	4.3421	5.0264
H	14.2215	11.8386	-4.3223
H	10.3164	11.7700	13.0576
H	12.3155	5.0252	11.7876
H	24.0145	11.7154	1.9477
H	22.4014	13.4883	2.6334
H	21.6505	11.7829	11.8149
H	20.9011	13.4998	10.1836
H	15.4490	8.6179	-3.0621
H	12.4585	9.2566	14.3484
H	20.3839	8.6653	10.6022
H	10.4208	2.4807	2.0229
H	12.4846	2.4812	0.6394
H	8.9003	2.5096	9.5179
H	11.0383	1.8353	10.5822
H	10.7170	17.7683	9.8865
H	20.3486	4.9399	10.5611
H	20.1197	2.5491	9.9166
H	16.0896	17.7575	5.4838
H	14.0002	17.9047	6.8310
H	14.6628	18.4837	1.9437
H	13.0621	20.2684	2.6304
H	10.2841	15.2925	13.0396

H	12.6213	16.0184	12.6148
H	6.4557	15.4559	8.8139
H	7.1035	15.2788	11.2020
H	25.4349	10.9777	5.4930
H	25.0138	11.6409	8.7658
H	23.5558	13.4494	7.8944
H	22.6678	8.6178	-1.7957
H	21.5847	2.5494	2.3947
H	14.1810	15.2692	-4.3312
H	15.7161	16.0360	-2.5354
H	20.1939	4.8825	-1.2351
H	22.5949	4.8982	-1.8704

	210 atoms	Al4-O3		
Energy =		-24863.53975420	Hartree	
H	12.9591	9.8817	7.3143	
Al	14.5434	9.5410	5.3816	
O	16.1597	9.1471	5.7522	
O	13.7540	9.3406	7.1110	
O	13.6820	8.4332	4.3855	
O	14.2617	11.1933	5.0231	
O	20.1643	8.6992	-1.3082	
O	17.6921	9.2943	-2.0401	
O	18.5761	8.4158	6.3914	
O	17.9561	8.7507	3.8170	
O	16.9389	6.6924	5.1575	
O	14.6280	9.2921	13.0436	
O	16.3817	8.4358	11.2555	
O	15.4118	6.7488	13.0895	
O	9.1323	15.3306	6.3874	
O	8.6038	15.4813	3.7731	
O	7.4768	13.5444	5.2624	
O	17.2916	8.5368	1.2299	
O	19.7052	9.2771	1.9215	
O	18.9485	6.7517	2.3366	
O	15.7833	8.5973	8.6991	
O	18.1691	9.3307	9.5128	
O	17.5371	6.7389	9.5582	
O	10.3372	16.1043	1.8672	
O	9.5157	13.5629	2.1348	
O	17.3151	4.9535	1.1835	
O	19.7478	4.2522	1.8442	
O	18.0046	4.6828	3.7545	
O	15.7926	4.9459	8.6449	
O	18.1594	4.1417	9.5240	
O	16.3404	5.0213	11.2361	
O	10.3157	11.0359	1.8064	
O	8.6258	11.6269	3.7717	
O	8.7998	10.9934	9.4454	
O	7.9909	13.5488	9.5408	
O	18.5704	4.9435	6.3492	
O	16.1605	4.1968	5.5693	
O	9.1521	11.7628	6.3818	
O	20.4455	9.2064	8.1023	
O	21.0856	8.6953	5.5744	
O	20.4173	6.7049	7.2406	
O	12.6192	16.0697	0.5106	
O	13.2735	15.5153	-1.9934	
O	12.2914	13.5809	-0.3832	
O	11.0285	2.3626	8.0789	
O	11.7437	1.8720	5.5247	
O	11.0570	16.0533	8.0648	
O	11.6335	15.5847	5.5244	

O	10.9741	13.5623	7.1807
O	15.4579	9.2516	-0.5586
O	14.7436	8.6788	1.9521
O	15.5478	6.7532	0.2665
O	13.2751	8.6857	9.5867
O	14.0270	6.7859	7.8095
O	14.7611	4.7879	1.9265
O	13.8823	4.2906	6.9214
O	13.2717	4.8361	9.4634
O	20.4423	4.2133	8.0717
O	21.0958	4.7606	5.5394
O	12.6280	11.1063	0.4995
O	13.2492	11.6792	-2.0255
O	11.1013	11.0469	8.0481
O	11.6832	11.5640	5.5379
O	12.1705	9.2396	2.3292
O	12.9763	6.7264	2.4791
O	12.2696	8.4274	12.0023
O	10.7086	9.2598	9.9672
O	11.4834	6.7518	10.0900
O	21.9833	8.4977	3.0870
O	23.6394	9.2532	5.0424
O	22.8109	6.7495	4.8998
O	12.6296	1.6071	3.0248
O	14.2653	2.4791	4.9891
O	12.6088	15.3417	3.0603
O	14.2308	16.0636	5.0693
O	13.3020	13.6044	4.9058
O	22.0379	4.9725	3.0607
O	15.8167	11.0733	-2.4715
O	15.0698	13.5852	-2.6306
O	12.5384	11.8355	3.0492
O	11.0567	11.8450	10.5980
O	12.7193	11.0287	12.5292
O	12.0365	13.5609	12.3825
O	13.8836	5.0744	4.3957
O	12.2025	4.2273	2.4909
O	10.6849	4.2677	9.9420
O	23.2922	11.8571	4.3896
O	21.5858	11.0760	2.4342
O	22.6146	11.6888	9.4832
O	20.0389	11.1180	9.9366
O	21.9899	9.2386	0.5519
O	12.2196	17.9022	2.4113
O	8.8081	16.1238	9.5272
O	11.1382	15.3708	10.6316
O	13.9302	18.6656	4.3965
O	23.3198	11.1018	6.9408
O	21.7031	6.7403	-0.3431
O	22.0123	4.2118	0.4931

Si	18.9424	8.2763	-2.3149
Si	17.3972	8.2650	5.2739
Si	15.8928	8.2910	12.7994
Si	7.9920	15.1035	5.2364
Si	18.4631	8.3187	2.3364
Si	16.9840	8.2672	9.7616
Si	9.0790	15.1402	2.2473
Si	18.4935	5.1822	2.2844
Si	16.9619	5.2109	9.7477
Si	9.0678	11.9855	2.2369
Si	7.5302	11.9863	9.7775
Si	17.4274	5.1445	5.2038
Si	15.8495	5.1865	12.7888
Si	8.0008	11.9891	5.2334
Si	20.1294	8.2571	6.8158
Si	12.2243	15.1603	-0.7973
Si	10.6739	1.5160	6.7103
Si	10.7025	15.1249	6.7767
Si	15.7545	8.3090	0.7362
Si	14.2441	8.2917	8.3520
Si	15.7711	5.1929	0.7010
Si	14.2460	5.2012	8.2107
Si	20.1264	5.1643	6.7855
Si	12.2170	12.0013	-0.8055
Si	10.7175	12.0099	6.7608
Si	13.4017	8.2722	2.8140
Si	11.9211	8.2755	10.4194
Si	22.3741	8.2994	4.6532
Si	13.0373	1.4621	4.6055
Si	12.9477	15.1562	4.6405
Si	22.4029	5.1864	4.6397
Si	14.5794	12.0463	-2.9014
Si	12.9728	12.0405	4.6093
Si	11.4846	12.0415	12.1712
Si	13.4587	5.2148	2.8218
Si	11.9521	5.2026	10.3643
Si	22.8496	12.0695	2.8127
Si	21.3028	12.0738	10.3912
Si	16.0774	9.5144	-2.0552
Si	13.0112	9.4936	13.0145
Si	21.3094	9.5239	2.0090
Si	19.7931	9.5459	9.5651
Si	11.9033	2.6832	1.9993
Si	11.9467	16.3550	1.9700
Si	10.3826	2.6940	9.5541
Si	10.4277	16.3635	9.5441
Si	19.8012	3.9598	9.5677
Si	11.9291	10.8019	1.9429
Si	10.4090	10.8039	9.5248
Si	14.5488	4.0182	5.4455

Si	14.5942	17.6199	5.4886
Si	13.5071	18.8597	2.8131
Si	11.5118	15.1121	12.2085
Si	7.5388	15.1173	9.7834
Si	23.9591	10.8074	5.4609
Si	23.6644	12.0135	8.2681
Si	21.6568	8.3211	-0.7607
Si	21.3526	3.9546	1.9731
Si	14.5884	15.1363	-2.9044
Si	21.6002	5.1623	-0.7938
H	19.4026	8.4671	-3.7162
H	18.5240	6.8662	-2.0429
H	17.0004	8.6838	13.6942
H	6.8545	15.9873	5.5276
H	7.9801	15.4407	1.3089
H	7.9416	11.6891	1.3278
H	6.4357	11.6334	8.8286
H	7.1212	11.8012	11.2083
H	16.9769	4.8216	13.6967
H	14.6467	4.3323	12.9885
H	6.8562	11.1049	5.5272
H	10.8538	15.4747	-1.2453
H	9.3221	1.9136	6.2326
H	10.7752	0.0684	7.0319
H	15.4877	4.3481	-0.4740
H	10.8617	11.6401	-1.2706
H	13.4816	0.0682	4.8450
H	23.5472	4.3421	5.0264
H	14.2215	11.8386	-4.3223
H	10.3164	11.7700	13.0576
H	12.3155	5.0252	11.7876
H	24.0145	11.7154	1.9477
H	22.4014	13.4883	2.6334
H	21.6505	11.7829	11.8149
H	20.9011	13.4998	10.1836
H	15.4490	8.6179	-3.0621
H	12.4585	9.2566	14.3484
H	20.3839	8.6653	10.6022
H	10.4208	2.4807	2.0229
H	12.4846	2.4812	0.6394
H	8.9003	2.5096	9.5179
H	11.0383	1.8353	10.5822
H	10.7170	17.7683	9.8865
H	20.3486	4.9399	10.5611
H	20.1197	2.5491	9.9166
H	16.0896	17.7575	5.4838
H	14.0002	17.9047	6.8310
H	14.6628	18.4837	1.9437
H	13.0621	20.2684	2.6304
H	10.2841	15.2925	13.0396

H	12.6213	16.0184	12.6148
H	6.4557	15.4559	8.8139
H	7.1035	15.2788	11.2020
H	25.4349	10.9777	5.4930
H	25.0138	11.6409	8.7658
H	23.5558	13.4494	7.8944
H	22.6678	8.6178	-1.7957
H	21.5847	2.5494	2.3947
H	14.1810	15.2692	-4.3312
H	15.7161	16.0360	-2.5354
H	20.1939	4.8825	-1.2351
H	22.5949	4.8982	-1.8704

	210 atoms	Al4-O5	
Energy =	-24863.54099989	Hartree	
H	13.8435	7.3703	4.8098
Al	14.6264	9.6445	5.5133
O	16.3139	9.3886	5.3808
O	13.9011	9.2981	7.0346
O	13.8347	8.2715	4.4138
O	14.1395	11.0673	4.7091
O	20.1684	8.6979	-1.3002
O	17.6955	9.2863	-2.0262
O	18.5756	8.4922	6.4238
O	18.2180	8.3631	3.8176
O	16.7911	6.7799	5.3996
O	14.6367	9.2989	13.0624
O	16.3847	8.4509	11.2510
O	15.4188	6.7509	13.0765
O	9.1414	15.3394	6.3702
O	8.6007	15.4784	3.7642
O	7.4664	13.5502	5.2454
O	17.2599	8.6914	1.3233
O	19.7253	9.3147	1.8729
O	18.9894	6.7395	1.8726
O	15.8349	8.4675	8.6568
O	18.1743	9.3125	9.5068
O	17.6014	6.7122	9.6306
O	10.3528	16.0780	1.8702
O	9.5140	13.5477	2.1277
O	17.2733	4.7825	1.2447
O	19.7228	4.1798	1.9140
O	18.0617	5.0848	3.7665
O	15.8142	4.9765	8.6375
O	18.1501	4.1104	9.5347
O	16.3250	4.9861	11.2376
O	10.2739	10.9860	1.7936
O	8.6183	11.6172	3.7759
O	8.7954	10.9616	9.4829
O	7.9918	13.5396	9.5382
O	18.5476	4.9801	6.3574
O	16.1793	4.2140	5.4183
O	9.1404	11.7903	6.3851
O	20.4719	9.2262	8.1182
O	21.0874	8.7133	5.5830
O	20.3994	6.7294	7.2368
O	12.6296	16.0492	0.5193
O	13.2703	15.4997	-1.9898
O	12.2933	13.5645	-0.3762
O	11.0283	2.3631	8.0802
O	11.7487	1.8739	5.5291
O	11.0554	16.0715	8.0573
O	11.6534	15.5760	5.5231

O	10.9778	13.5772	7.1822
O	15.4538	9.2352	-0.5668
O	14.7051	8.6435	1.9124
O	15.7000	6.7545	0.3028
O	13.3541	8.6488	9.5661
O	13.9946	6.7636	7.7657
O	14.6990	4.8559	1.8996
O	13.9450	4.2778	6.9066
O	13.2838	4.8011	9.4486
O	20.4220	4.2290	8.0732
O	21.0839	4.7732	5.5524
O	12.6003	11.0666	0.5099
O	13.2656	11.6575	-2.0043
O	11.0589	11.0869	8.0562
O	11.6401	11.6120	5.5020
O	12.2300	9.3669	2.5431
O	12.8777	6.7487	2.5202
O	12.3071	8.4330	11.9750
O	10.7577	9.2587	9.9485
O	11.5216	6.7545	10.0734
O	21.9640	8.4922	3.0888
O	23.6359	9.2611	5.0271
O	22.8027	6.7549	4.9045
O	12.6017	1.5651	3.0119
O	14.2282	2.5504	4.8838
O	12.6338	15.3267	3.0712
O	14.2520	16.0578	5.0760
O	13.3560	13.5897	4.9263
O	22.0200	4.9778	3.0728
O	15.8239	11.0716	-2.4744
O	15.0683	13.5816	-2.6306
O	12.3912	12.0304	2.9962
O	11.0501	11.8446	10.6077
O	12.7235	11.0336	12.5430
O	12.0366	13.5609	12.3862
O	13.8768	5.1899	4.4082
O	12.1914	4.2034	2.5356
O	10.6967	4.2735	9.9380
O	23.2993	11.8689	4.3947
O	21.6012	11.0800	2.4364
O	22.6089	11.6881	9.4818
O	20.0291	11.1228	9.9475
O	22.0217	9.2381	0.5493
O	12.2258	17.8877	2.4125
O	8.8100	16.1152	9.5236
O	11.1435	15.3732	10.6291
O	13.9293	18.6599	4.3967
O	23.3208	11.0992	6.9421
O	21.7136	6.7414	-0.3470
O	21.9825	4.2216	0.5064

Si	18.9517	8.2741	-2.3113
Si	17.4557	8.2914	5.2585
Si	15.8951	8.2983	12.7930
Si	7.9924	15.1055	5.2324
Si	18.5417	8.2746	2.2400
Si	16.9932	8.2352	9.7633
Si	9.0811	15.1292	2.2453
Si	18.5133	5.2130	2.2159
Si	16.9704	5.2039	9.7650
Si	9.0514	11.9769	2.2454
Si	7.5365	11.9725	9.7810
Si	17.4032	5.2731	5.2385
Si	15.8471	5.1862	12.7901
Si	7.9952	11.9959	5.2399
Si	20.1324	8.2909	6.8324
Si	12.2232	15.1495	-0.7887
Si	10.6728	1.5173	6.7174
Si	10.7091	15.1312	6.7754
Si	15.7962	8.3287	0.7349
Si	14.2614	8.3196	8.2468
Si	15.7868	5.1720	0.7103
Si	14.2593	5.2180	8.2100
Si	20.1098	5.1869	6.7948
Si	12.2175	11.9875	-0.7992
Si	10.7122	12.0212	6.7706
Si	13.3904	8.2737	2.7659
Si	11.9907	8.2839	10.3769
Si	22.3663	8.3057	4.6572
Si	13.0271	1.4651	4.5952
Si	12.9734	15.1415	4.6548
Si	22.3940	5.1902	4.6533
Si	14.5806	12.0438	-2.8989
Si	12.9046	12.0680	4.5565
Si	11.4861	12.0378	12.1743
Si	13.4163	5.2253	2.8261
Si	11.9737	5.2089	10.3558
Si	22.8601	12.0773	2.8187
Si	21.2975	12.0737	10.3936
Si	16.0842	9.5144	-2.0622
Si	13.0170	9.4920	13.0102
Si	21.3327	9.5335	2.0011
Si	19.7883	9.5486	9.5755
Si	11.8973	2.6615	2.0016
Si	11.9607	16.3352	1.9836
Si	10.3850	2.7039	9.5598
Si	10.4286	16.3630	9.5433
Si	19.7918	3.9583	9.5740
Si	11.8913	10.8798	1.9683
Si	10.4165	10.7976	9.5271
Si	14.5608	4.0535	5.4162

Si	14.5972	17.6185	5.4917
Si	13.5065	18.8561	2.8145
Si	11.5129	15.1090	12.2079
Si	7.5407	15.1072	9.7826
Si	23.9600	10.8105	5.4630
Si	23.6617	12.0130	8.2713
Si	21.6664	8.3218	-0.7579
Si	21.3439	3.9490	1.9919
Si	14.5867	15.1353	-2.9028
Si	21.5988	5.1648	-0.7922
H	19.4026	8.4671	-3.7162
H	18.5240	6.8662	-2.0429
H	17.0004	8.6838	13.6942
H	6.8545	15.9873	5.5276
H	7.9801	15.4407	1.3089
H	7.9416	11.6891	1.3278
H	6.4357	11.6334	8.8286
H	7.1212	11.8012	11.2083
H	16.9769	4.8216	13.6967
H	14.6467	4.3323	12.9885
H	6.8562	11.1049	5.5272
H	10.8538	15.4747	-1.2453
H	9.3221	1.9136	6.2326
H	10.7752	0.0684	7.0319
H	15.4877	4.3481	-0.4740
H	10.8617	11.6401	-1.2706
H	13.4816	0.0682	4.8450
H	23.5472	4.3421	5.0264
H	14.2215	11.8386	-4.3223
H	10.3164	11.7700	13.0576
H	12.3155	5.0252	11.7876
H	24.0145	11.7154	1.9477
H	22.4014	13.4883	2.6334
H	21.6505	11.7829	11.8149
H	20.9011	13.4998	10.1836
H	15.4490	8.6179	-3.0621
H	12.4585	9.2566	14.3484
H	20.3839	8.6653	10.6022
H	10.4208	2.4807	2.0229
H	12.4846	2.4812	0.6394
H	8.9003	2.5096	9.5179
H	11.0383	1.8353	10.5822
H	10.7170	17.7683	9.8865
H	20.3486	4.9399	10.5611
H	20.1197	2.5491	9.9166
H	16.0896	17.7575	5.4838
H	14.0002	17.9047	6.8310
H	14.6628	18.4837	1.9437
H	13.0621	20.2684	2.6304
H	10.2841	15.2925	13.0396

H	12.6213	16.0184	12.6148
H	6.4557	15.4559	8.8139
H	7.1035	15.2788	11.2020
H	25.4349	10.9777	5.4930
H	25.0138	11.6409	8.7658
H	23.5558	13.4494	7.8944
H	22.6678	8.6178	-1.7957
H	21.5847	2.5494	2.3947
H	14.1810	15.2692	-4.3312
H	15.7161	16.0360	-2.5354
H	20.1939	4.8825	-1.2351
H	22.5949	4.8982	-1.8704

	210 atoms	Al4-O8	
Energy =	-24863.53662900	Hartree	
H	15.0206	11.5050	4.8574
Al	14.3636	9.2823	5.4945
O	16.0823	9.3633	5.3713
O	13.6611	9.2376	7.0566
O	13.4772	8.3537	4.3664
O	14.1536	11.0770	4.9818
O	19.9452	8.6421	-1.2474
O	17.5101	9.2071	-1.9334
O	18.3820	8.4935	6.3601
O	17.9540	8.3864	3.7615
O	16.5935	6.7651	5.3887
O	14.4984	9.2456	12.8795
O	16.2490	8.3356	11.1164
O	15.2352	6.6929	12.9638
O	9.0492	15.1159	6.3513
O	8.5968	15.4335	3.7502
O	7.4145	13.4112	5.0748
O	17.0638	8.5802	1.2325
O	19.4823	9.3068	1.8231
O	18.8684	6.7252	1.9091
O	15.6669	8.3513	8.5253
O	17.9797	9.2488	9.3556
O	17.4908	6.6502	9.4720
O	10.2675	15.9327	1.7737
O	9.4059	13.4340	2.1702
O	17.1361	4.8347	1.1833
O	19.5102	4.1467	1.9294
O	17.8467	5.0831	3.7504
O	15.6516	4.9483	8.5202
O	17.9757	4.0636	9.3879
O	16.2032	4.9777	11.1132
O	10.2600	10.9682	1.7120
O	8.6075	11.4124	3.7340
O	8.6950	10.8002	9.3558
O	7.9562	13.4006	9.4421
O	18.3572	4.9910	6.3491
O	16.0348	4.1691	5.4462
O	9.0623	11.6935	6.3261
O	20.3056	9.2138	8.0259
O	20.8753	8.7238	5.4873
O	20.2410	6.7288	7.1349
O	12.5516	15.9625	0.4752
O	13.1701	15.2613	-1.9425
O	12.1211	13.4326	-0.2464
O	10.9441	2.3921	7.9971
O	11.6661	1.8857	5.4840
O	11.0098	15.9002	7.9595
O	11.5196	15.4293	5.4107

O	10.9811	13.4057	7.0627
O	15.2023	9.1570	-0.5673
O	14.5441	8.6648	1.9581
O	15.3855	6.6928	0.3361
O	13.2572	8.5892	9.5871
O	13.7533	6.6773	7.7598
O	14.6081	4.6828	1.9412
O	13.7655	4.1663	6.8601
O	13.1505	4.6534	9.3826
O	20.2958	4.2455	7.9997
O	20.8419	4.7294	5.4554
O	12.5640	10.9098	0.4512
O	13.1543	11.6468	-1.9824
O	10.9816	10.9131	7.9746
O	11.5613	11.3898	5.4329
O	11.9607	9.1001	2.3240
O	12.8390	6.6449	2.4131
O	12.1523	8.2187	11.9209
O	10.6451	9.1608	9.9312
O	11.4118	6.6670	9.9090
O	21.6915	8.3404	3.0143
O	23.4187	9.1862	4.8741
O	22.5764	6.7069	4.9006
O	12.4817	1.5274	2.9725
O	14.1711	2.4642	4.8402
O	12.5239	15.1185	2.9752
O	14.1336	15.8871	4.9858
O	13.2387	13.4499	4.8532
O	21.8372	4.9686	3.0124
O	15.7171	10.9796	-2.4374
O	15.0499	13.4383	-2.6604
O	12.5122	11.6779	2.9659
O	10.8855	11.7964	10.4712
O	12.5724	10.8616	12.3073
O	11.9633	13.4066	12.2844
O	13.7329	5.0844	4.3950
O	12.0598	4.1719	2.5149
O	10.5227	4.2642	9.8640
O	23.0778	11.8244	4.3826
O	21.3649	10.9676	2.4947
O	22.3226	11.5355	9.3432
O	19.7489	11.0374	9.8922
O	21.8206	9.2111	0.5471
O	12.0880	17.7021	2.4444
O	8.7283	15.9711	9.3764
O	11.0273	15.1707	10.5060
O	13.7976	18.5045	4.3719
O	23.0608	10.9508	6.8655
O	21.4863	6.6899	-0.3039
O	21.7541	4.1521	0.4949

Si	18.7514	8.2091	-2.2707
Si	17.2407	8.2712	5.2197
Si	15.7344	8.2196	12.6470
Si	7.9363	14.9624	5.1644
Si	18.3235	8.2415	2.1965
Si	16.8349	8.1450	9.6201
Si	9.0078	15.0183	2.2299
Si	18.3312	5.2120	2.2133
Si	16.8172	5.1644	9.6265
Si	9.0005	11.8498	2.2138
Si	7.4754	11.8420	9.6791
Si	17.2088	5.2597	5.2302
Si	15.6917	5.1438	12.6527
Si	7.9366	11.8583	5.1526
Si	19.9433	8.2845	6.7442
Si	12.1055	14.9861	-0.7637
Si	10.5864	1.5239	6.6536
Si	10.6318	14.9560	6.6975
Si	15.5468	8.2644	0.7481
Si	14.0700	8.2209	8.2184
Si	15.6041	5.1201	0.7235
Si	14.0802	5.1274	8.1328
Si	19.9284	5.1827	6.7258
Si	12.1025	11.8823	-0.7805
Si	10.6344	11.8536	6.7092
Si	13.2255	8.1751	2.8023
Si	11.8700	8.1625	10.3163
Si	22.1326	8.2343	4.5755
Si	12.9218	1.4530	4.5401
Si	12.8430	15.0048	4.5606
Si	22.1717	5.1542	4.5987
Si	14.4806	11.9290	-2.8737
Si	12.7766	11.9424	4.5278
Si	11.3646	11.9118	12.0321
Si	13.3156	5.1513	2.8225
Si	11.8457	5.1308	10.2478
Si	22.6330	11.9607	2.8107
Si	21.0566	11.9403	10.2872
Si	15.9081	9.4200	-2.0205
Si	12.8806	9.3537	12.8502
Si	21.0905	9.4526	1.9831
Si	19.5862	9.4695	9.4752
Si	11.7844	2.6413	1.9899
Si	11.8605	16.1825	1.9282
Si	10.2805	2.6953	9.4659
Si	10.3422	16.1835	9.4310
Si	19.6097	3.9275	9.4611
Si	11.8355	10.6443	1.8762
Si	10.3109	10.6689	9.4435
Si	14.4219	3.9789	5.3820

Si	14.4546	17.4455	5.4328
Si	13.3711	18.6702	2.7980
Si	11.4087	14.9358	12.0857
Si	7.4797	14.9589	9.6786
Si	23.7127	10.7088	5.3913
Si	23.4079	11.8735	8.1802
Si	21.4408	8.2646	-0.7265
Si	21.1236	3.9216	1.9786
Si	14.4668	14.9504	-2.8747
Si	21.3822	5.1203	-0.7748
H	19.1919	8.4229	-3.6734
H	18.3212	6.8115	-2.0208
H	16.7876	8.6250	13.5709
H	6.7982	15.8320	5.4383
H	7.9433	15.3420	1.2891
H	7.9344	11.4988	1.2884
H	6.4161	11.5063	8.7105
H	7.0485	11.6821	11.0963
H	16.7930	4.7635	13.5756
H	14.4919	4.3121	12.8080
H	6.7868	10.9992	5.4372
H	10.7795	15.3125	-1.3002
H	9.2659	1.9342	6.1355
H	10.6482	0.0808	7.0154
H	15.3477	4.3009	-0.4545
H	10.7784	11.5300	-1.3147
H	13.3231	0.0743	4.7486
H	23.3112	4.3156	4.9744
H	14.0175	11.6650	-4.2305
H	10.2235	11.6554	12.9395
H	12.2154	4.9386	11.6514
H	23.7624	11.6097	1.9090
H	22.1547	13.3339	2.6775
H	21.4773	11.6124	11.6542
H	20.6656	13.3441	10.0904
H	15.3088	8.5647	-3.0595
H	12.3322	9.1241	14.1653
H	20.2000	8.5688	10.4779
H	10.3248	2.5198	2.0236
H	12.3908	2.3875	0.6749
H	8.8152	2.5262	9.4402
H	10.9549	1.8387	10.4646
H	10.5724	17.5814	9.7743
H	20.1949	4.8360	10.4920
H	19.8183	2.5132	9.7444
H	15.9163	17.5935	5.3920
H	13.8474	17.7234	6.7466
H	14.5040	18.3301	1.8947
H	12.8855	20.0347	2.6824
H	10.1914	15.1313	12.9221

H	12.5165	15.8314	12.4544
H	6.4209	15.3207	8.7079
H	7.0496	15.1494	11.0939
H	25.1600	10.8822	5.3983
H	24.7141	11.4543	8.7175
H	23.3548	13.3262	7.8395
H	22.4007	8.5817	-1.7706
H	21.3361	2.5301	2.3417
H	13.9541	15.1068	-4.2317
H	15.5692	15.8247	-2.5060
H	20.0062	4.8284	-1.2879
H	22.4362	4.8265	-1.7499
